# Biomarkers and Tourette syndrome: a systematic review and meta-analysis

**DOI:** 10.3389/fneur.2024.1262057

**Published:** 2024-02-07

**Authors:** Yanlin Jiang, Yuan Li, Xi Chen, Rui Zhai, Yaqi Peng, Ran Tai, Congxiao Zhou, Junhong Wang

**Affiliations:** Department of Pediatrics, Dongzhimen Hospital, Beijing University of Chinese Medicine, Beijing, China

**Keywords:** Tourette syndrome, biomarkers, diagnosis, meta-analysis, children

## Abstract

**Objective:**

This research aims to investigate whether peripheral biomarkers might differentiate individuals with Tourette syndrome (TS) from those without the condition.

**Methods:**

A broad range of databases was searched through November 2022. This study employed a systematic literature review and subsequent meta-analysis of case-control studies that assessed the aberration of biomarkers of patients with TS and controls.

**Results:**

A total of 81 studies were identified, out of which 60 met the eligibility criteria for inclusion in the meta-analysis. Following a meticulous screening procedure to determine the feasibility of incorporating case–control studies into the meta-analysis, 13 comparisons were statistically significant [CD3+ T cell, CD4+ T cell, CD4+ T cell to CD8+ T cell ratio, NK-cell, anti-streptolysin O antibodies, anti-DNase antibodies, glutamic acid (Glu), aspartic acid (Asp), ferritin (Fe), zinc (Zn), lead (Pb), vitamin D, and brain-derived neurotrophic factor (BDNF)]. Publication bias was found for anti-streptolysin O antibodies. Suggestive associations were evidenced for norsalsolinol (NSAL), neuron-specific enolase (NSE), and S100B.

**Conclusion:**

In this study, we present empirical evidence substantiating the link between several peripheral biomarkers and the early diagnosis of TS. Larger and more standardized studies are necessary to replicate the observed results, elucidate the specificity of the biomarkers for TS, and evaluate their precision for use in clinical settings.

## Introduction

Tourette syndrome (TS), as one of the most prevalent childhood-onset neuropsychiatric disorders, is characterized by the presence of multiple motor tics and at least one vocal tic, persisting for a minimum of 1 year (1). Tics are defined as “sudden, rapid, recurrent, non-rhythmic motor movement or vocalization” (2). The global prevalence of TS in children and adolescents is estimated to be 0.7%, signifying its substantial impact on public health (3). Comorbidities commonly co-occur in TS patients, encompassing attention-deficit hyperactivity disorder (ADHD), anxiety, obsessive-compulsive disorder (OCD), learning difficulties, or other behavioral challenges (4). While some tics might exhibit mild characteristics, others can lead to psychosocial, physical, and functional difficulties that significantly influence social interactions, academic accomplishments, and job performance (5).

Evaluating and treating TS is still complex (6) partly due to an unclear etiology and diagnoses based on sets of signs and symptoms. To date, there are no established gold standards employing biological tests to definitively validate psychiatric diagnoses (7), including TS. A biomarker is defined as “a distinct characteristic that is quantified as an indicator of typical biological processes, pathological processes, or responses to an exposure or intervention” (8). The detection of peripheral biomarkers, accessible through noninvasive *in vivo* measurements, has the potential to aid in distinguishing TS from other conditions and contribute to the development of individual treatment.

In this review, we aimed to clarify and quantify the correlation between peripheral biomarkers and TS. To meet this objective, we conducted a systematic review and subsequently performed a meta-analysis encompassing studies assessing the association between TS and biomarkers in the following domains: immune processes (immune cells, antibodies, complement and cytokines); neurotransmitters, including monoamine neurotransmitters and amino-acid neurotransmitters; nutritional factors (trace elements and vitamins); hypothalamic-pituitary-adrenal axis (HPA) alterations; and markers implicated in other aspects of brain functioning (neurotrophic factors and prolactin). The findings from the reviewed data and our meta-analysis outcomes are thoroughly discussed in this article.

## Methods

The present review adhered to the guidelines outlined in the Preferred Reporting Items for Systematic Reviews and Meta-Analyses (PRISMA) (9). The review protocol was duly registered on PROSPERO (registration number: CRD42023391034).

### Literature search

To conduct the review and meta-analysis, a comprehensive search was performed across five electronic databases [Medline/PubMed; Cochrane Library; Embase; Web of Science; the China National Knowledge Infrastructure (CNKI)], from their inception until November 2022, for all eligible studies for the association between biomarkers and TS in childhood. A search algorithm based on a combination of terms: (tic disorders OR tics OR Tourette OR Tourette Syndrome) AND (Serum OR Plasma OR Urine OR Saliva OR Blood OR Blood Platelets OR Erythrocytes OR Hair OR levels OR peripheral OR cerebrospinal fluid OR red blood cells OR salivary biomarker* OR urinary biomarker* OR plasma biomarker* OR blood biomarker* OR serum biomarker* OR biomarker*) was used. The search strings are described in Supplementary Table S1. Systematic exploration of the reference lists of articles was conducted to identify additional relevant publications.

### Inclusion and exclusion criteria

Eligible studies were population-based investigations that compared the occurrence of one or more of the peripheral biochemical markers (as elucidated in the introductory section of this manuscript) of clinically diagnosed cases of TS versus healthy controls (unrelated to cases). We included studies that investigated children under 18 years and used categorical TS diagnosis criteria according to the International Classification of Diseases (ICD) manual, the Diagnostic and Statistical Manual of Mental Disorders (DSM), or less universally applicable criteria, such as Chinese Classification and Diagnostic Criteria of Mental Disorders (CCMD). In addition, studies that met the inclusion criteria should provide statistics required for meta-analysis (or where data were retrievable from the authors). Comorbid OCD or ADHD was included in the analysis due to its relative significance among the three disorders. However, studies that combined the results of OCD, ADHD, and tic disorders (TD) without explicitly specifying the inclusion of patients diagnosed solely with TS were excluded from consideration.

Exclusion criteria encompassed case reports or reviews, articles not published in English or Chinese, and studies not involving human participants or selecting samples based on disorders other than TS. We did not include potential genetic biomarkers because of different analytical methods. We also did not include potential biomarkers from neuroimaging studies because we would mainly focus on detection of peripheral biomarkers through biological fluid. Meta-analyses were conducted for all biomarkers with available data that were reported in a minimum of three published studies.

### Data extraction and quality assessment

Two investigators individually retrieved information on the first author’s name, the population year, biological fluid type, sample size, diagnostic criteria used to diagnose TS, percentage of participants, mean age in years, and biomarkers examined from each eligible article. Biomarkers were presented as concentrations with mean (standard deviation, SD), median (interquartile range, IQR), or median (range), and the data of the latter were transformed to the former by particular formulae (10–12) on the website.^1^ We combined mean and SD from multiple groups into a single group by using Cochrane’s Formula (13, 14) on the website,^2^ if necessary. Study quality was rated using the Newcastle–Ottawa scale (NOS) (15), as recommended by the Cochrane Collaboration (14). Studies with NOS scores ranging from 7 to 9 were categorized as high quality, scores from 4 to 6 as medium quality, and scores less than 4 as low quality (Supplementary Table S3).

### Statistical analyses

We employed R software version 4.1.2. for statistical analysis using the “meta” package. The standardized mean difference (SMD) was computed as the effect size (ES) in each eligible study to facilitate meta-analysis of continuous data, given the variability in measurement methods for different biochemical parameters. For categorical data involving studies assessing the positivity of specific antibodies with cutoff values, we conducted meta-analysis using Mantel–Haenszel method and pooled the risk ratio (RR). The evaluation of the links between different peripheral biomarker levels and TS was conducted using ESs and their 95% confidence intervals (CIs). Sensitivity analyses were performed using leave-one-out method to ascertain whether any individual study significantly influenced the results. Between-study heterogeneity was assessed using *χ*^2^ test of goodness of fit test and *I*^2^ statistic. In instances where obvious heterogeneity (*I*^2^ is greater than 50%) (16) was observed among the studies, we employed a random-effects model in our meta-analysis. Conversely, a fixed-effects model was utilized when no substantial heterogeneity was detected. Publication bias was quantified by Egger’s test and visualized by funnel plot when the usable data of biomarker levels were reported in at least 10 published studies (17). *p*-values of 0.05 or less were considered significant.

## Results

**Figure 1:**
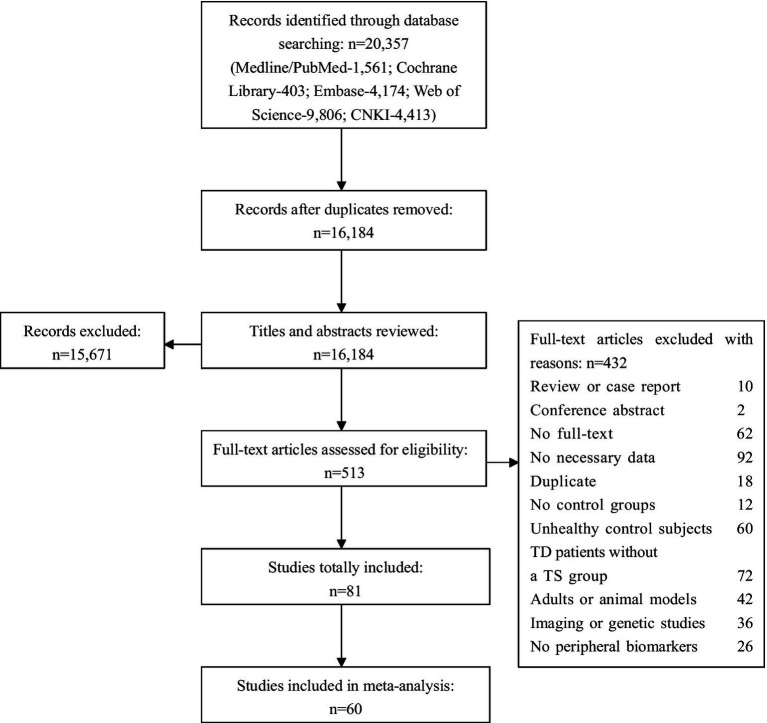
Flow chart depicting the selection procedure for review and meta-analysis.

The initial database search yielded 20,357 articles. Among them, 4,173 records were removed due to duplication and 15,671 articles were excluded after reviewing the titles and abstracts, leaving 513 papers with full-text available during the screening process. Finally, basing on the inclusion and exclusion criteria, we retained 81 studies for the systematic review and meta-analysis (a total of 8,313 participants including 3,842 with TS and 4,471 comparison subjects). A flowchart summarizing the study selection process is presented in Figure 1.

A total of 39 studies focused on alterations in immune processes divided into immune cells (13 studies), antibodies (23 studies), complement (3 studies), and cytokines (12 studies). We found 23 studies of neurotransmitters including monoamine neurotransmitters (15 studies), amino-acid neurotransmitters (7 studies), and other neurotransmitters (4 studies). Thirteen studies reported nutritional factors (trace elements: 7 studies; vitamins 7 studies), and three studies analyzed biochemical alterations in the HPA pathway. We used the term “other” for nine studies that investigated biomarkers involved in other aspects of brain functioning [neurotrophic factors: 3 studies; prolactin: 3 studies; neuron-specific enolase (NSE): 2 studies; S100B protein: 1 study]. Nearly all included studies quantified biomarker concentrations in the serum or plasma of participants with TS.

Supplementary Table S2 provides a detailed account of the attributes of the case–control studies selected for inclusion in the meta-analysis. All subsequent analyses presented herein are founded upon SMD. According to the quality assessment results from our meta-analysis, 50 articles were designated as high quality, with 10 articles categorized as medium quality, and this classification was deemed satisfactory. The Egger’s test and funnel plots of the data from the eligible studies indicated publication bias for anti-streptolysin O antibodies (Supplementary Figure S8), suggesting that these results may not be robust enough. In addition, the results of sensitivity analyses are presented in Supplementary Figures S9–S13.

### Immune system

Out of the 39 case-control studies involved, 31 studies were subjected to meta-analysis for immune cells, antibodies, complement, and cytokines.

#### Immune cells

**Figure 2:**
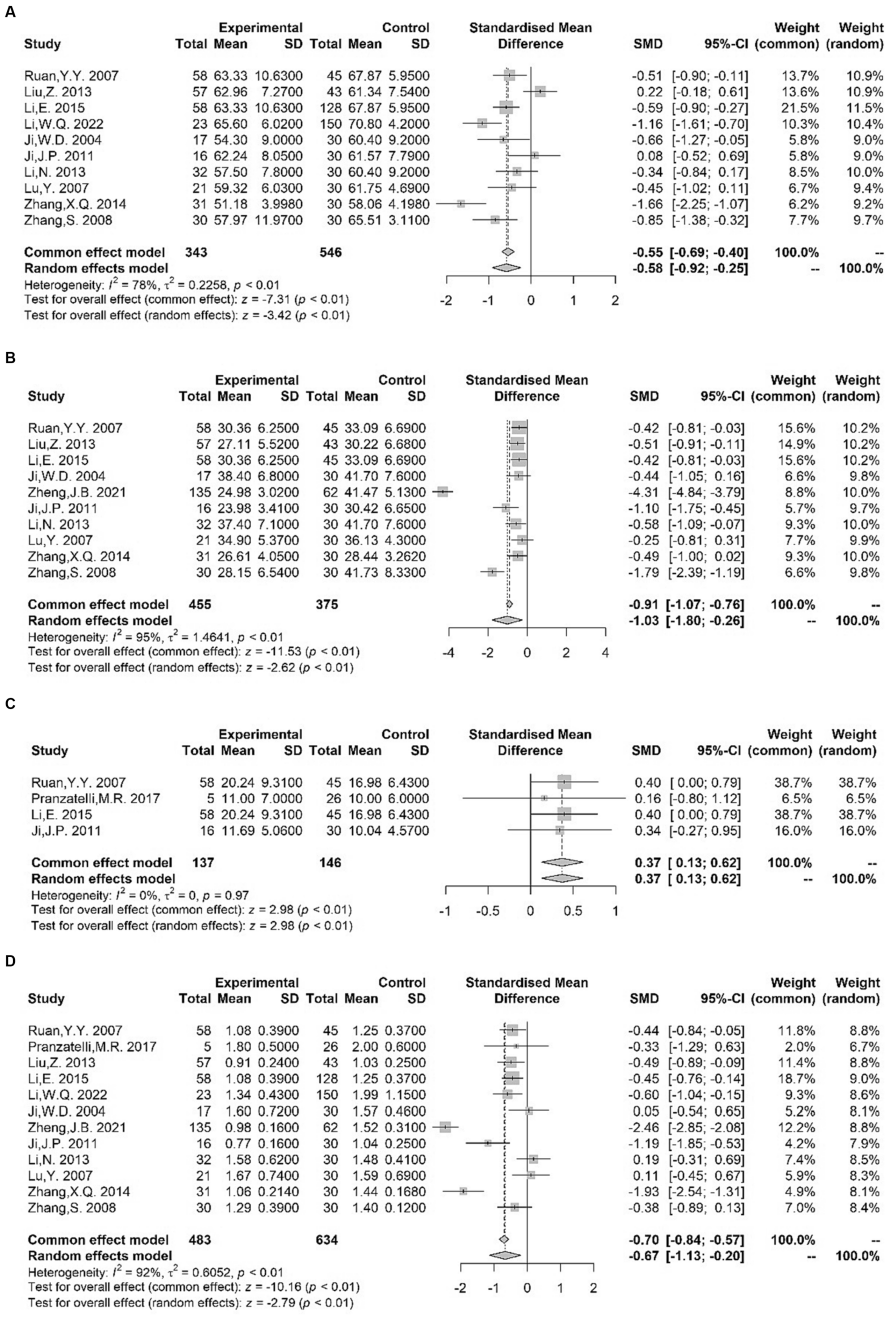
Forest plots for standard mean difference (SMD) from meta-analysis of serum CD3^+^ T cell **(A)**, CD4^+^ T cell **(B)**, NK cell **(C)** levels and CD4^+^ T cell to serum CD8^+^ T cell ratio **(D)**.

The serum levels of specific B or T clusters of differentiation (CD) tagging lymphocyte cell subpopulations were investigated in several studies. We found significantly lower CD3+ T cell levels (18–27) [SMD = −0.58 (−0.92, −0.25); *I*^2^ = 78%; *p* < 0.01], lower CD4+ T cell levels (18–20, 22–28) [SMD = −1.03 (−1.80, −0.26); *I*^2^ = 95%; *p* < 0.01], and higher NK cell levels (18, 20, 23, 29) [SMD = 0.37 (0.13, 0.62); *I*^2^ = 0%; *p* < 0.01] in children with TS compared with healthy controls (Figure 2). Differences in serum CD4+ T cell to serum CD8+ T cell ratio (18–29) were noted between patients and control groups, with obvious heterogeneity of effect sizes across the studies [SMD = −0.67 (−1.13, −0.20); *I*^2^ = 92%; *p* < 0.01] (Figure 2). However, our meta-analysis found no significant variations between TS and controls to the levels of CD8+ T cell (18–20, 22–27) [SMD = 0.05 (−0.29, 0.39); *I*^2^ = 75%; *p* = 0.78] or CD19+ lymphocytes (18, 20, 30) [SMD = 0.64 (−0.56, 1.83); *I*^2^ = 96%; *p* = 0.30] (Supplementary Figure S1).

Li et al. (21) found decreased serum CD3+ CD4+ T cell percentages in children with TS compared with healthy controls, whereas no alterations were observed in the serum levels of CD3+ CD8+ T cell. Given its direct contact with the brain’s extracellular space, the classical cerebrospinal fluid (CSF) mirrors biochemical modifications occurring within the brain (31). According to a single study, the percentages of CSF total conventional T cells, B cells, or NK cells did not display significant group abnormalities (29).

Considering the observed disparities, it is not possible to reach a definitive conclusion based on the available data, indicating that immune cells can differentiate patients with TS from those without TS.

#### Antibodies

The meta-analysis of blood antibodies encompassed 15 eligible studies. Based on the quantitative meta-analysis of the gathered studies, there were no discernible variations in the levels of the listed immunoglobulins (Ig) (Supplementary Figure S2) between patients diagnosed with TS and control subjects: IgA (25, 27, 32, 33) [SMD = −0.01 (−0.44, 0.41); *I*^2^ = 67%; *p* = 0.95], IgM (25, 27, 32, 33) [SMD = −0.04 (−0.48, 0.40); *I*^2^ = 68%; *p* = 0.86], and IgG (25, 27, 32, 33) [SMD = −0.21 (−0.45, 0.02); *I*^2^ = 31%; *p* = 0.08]. Similar results for CSF concentrations of Ig were found (29), although we did not have sufficient data for meta-analysis. Landau et al. (32) found a significant increase in serum IgE levels within the TS group when compared to controls, but this outcome was not replicated by Yuce et al. (34) Moreover, Singer et al. (35) reported that TS patients had higher serum levels of antineuronal antibodies (ANAb) against putamen than the controls; by contrast, no differences were observed in the levels of serum antibodies against neuron-like HTB-10 neuroblastoma cells (36) or plasma antiphospholipid antibodies (aPLAs) (37).

**Figure 3:**
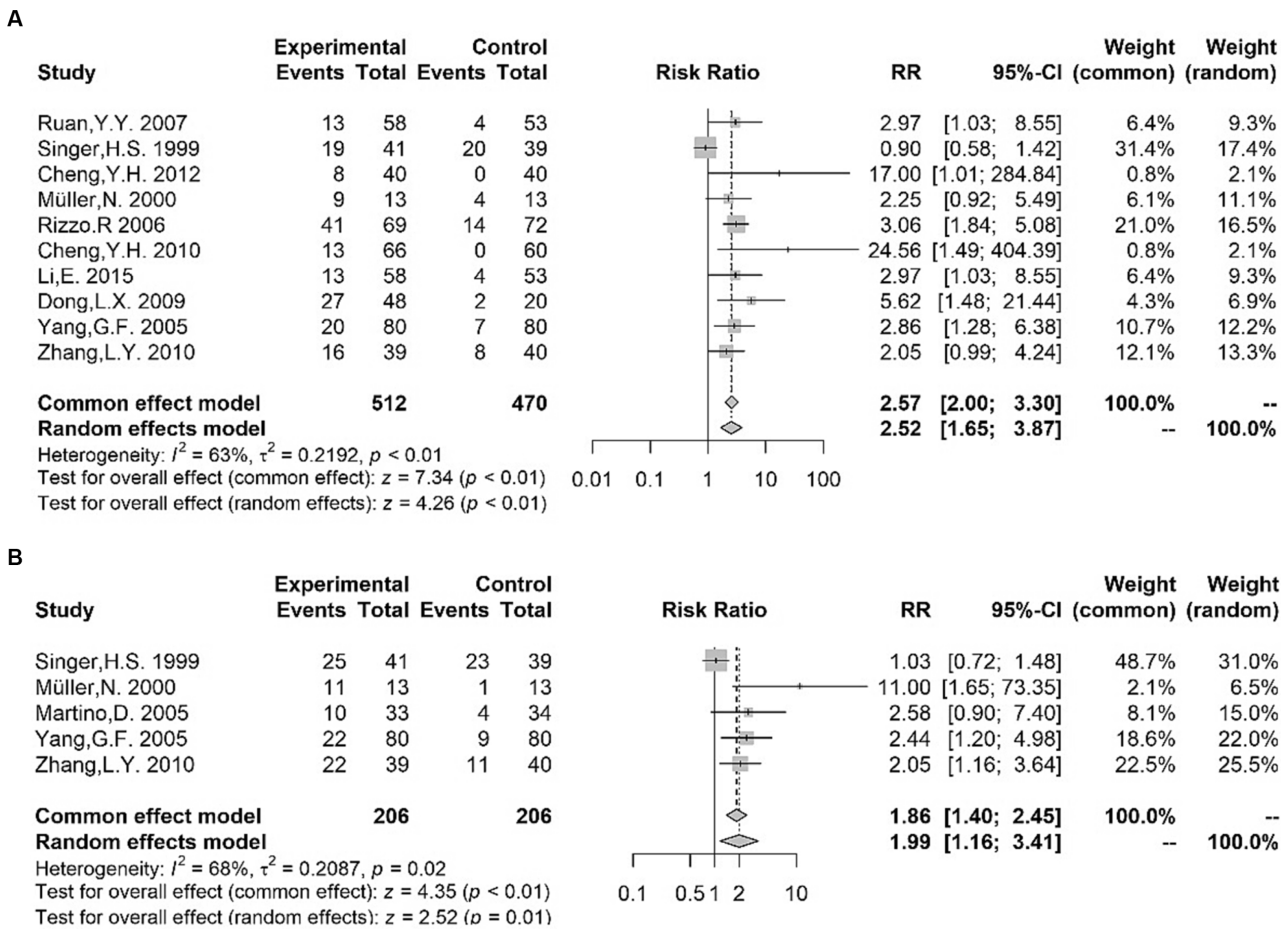
Forest plots for risk ratio (RR) from meta-analysis of anti-streptococcal antibody titers [anti-streptolysin O antibodies **(A)**; anti-DNase antibodies **(B)**].

Qualitative meta-analysis indicated that the positivity for the following antibodies was more frequent in children with TS than in healthy participants: anti-streptolysin O antibodies (18, 20, 36, 38–44) [RR = 2.52 (1.65, 3.87); *I*^2^ = 63%; *p* < 0.01]; anti-DNase antibodies (36, 40, 42, 43, 45) [RR = 1.99 (1.16, 3.41); *I*^2^ = 68%; *p* = 0.01] (Figure 3). Hence, the existing evidence allows us to suggest that Streptococcus might be a viable etiological candidate for TS, thereby supporting the PANDAS/PANS hypothesis (46, 47). The positivity of other autoantibodies was also examined in several studies. Concerning anti-basal ganglia antibodies (ABGA), a category of anti-neuronal antibodies connected with a diverse spectrum of post-streptococcal neuropsychiatric disorders (48), two studies (38, 42) found an increased rate of ABGA-positive subjects in TS patients compared with that in controls. Moreover, Cheng et al. (39) reported that significantly more patients in the TS group were positive for anti-brain antibodies (ABAb) and ANAb compared with the control group. However, no alterations were observed in the positivity of serum glial fibrillary acidic protein (GFAP) antibody between patients with TS and controls (49, 50). Although these findings need to be replicated, anti-*Streptococcus* antibodies and other autoantibodies mentioned above could be a potential biomarker of TS in light of the pathogenesis of neuroimmune interaction (51). In addition, two studies (52, 53) showed that the positive rate of *Mycoplasma pneumoniae* (MP) IgA in children with TS was higher than that in controls; hence, MP infection may be associated with Tourette syndrome, despite the limited evidence.

#### Complement

Complement, an essential component of the complement system made up of a battery of dozens of activators and inhibitors, was investigated in three studies. Our meta-analysis found no alteration in C3 (25, 27, 33) [SMD = −0.11 (−0.53, 0.31); *I*^2^ = 52%; *p* = 0.61] or C4 (25, 27, 33) [SMD = 0.22 (−0.07, 0.51); *I*^2^ = 0%; *p* = 0.14] concentrations between patients and controls (Supplementary Figure S3). Therefore, in the absence of further data, biomarkers associated with the complement system do not offer sufficient support to be considered as biomarkers for TS.

#### Cytokines

Eleven studies provided data for our meta-analysis. No significant distinction was observed between suffering from TS and control participants with regard to the levels of IFN-γ (28, 54–56) [SMD = 1.10 (−1.32, 3.53); *I*^2^ = 99%; *p* = 0.37], IL-1β (39, 54, 57) [SMD = 0.73 (−0.53, 1.98); *I*^2^ = 95%; *p* = 0.26], IL-4 (54–56) [SMD = 0.57 (−1.31, 2.45); *I*^2^ = 98%; *p* = 0.55], IL-6 (22, 24, 39, 57, 58) [SMD = 0.46 (−0.35, 1.28); *I*^2^ = 92%; *p* = 0.27], IL-8 (22, 24, 28, 58) [SMD = 0.50 (−1.76, 2.76); *I*^2^ = 99%; *p* = 0.66], IL-12 (54, 57, 59, 60) [SMD = 1.22 (−0.40, 2.84); *I*^2^ = 97%; *p* = 0.14], or TNF-α (57–60) [SMD = 1.89 (−0.28, 4.06); *I*^2^ = 98%; *p* = 0.09] (Supplementary Figure S4).

Several subsequent studies continued to search for the association between cytokines and TS. Two studies were conducted on serum IL-10, and the findings yielded divergent outcomes, with one study (61) reporting no alterations, while another study demonstrated reduced levels in children with TS (56). Zhang et al. (61) and Gao et al. (56) found significantly lower serum IL-2 levels in TS patients compared with controls. In addition, compared with controls, patients with TS had higher serum levels of IL-13 and IL-17, as confirmed by Cheng et al. (39) and Gao et al. (55)

In summary, the absence of compelling evidence regarding immune impairment hinders us from drawing a definitive conclusion that cytokines may be possible candidates to differentiate patients with TS from those without.

### Neurotransmitters

Twenty-three case-control studies of TS investigated on neurotransmitters. The studies suitable for meta-analysis consisted of eight for monoamine neurotransmitters [serotonin (5-HT): 5 studies; dopamine (DA): 8 studies; norepinephrine (NE): 6 studies] and seven for amino-acid neurotransmitters [gamma-aminobutyric acid (GABA): 4 studies; aspartic acid (Asp): 5 studies; glutamic acid (Glu): 6 studies].

### Monoamine neurotransmitters and their metabolites

#### 5-HT

Our meta-analyses pertaining to peripheral blood 5-HT levels (62–66) did not reveal any statistically significant distinctions between TS patients and controls [SMD = −0.97 (−5.21, 3.28); *I*^2^ = 98%; *p* = 0.66] (Supplementary Figure S5). Consistent with this result, one study of urine 5-HT found lower levels in TS (67). 5-Hydroxyindoleacetic acid (5-HIAA), the principal metabolite derived from serotonin, was observed in one study. The TS group was found to have significantly lower urine levels of 5-HIAA than the control groups (67). Zhao et al. (64) reported no alteration in tryptophan (the precursor of 5-HT) between patients with TS and controls. Moreover, Sallee et al. (68) reported that platelet 5HTPR capacity was reduced in children with OCD, but this reduction was not evident in individuals with TS.

In summary, although contrasting results were obtained in terms of the concentrations of 5-HT between children with TS and controls, and no definitive evidence was found for tryptophan, the 5-HT system is still considered a viable candidate due to genetic and gene expression studies implicating its role in the etiology of TS (69–71).

#### DA

Based on the results of our meta-analysis, no significant disparities were observed in the blood levels of DA (62–66, 72–74) between patients and controls [SMD = 1.78 (−0.45, 4.01); *I*^2^ = 98%; *p* = 0.12] (Supplementary Figure S5). Rabey et al. (75) employed an assay for quantifying DA accumulation within platelet storage granules (PSG); the data showed a diminished capacity of PSG to accumulate DA, which may be considered a physiological mechanism to compensate for the excessive DA activity. A pivotal role in the synaptic accumulation and quantal release of monoamines is carried out by the vesicular monoamine transporter (VMAT2). Ben-Dor et al. (76) reported a considerable reduction in platelet VMAT2 density in individuals with TS through the assessment of high affinity [3H] dihydrotetrabenazine binding to platelet VMAT2.

One study evaluated the presence of the DA metabolite homovanillic acid (HVA) in plasma and found increased levels in children with TS (62). For tyrosine (Tyr), the precursor of DA and NE, one study was conducted in plasma and showed negative results (64). Norsalsolinol (NSAL), a type of tetrahydroisoquinoline (TIQ), has the capacity to modulate dopaminergic neurotransmission and metabolism in the central nervous system. Capetian et al. (77) reported that NSAL concentrations in urine were elevated significantly in TS patients, which suggested that dopaminergic hyperactivity underlies the pathophysiology of TS. Hence, the concentrations of NSAL in urine have the potential to serve as a diagnostic biomarker for TS. β-endorphins, the neuromodulators of the brain, lead to excess accumulation of dopamine by inhibiting the release of GABA (78). However, one study was conducted for β-endorphins in CSF yielded a negative result (79).

Although these biomarker studies do not provide a definite correlation between DA and TS diagnosis, the DA system could still be a useful biomarker for TS given that genetic, animal, neuroimaging, and clinical studies have indicated the important role of DA in TS pathogenesis (4, 69, 80–82).

#### NE

Random-effect meta-analysis suggested that the levels of NE (62–65, 72, 74) in blood did not significantly differ between children with TS and controls [SMD = −0.07 (−0.69, 0.54); *I*^2^ = 92%; *p* = 0.82] (Supplementary Figure S5). 3-Methoxy-4-hydroxyphenylethylene glycol (MHPG) and normetanephrine (NM) are metabolites of NE. Baker et al. (83) reported that the urinary excretion of MHPG and NME was significantly lower in patients with TS than in controls. Considering the limited number of studies, we failed to see clearly the effects of NE and its metabolites on TS onset.

#### Biogenic trace amines

B-Phenylethylamine (PEA), derived from the decarboxylation of phenylalanine (Phe), is considered a “trace amine” due to its low urinary excretion rate and brain concentration compared with catecholamines. One study (84) examined the levels of PEA, Phe, and the PEA metabolite phenylacetic acid (PAA) in urine and/or plasma; statistical analyses revealed that patients with TS had lower plasma Phe and urinary PEA than the controls, but urinary/plasma PAA was not different between them. However, the available data are insufficient to definitively establish a link between abnormalities in the synthesis or metabolism of PEA and the etiology of TS.

#### Amino-acid neurotransmitters

**Figure 4:**
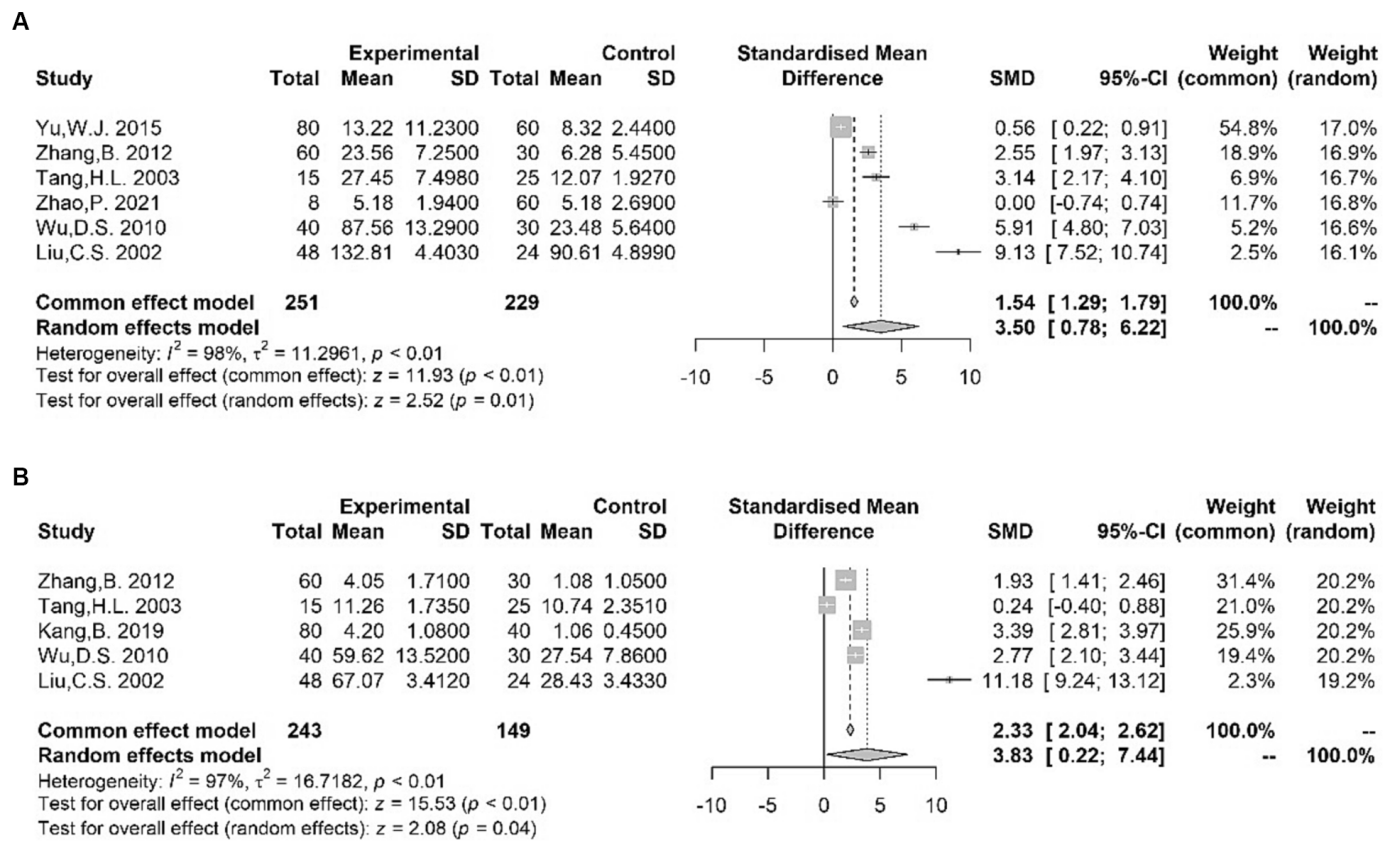
Forest plots for standard mean difference (SMD) from meta-analysis of blood Glu **(A)** and Asp **(B)** levels.

Our meta-analysis showed higher blood levels of Glu (62, 64, 74, 85–87) [SMD = 3.50 (0.78, 6.22); *I*^2^ = 98%; *p* = 0.01] and Asp (62, 65, 85–87) [SMD = 3.83 (0.22, 7.44); *I*^2^ = 97%; *p* = 0.04] in TS patients compared with normal subjects (Figure 4). However, our analysis did not reveal any statistically significant difference in the blood levels of GABA (62, 64, 65, 74) [SMD = −1.85 (−4.17, 0.47); *I*^2^ = 98%; *p* = 0.12] (Supplementary Figure S5) between cases and controls. Despite divergent data produced by different studies, amino-acid neurotransmitters still seem to be potential biomarkers for children with TS because of their important role in TS neurobiology (70, 88–91).

#### Other neurotransmitters

One study (92) reported no significant differences between CSF acetylcholinesterase (AChE) activity in patients with TS and controls. By contrast, Rabey et al. (93) reported a reduction in cholinergic muscarinic binding in peripheral lymphocytes, as evidenced by measurements of (3H) quinuclidinyl benzilate [(3H)-QNB], indicating the potential involvement of cholinergic receptor alterations in the pathophysiology of TS. Nitric oxide (NO), functioning as a neurotransmitter and acting on the NMDA receptor, is suggested to play a role in the pathogenesis of basal ganglia in TS. However, studies of NO levels reported contrasting results. In one study (94), there were no significant differences between patients with TS and controls, while another study by Hu et al. (95) reported higher plasma levels in patients compared to controls.

### Nutritional factors

#### Trace elements

**Figure 5:**
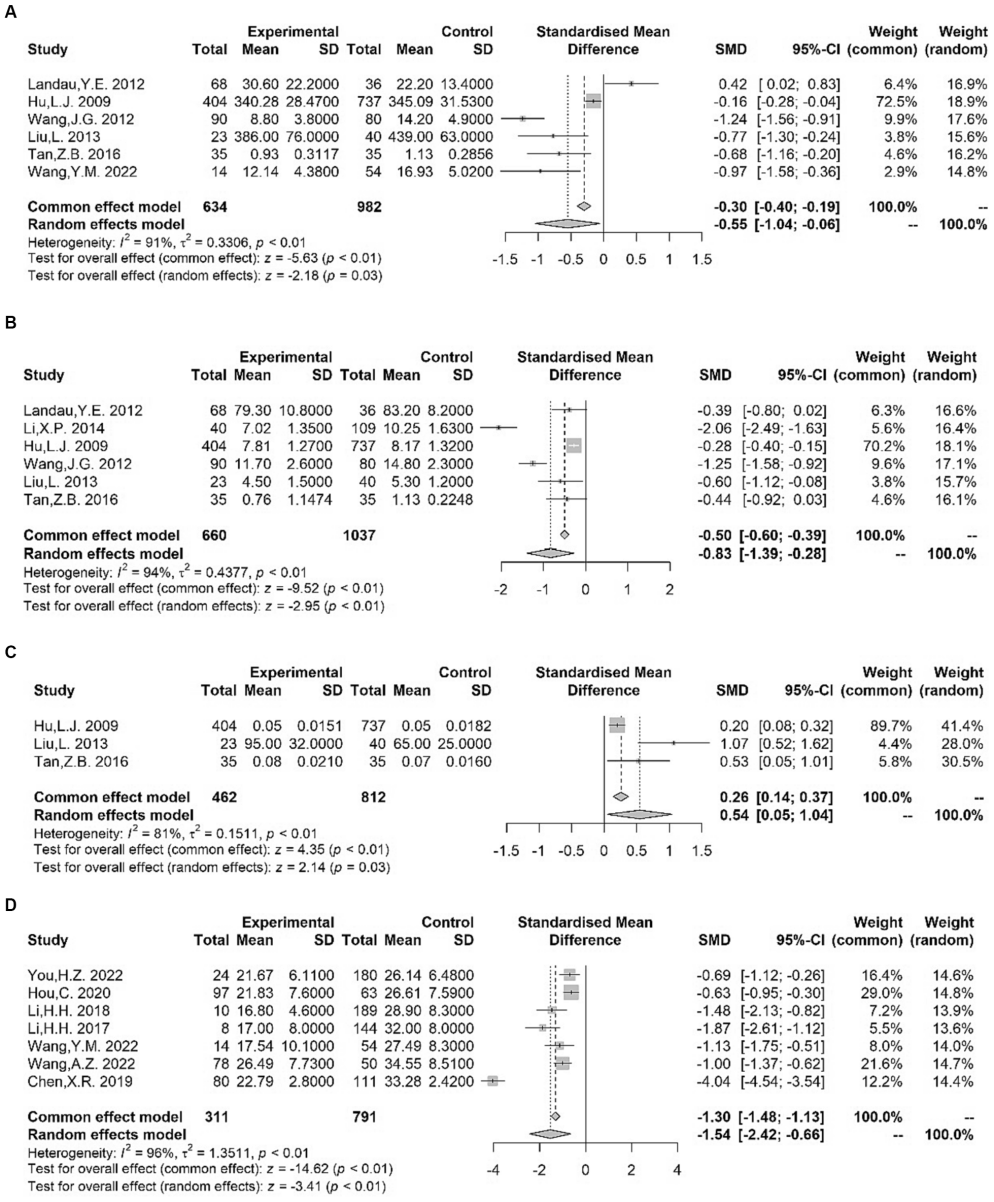
Forest plots for standard mean difference (SMD) from meta-analysis of blood Fe **(A)**, Zn **(B)**, Pb **(C)** and vitamin D **(D)** levels.

Seven studies that specifically assessed trace element status in children with TS were used for meta-analysis. In the TS group, the plasma/serum concentration of ferritin (Fe) (32, 96–100) [SMD = −0.55 (−1.04, −0.06); *I*^2^ = 91%; *p* = 0.03] and zinc (Zn) (32, 63, 96–99) [SMD = −0.83 (−1.39, −0.28); *I*^2^ = 94%; *p* < 0.01] was significantly lower compared to controls, as indicated by the results of our meta-analysis (Figure 5). However, in our meta-analysis, we found significantly higher serum lead (Pb) levels (96, 98, 99) among patients with TS [SMD = 0.54 (0.05, 1.04); *I*^2^ = 81%; *p* = 0.03] (Figure 5). Moreover, no alterations were observed in the blood levels of calcium (Ca) (63, 96, 97, 99) [SMD = −0.12 (−0.44, 0.20); *I*^2^ = 80%; *p* = 0.48], magnesium (Mg) (96, 98, 99) [SMD = 0.00 (−0.11, 0.12); *I*^2^ = 0%; *p* = 0.98], and copper (Cu) (96, 98, 99) [SMD = 0.06 (−0.05, 0.18); *I*^2^ = 18%; *p* = 0.28] between patients with TS and controls (Supplementary Figure S6). In addition, Liu et al. (98) reported no significant differences in serum manganese (Mn) levels between children with TS and control subjects. Exposure to high levels of heavy metals (lead et al.) and deficiencies for trace elements, such as Fe and Zn, may be connected with a clinical diagnosis of TS and increased risk of TS onset.

#### Vitamins

We examined seven studies of serum vitamin D. The pooled analysis demonstrated a significant reduction in serum vitamin D levels (100–106) among patients with TS [SMD = −1.54 (−2.42, −0.66); *I*^2^ = 96%; *p* < 0.01] (Figure 5). Although the number of eligible studies was limited, one study (105) provided further support for lower vitamin A levels in patients with TS compared to controls. However, a conflicting result was observed in the study by Hou et al. (102). Additionally, Wang et al. (105) reported no significant differences in the serum levels of vitamin E between the TS and control groups. Altogether, vitamin insufficiency, especially vitamin D deficiency, may contribute to an increased occurrence of tics.

### Hypothalamus-pituitary-adrenal axis pathway

For the meta-analysis of serum cortisol, three studies exploring the HPA axis were included. We found no difference between TS and control subjects in terms of the baseline serum levels of cortisol (28, 65, 107) [SMD = −0.34 (−3.31, 2.64); *I*^2^ = 99%; *p* = 0.82] (Supplementary Figure S7).

Despite the inadequate number of eligible studies for conducting a meta-analysis on adrenocorticotrophic hormone (ACTH) and TS, two studies (65, 107) demonstrated a significant rise in serum ACTH levels in the TS group when compared to controls.

Although initial studies on the physiological stress response, focusing on HPA axis activation, hinted at possible dysregulation in TS, the existing evidence for using HPA axis biological indicators as biomarkers for TS is still inadequate.

### Other

**Figure 6:**
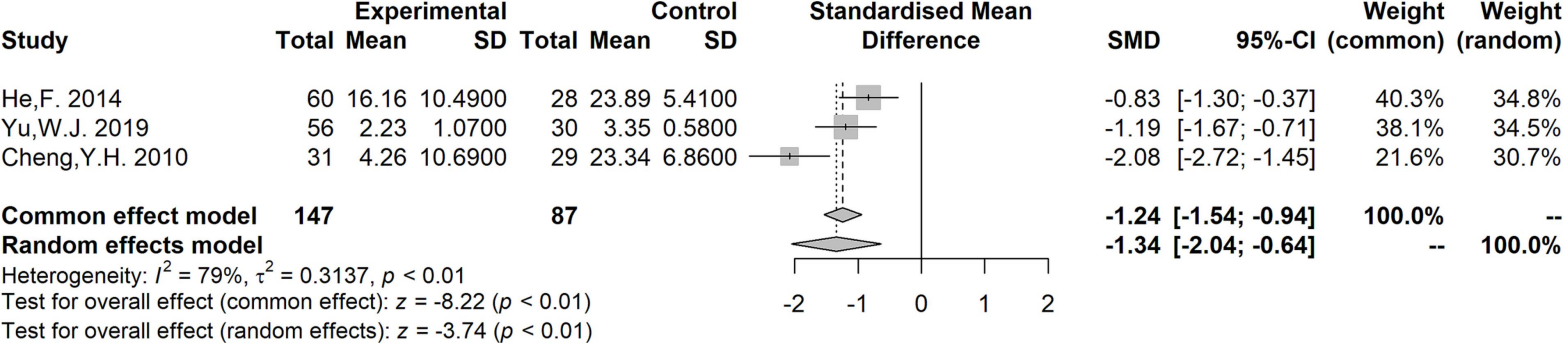
Forest plots for standard mean difference (SMD) from meta-analysis of serum BDNF levels.

The neurotrophin brain-derived neurotrophic factor (BDNF), one of the most widely investigated molecules in psychiatric disorders, is associated with neuronal maintenance, neuronal survival, plasticity, and neurotransmitter regulation (108, 109). The meta-analysis revealed diminished serum BDNF levels (110–112) in individuals with TS in comparison to the control group [SMD = −1.34 (−2.04, −0.64); *I*^2^ = 79%; *p* < 0.01] (Figure 6). The above results and the study of genetic susceptibility (113) suggest that BDNF could be a good candidate for TS biomarkers. Prolactin (PRL) is a peptide hormone produced and released from specialized cells called lactotrophs in the anterior pituitary gland (114). The levels of PRL (115–117) showed no statistically significant differences between TS patients and controls, according to the findings of the meta-analysis [SMD = −0.14 (−1.64, 1.36); *I*^2^ = 95%; *p* = 0.85] (Supplementary Figure S7). Moreover, two studies (21, 118) reported that the NSE levels increased in patients with TS, suggesting the potential utility of NSE as a biomarker in TS. Nevertheless, additional research is needed to validate this observation. In addition, Ruan et al. (119) found higher serum level of the S100B protein (a specific protein reflecting the degree of brain injury and prognosis) in patients with TS than in healthy children.

## Discussion

This extensive review aimed to evaluate biomarkers as potential diagnostic indicators for TS through a comprehensive review and meta-analysis of previous studies. Accessible fluid biomarkers can offer an objective assessment of the current situation, supporting TS diagnosis or assessing disease status and ultimately guiding clinical decisions. Our study demonstrated multiple significant variances of blood-derived parameter levels through comparisons between children with and without TS. Meanwhile, the results obtained from our study further corroborate the notion that both neurotransmitters and the immune system are dysregulated in TS; as such, lead exposure and trace elements or BDNF deficiencies may be risk factors. Biomarkers measured in peripheral samples could potentially serve as diagnostic tools, although further research is required before their implementation in clinical practice.

Our meta-analysis resulted in 13 significant findings, which are summarized in Table 1. Two of these were obtained from the qualitative analysis. Most biomarkers that showed significant differences between patients with TS and controls exhibited obvious heterogeneity in effect sizes across studies. These findings of heterogeneity could be explained by several factors, such as sample sources, cutoff positivity values, and study methodological procedures. They might also result from differences among the studies in terms of subject demographics, environmental exposures, life habits, nutritional status, or neuropsychiatric comorbidity. The significant heterogeneity indicated that TS is a complex and multifactorial disorder that results from a number of causative factors, with none being deemed essential or adequate for its initiation. This finding has been partially substantiated by genetic studies (4, 120, 121).

**Table 1:** Summary of the effect size difference meta-analysis findings.

Source	Biomarkers symbol	SMD/RR	*p*	Significant heterogeneity?	Quantitative meta-analysis?
Serum	CD3+	−0.58	<0.01	Yes	Yes
Serum	CD4+	−1.03	<0.01	Yes	Yes
Serum	CD4+/CD8+	−0.67	<0.01	Yes	Yes
Serum	NK-cells	0.37	<0.01	No	Yes
Serum/plasma	Glu	3.50	0.01	Yes	Yes
Serum/plasma	Asp	3.83	0.04	Yes	Yes
Serum	Fe	−0.55	0.03	Yes	Yes
Serum	Pb	0.54	0.03	Yes	Yes
Serum/plasma	Zn	−0.83	<0.01	Yes	Yes
Serum	Vitamin D	−1.54	<0.01	Yes	Yes
Serum	BDNF	−1.34	<0.01	Yes	Yes
Serum	Anti-DNase-positive	1.99	0.01	Yes	No
Serum/plasma	Anti-streptolysin O -positive	2.52	<0.01	Yes	No

The significant meta-analyses for T cells, NK-cells, and anti-*Streptococcus* antibody positivity suggest that patients with TS have immune disturbances, especially in the levels of immune cells and activity of some specific antibodies. Our investigation revealed reduced counts of CD3+ and CD4+ T cells in patients with TS, accompanied by a diminished CD4+ to CD8+ ratio. In contrast, there was an increase in the number of NK cells. However, when it comes to T cells specifically, an observational study revealed that patients experiencing symptom exacerbation might display heightened levels of T cells (122). The plausibility of a link between potential immune dysfunction and the initiation or progression of TS was identified in our study. Notably, immune dysregulation is also present in other neurological diseases, such as OCD (123). As such, we cannot conclude that immune cells could become candidates for TS biomarkers, despite the significant findings.

The hypothesis of β-hemolytic streptococcal infections triggering TS and specific psychiatric disorders through immune responses has been proposed (124). Although tics might occur or worsen after group A streptococcal (GAS) infections, GAS infection as risk-modifiers for TS remains controversial (125). The term “pediatric autoimmune neuropsychiatric disorders associated with streptococcal infections” (PANDAS) refers to a group of disorders in children characterized by the sudden onset of tics, along with manifestations of obsessive-compulsive behavior and personality changes, which are associated with streptococcal infections (126). Pediatric acute-onset neuropsychiatric syndrome (PANS), the later and more comprehensive version, has attracted considerable attention and sparked controversy. Population-based studies indicated a higher likelihood of prior streptococcal infection the onset of symptoms in patients with OCD or TS (127, 128). Moreover, Murphy et al. (129) reported that patients with significant fluctuations in tics/OCD symptoms had consistently elevated streptococcal titers compared to those with a stable or remitting course. However, caution is necessary in interpreting the results due to the presence of publication bias in studies examining anti-streptolysin O antibodies in TS patients and controls. In summary, the significance of anti-Streptococcus antibodies (anti-streptolysin O antibodies and anti-DNase B antibodies) in TS requires further investigation, as the causal relationship between Streptococcus and TS is yet to be conclusively demonstrated. It is plausible that the higher levels of anti-Streptococcus antibodies observed in TS patients might be a consequence of stress-induced vulnerability to infections rather than serving as a direct etiological factor for TS (130).

The results in Table 1 implicate serum/plasma Glu and Asp levels as potential biomarkers for TS. Neurophysiological, brain imaging, and postmortem studies have substantiated the pathological involvement of cortico-striatal-thalamo-cortical (CSTC) pathways in TS (131). Perturbations in the levels of excitatory amino acid neurotransmitters, like Glu and Asp, within the CSTC loop have been associated with the pathogenesis of TS (132). Glu, an excitatory neurotransmitter, is highly expressed in brain tissues and is regularly associated with nervous system abnormalities (133). Asp is involved in the synthesis and release of GABA and DA (134). Excitatory amino acid neurotransmitters, especially Glu, plays a crucial role in pathways associated with CSTC circuits and has significant interactions with dopaminergic systems (135). These findings provide substantial support for the potential involvement of excitatory amino acid neurotransmitters in TS etiology. Mahone et al. (91) conducted a study that indicated an increase in Glu and GABA levels within the CSTC loop, leading to improved selective motor inhibition in children with TS. Additionally, the study conducted on D1CT-7 mice revealed a direct association between Glu release in the CSTC loop and tic disorder behavior (136). However, elevated levels of serum Glu and Asp have also been found in children with ADHD (137). And magnetic resonance spectroscopy studies suggest a possible rise in Glu levels in the striatum among children with ADHD, OCD, and autism spectrum disorders (ASD) (138, 139). Taken together, although positive results were found in preliminary studies, we have to admit that more evidence should be provided before excitatory neurotransmitters can be used as biomarkers in the diagnosis of TS.

Table 1 also indicates that serum/plasma Zn, serum Fe, serum Pb, and serum vitamin D levels could be useful biomarkers for TS. Nearly all the studies corroborate the association of reduced levels of serum/plasma Zn, serum Fe, and serum vitamin D with TS. In the realm of nutrient metabolism, zinc (Zn) assumes an indispensable role as a crucial cofactor, supporting the development and maintenance of brain structure. Additionally, Zn plays a vital part in the synthesis and regulation of melatonin, the pineal hormone that exerts substantial influence over dopamine function, making it a noteworthy component in TS treatment (140). Serum ferritin stands out as the prevailing and highly sensitive indicator of overall body iron reserves. Decreased iron stores might play a role in the hypoplasia of the caudate and putamen, potentially elevating susceptibility to tic development or leading to more severe tics (141). Meanwhile, the levels of Zn and Fe have a connection with DA metabolism, and decreased Zn and Fe levels could potentially contribute to impaired dopaminergic transmission in children diagnosed with TS. High-level lead exposure may cause neurotransmitter alterations, such as NE. The increased NE turnover is implicated in hyperactivity disorders, such as ADHD and TS (142). Interestingly, studies have found that children with ADHD and ASD also exhibit lower blood levels of Zn and Fe similar to those in TS patients, along with a history of lead exposure or elevated blood Pb levels (143–149). By regulating the gene expression of the rate-limiting enzyme, tyrosine hydroxylase, Vitamin D influences the production of both DA and NE (150). Therefore, vitamin D deficiency might contribute to dopaminergic dysfunction in TS. Moreover, In TS, vitamin D exerts a substantial anti-inflammatory effect, impacting both cellular and humoral immune responses. Therefore, inadequate vitamin D levels may be associated with inflammation in TS. Conversely, an overactive immune system in TS might lead to heightened vitamin D consumption, contributing to its decreased levels (“reverse causation”) (151). It’s worth noting that vitamin D deficiency is also observed in other neurological and psychiatric disorders, such as ADHD, ASD, anxiety, depression, and schizophrenia (152–156). Given the potential modifiability of nutritional factors, their implication in TS holds particular importance for clinical application.

BDNF is a potential biomarker illustrated in Table 1. Belonging to the neurotrophic factor family, BDNF holds significant importance in supporting the development, maintenance, and protection of striatal neurons (157, 158) and has role in enhancing neuromuscular transmission excitation-contraction coupling (159). Furthermore, the nutritional impact of BDNF on dopamine neurons is noteworthy as it enhances the number of dopamine receptors in the brain, closely linked to TS pathogenesis (160). In addition, the BDNF gene’s high association with TS also implicates its involvement in other movement disorders, including ADHD and OCD (161).

Few studies were conducted on NSAL, NSE, and S100B, which could be additional potential biomarkers for TS. Hence, additional relevant studies are required to further investigate the subject.

There has been skepticism surrounding studies focusing on peripheral blood parameters. The question of whether serum/plasma levels can be indicative of CNS activity remains ambiguous. However, investigations have revealed that specific blood-brain barrier (BBB) transporters facilitate the transportation of intact neurotransmitters from the CNS to the periphery (162). Notably, certain biomarkers, such as BDNF serum levels, have been demonstrated to reflect alterations in the brain (163).

The clinical utility of biomarkers for TS remains elusive, with no biomarkers currently serving as reliable diagnostic tools. Our study adds significant value by identifying potential biomarkers for TS through a systematic meta-analysis designed to address specific questions or hypotheses (164). Our review indicates the potential usefulness of peripheral biomarkers in this context; however, further investigation is required to ascertain whether the statistical significance of our findings translates into diagnostic applicability.

We also note several limitations. Biomarkers discussed here are likely to be not specific, given that they are based on the evidence of association with other neurological or psychiatric disorders. Notably, we did not account for potential variations in assay sensitivity, methods of sample collection, and molecular detection employed in different studies. Furthermore, confounding factors such as diet, stress status, and the specific diagnostic criteria for TS were not controlled in our analysis. Additionally, the prior treatment experiences of patients could have influenced the differentiation between TS and control subjects, despite biomarker assessments being conducted during a treatment-free period. Moreover, the use of different cutoff positivity values in various studies may have introduced bias into our findings. In light of these limitations, our study indicates that the biomarkers under review hold promise for potential clinical applications. Nevertheless, it is crucial to conduct further investigations before considering their adoption in clinical practice. The deep integration of omics sciences could be taken into account.

## Conclusion

Our study demonstrated that TS is associated with peripheral levels of some T cells, NK-cells, anti-*Streptococcus* antibody positivity, Glu, Asp, Fe, Pb, Zn, vitamin D, and BDNF. Further studies should be conducted to replicate these findings before they are used in clinical settings.

## Data availability statement

The original contributions presented in the study are included in the article/Supplementary material, further inquiries can be directed to the corresponding author.

## Author contributions

YJ: Conceptualization, Data curation, Methodology, Writing – original draft. YL: Visualization, Writing – original draft. XC: Data curation, Writing – original draft. RZ: Data curation, Writing – review & editing. YP: Visualization, Writing – review & editing. RT: Data curation, Writing – review & editing. CZ: Data curation, Writing – review & editing. JW: Conceptualization, Writing – review & editing.

## Footnotes

^1^https://smcgrath.shinyapps.io/estmeansd/

^2^https://www.statstodo.com/CombineMeansSDs.php
