# Supplementary material for: Biomarkers and Tourette syndrome: a systematic review and meta-analysis
Source: Front Neurol. 2024 Feb 7;15:1262057. doi: 10.3389/fneur.2024.1262057 (PMC10879287; doi:10.3389/fneur.2024.1262057)

Figure S1 Forest plots for standard mean difference (SMD) from meta-analysis of serum CD8+T cell (a) and CD19+ lymphocyte (b) levels.

(a)


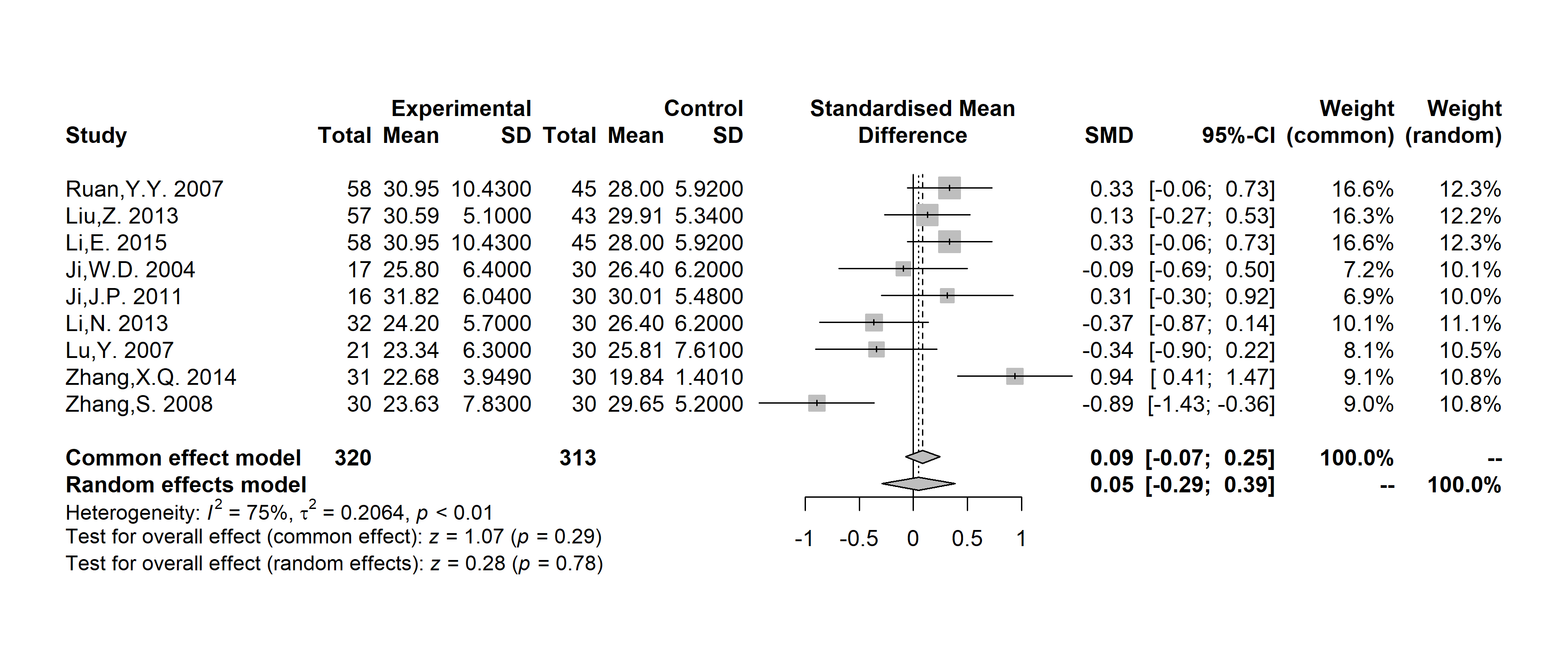


（b）


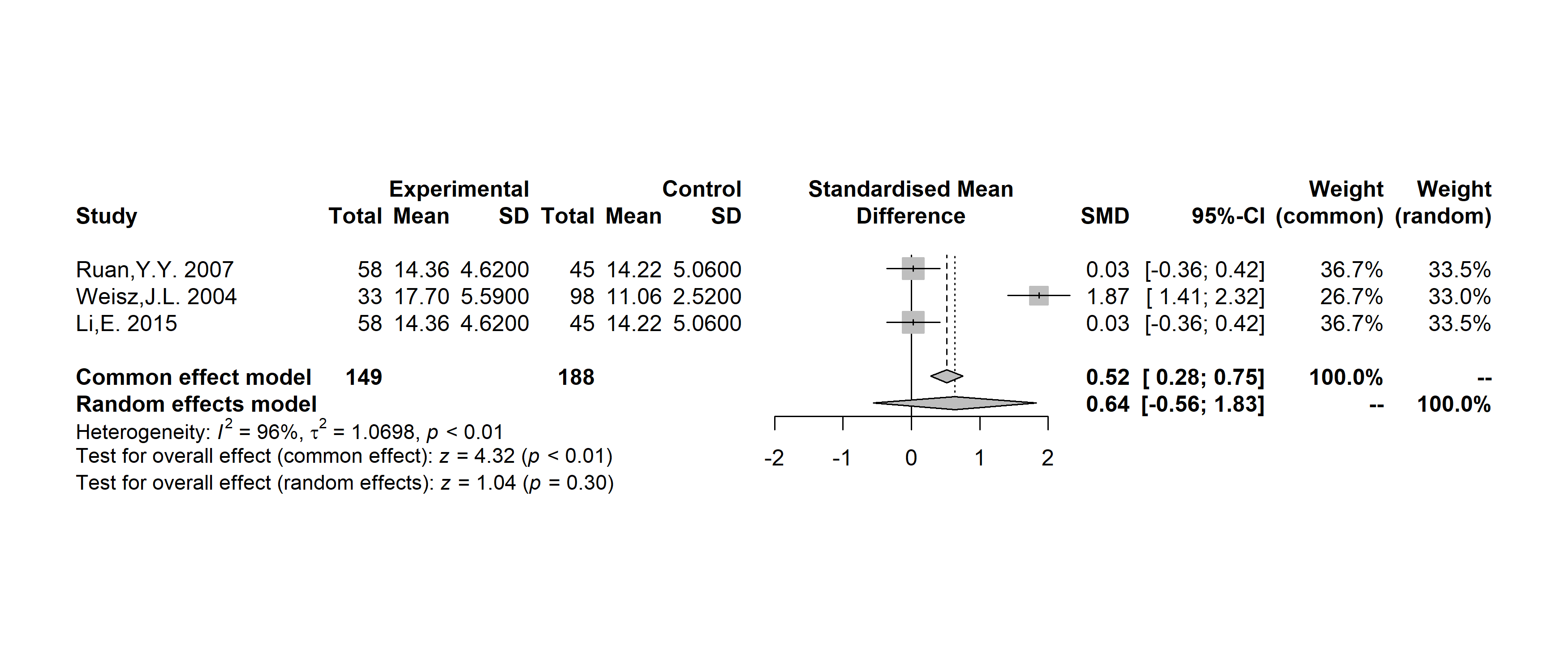


Figure S2 Forest plots for standard mean difference (SMD) from meta-analysis of serum immunoglobulin (Ig) levels (IgA (a); IgM (b); IgG (c)).

(a)


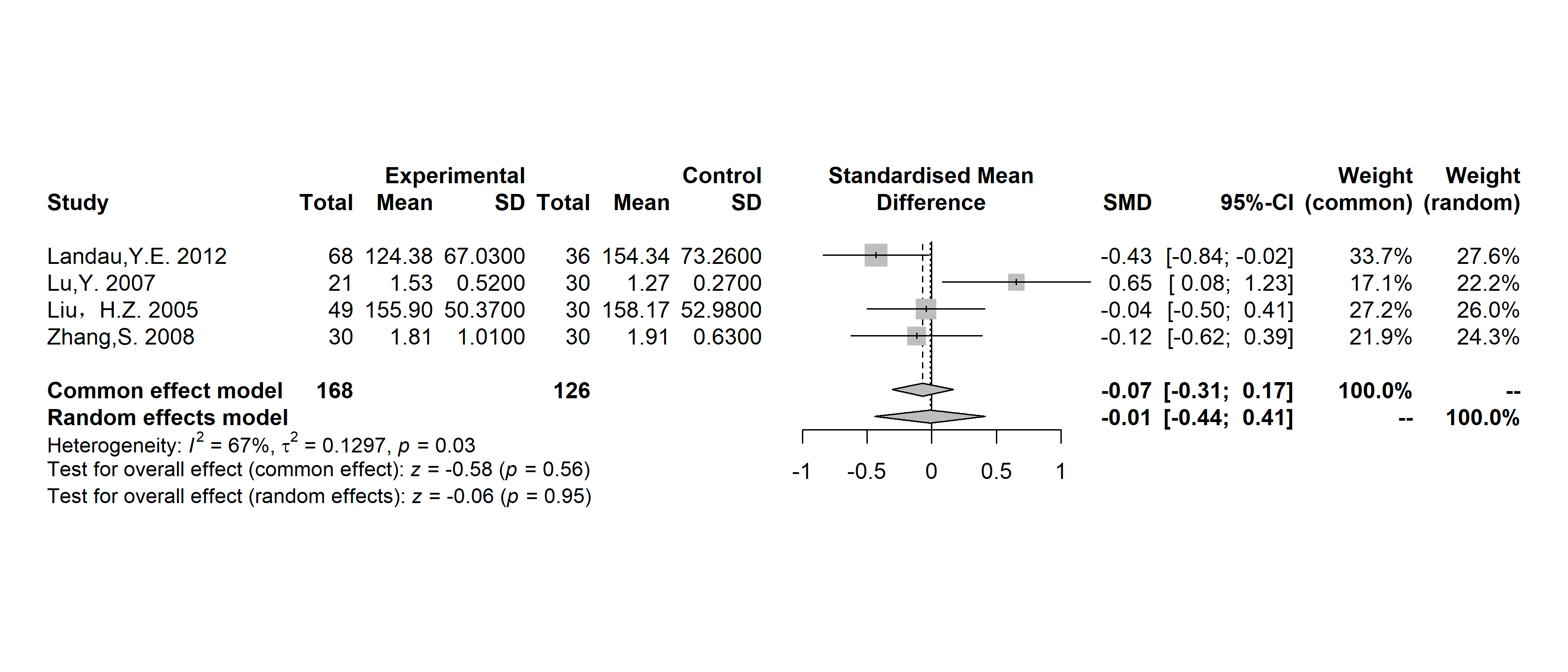


(b)


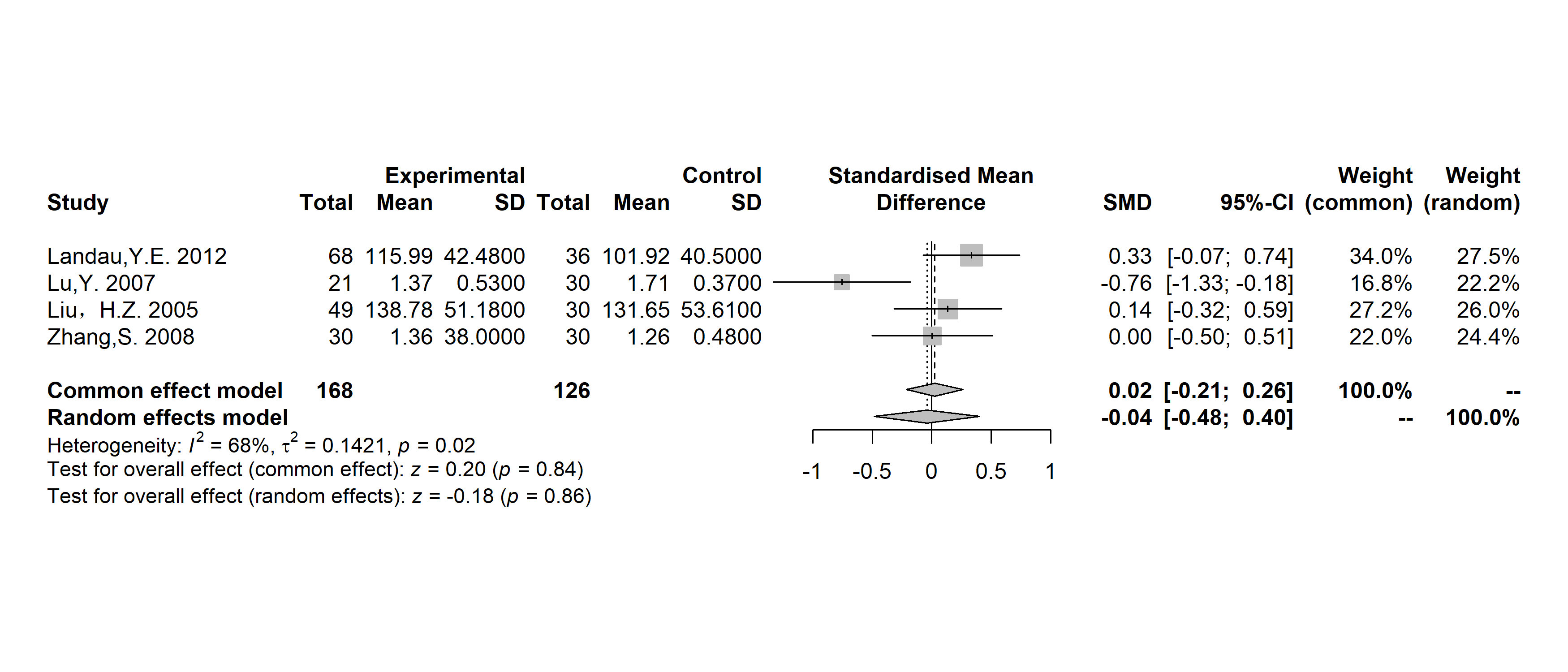


(c)


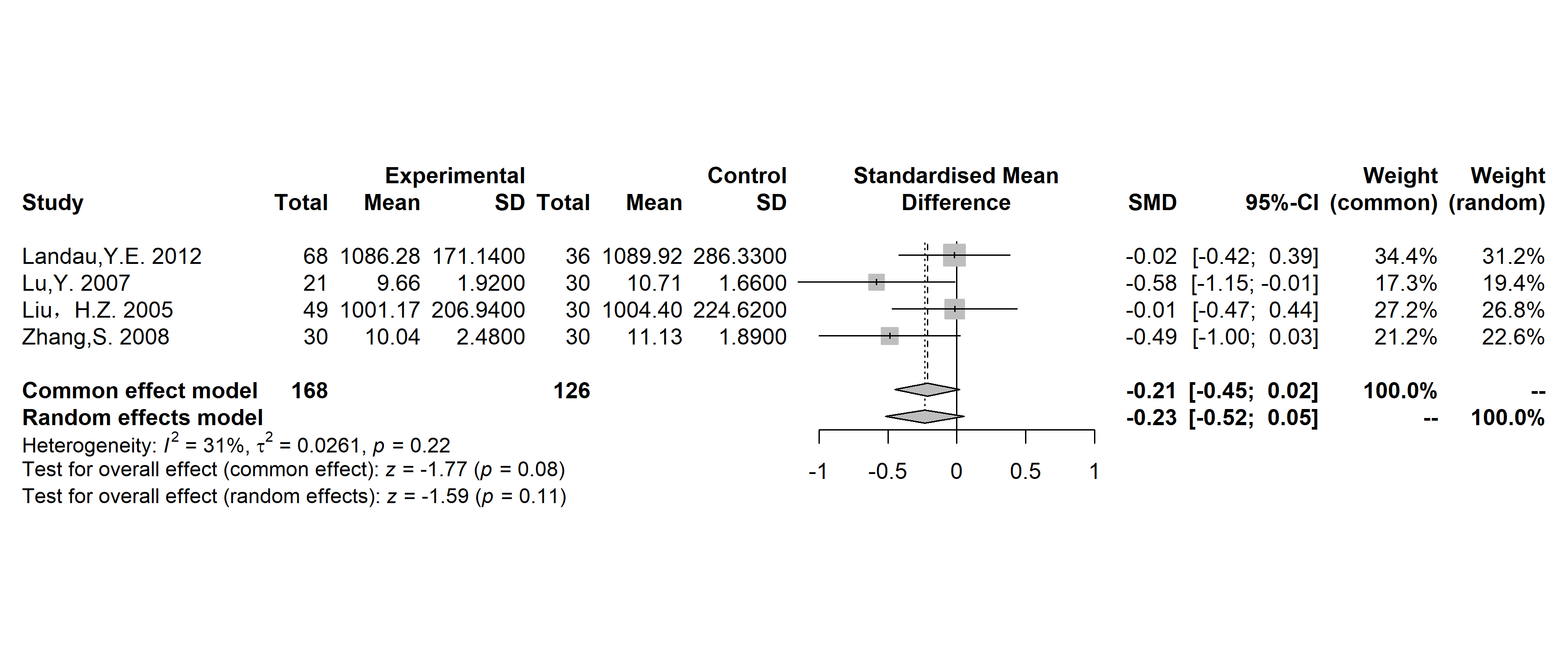


Figure S3 Forest plots for standard mean difference (SMD) from meta-analysis of serum C3 (a) and C4 (b) levels.

(a)


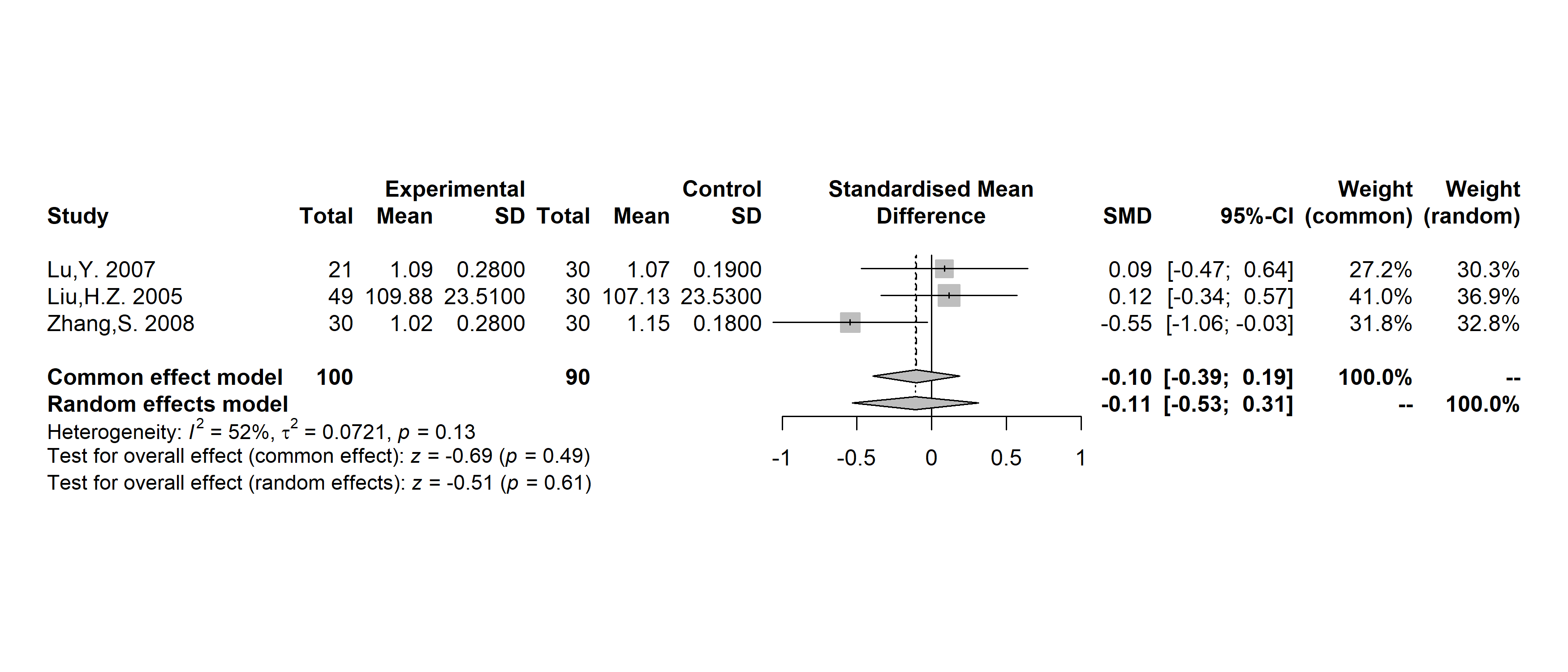


(b)


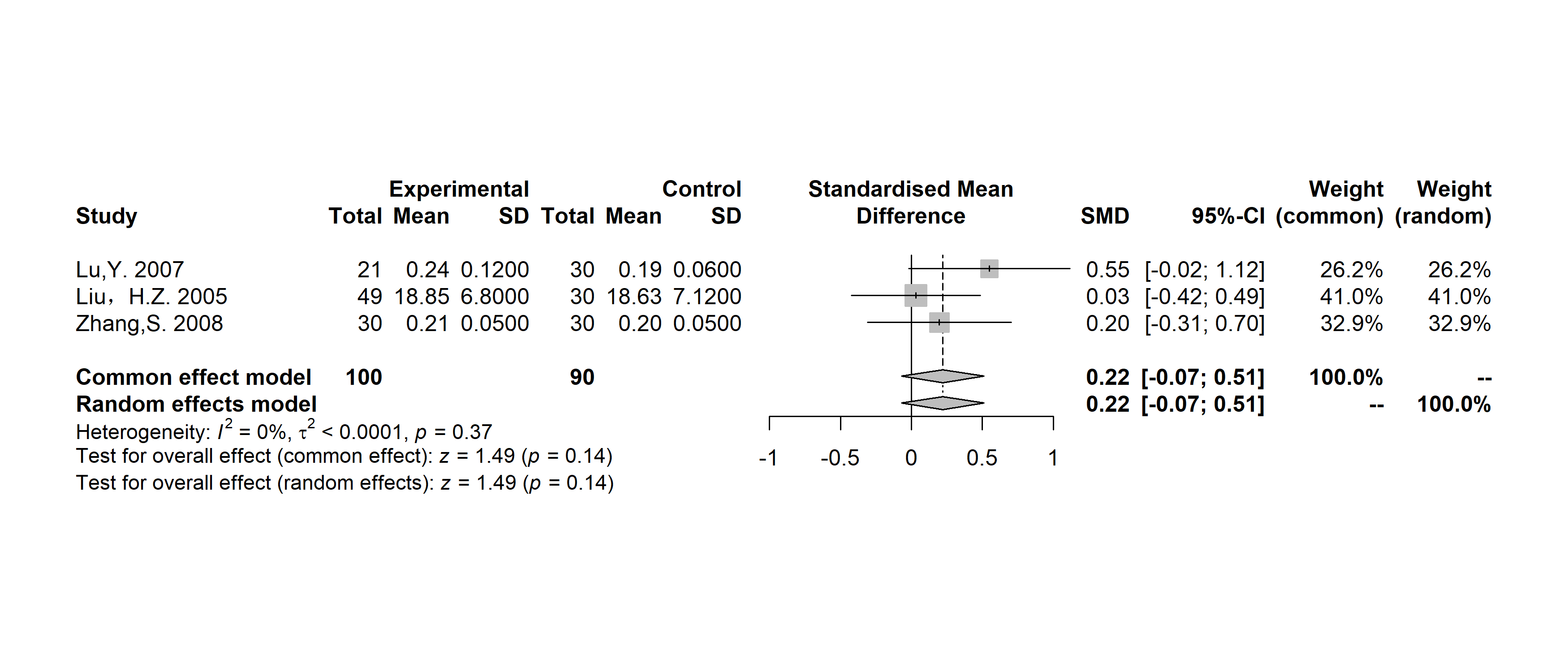


Figure S4 Forest plots for standard mean difference (SMD) from meta-analysis of IFN-γ (a), IL-1β (b), IL-4 (c), IL-6 (d), IL-8 (e), IL-12 (f) and TNF-α (g) levels.

(a)


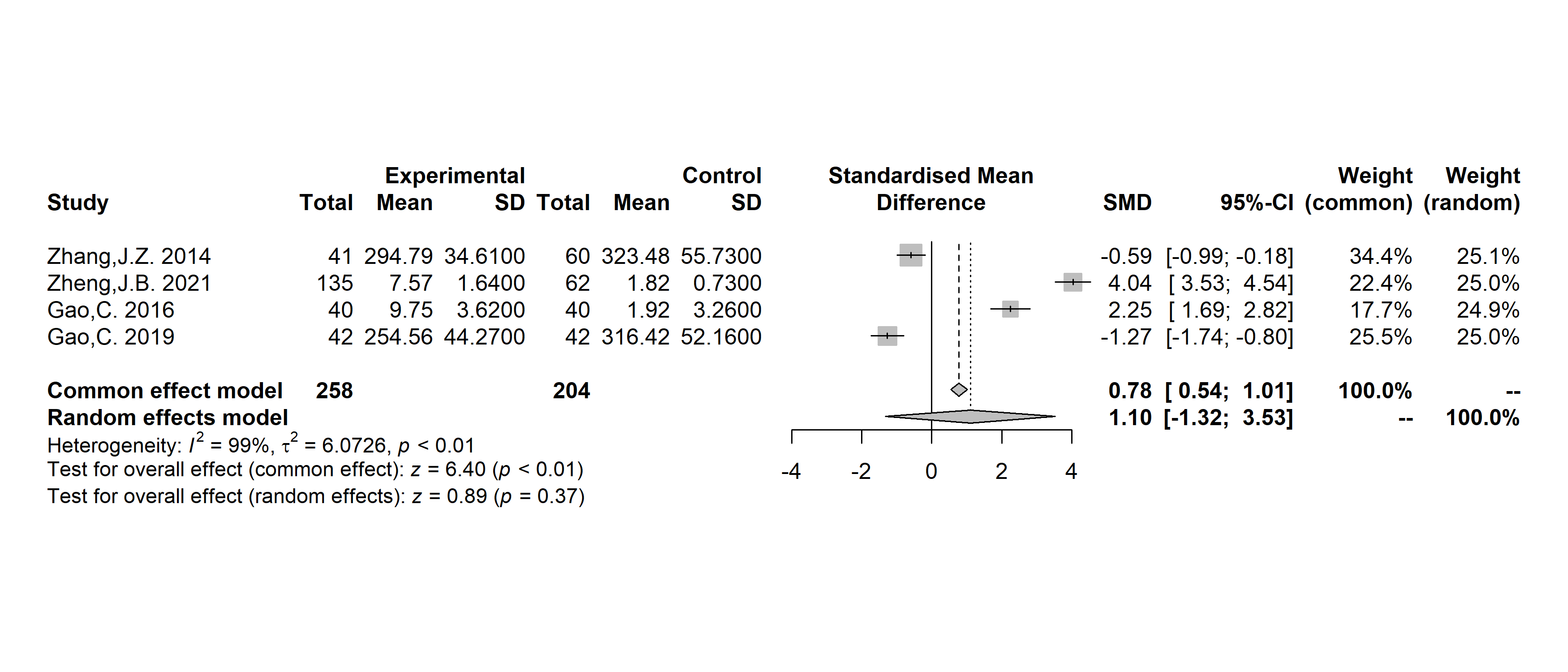


(b)


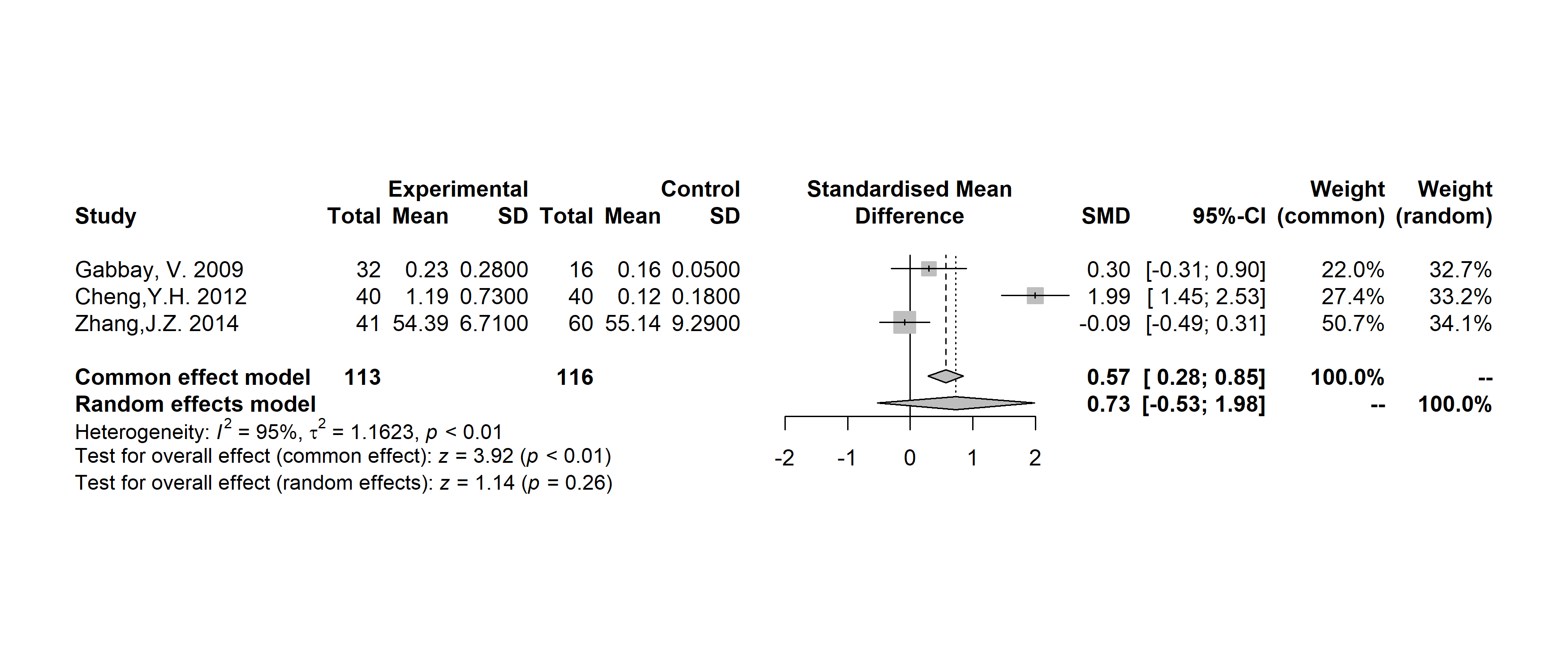


(c)


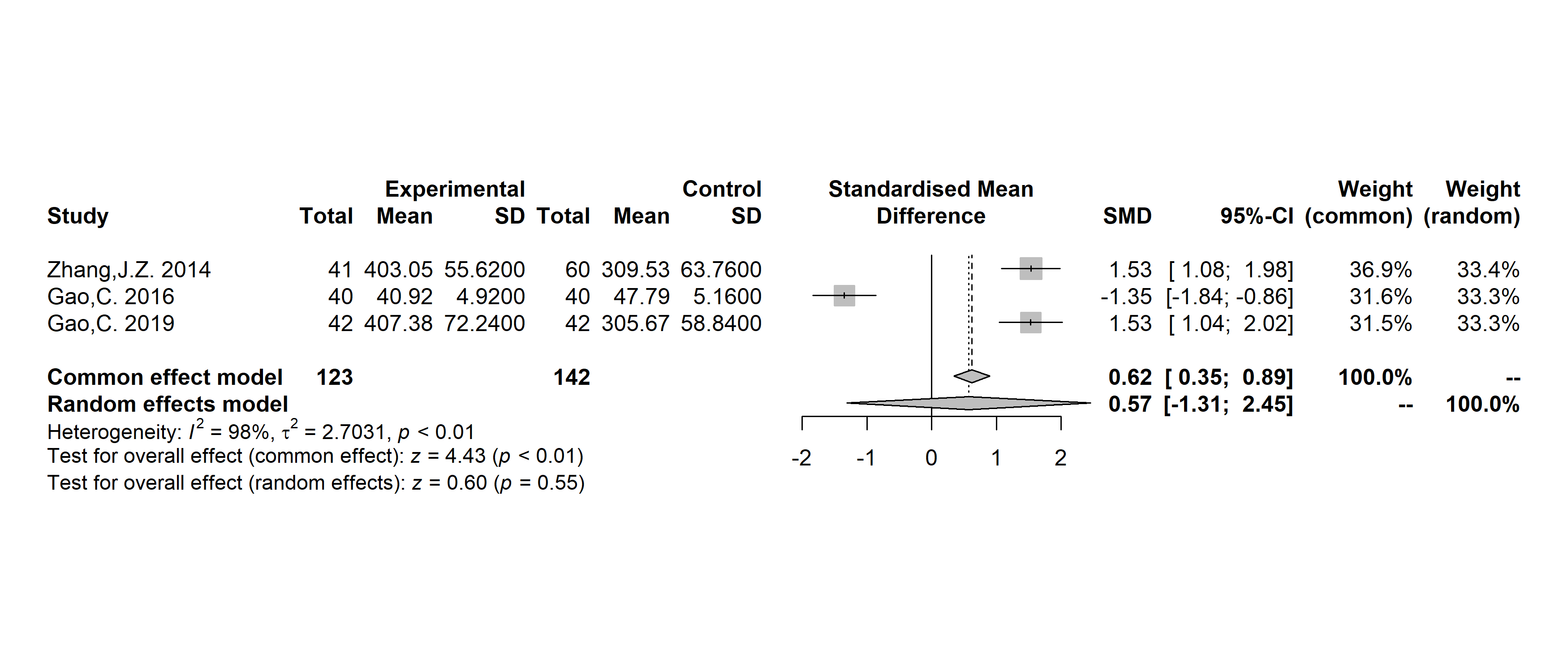


(d)


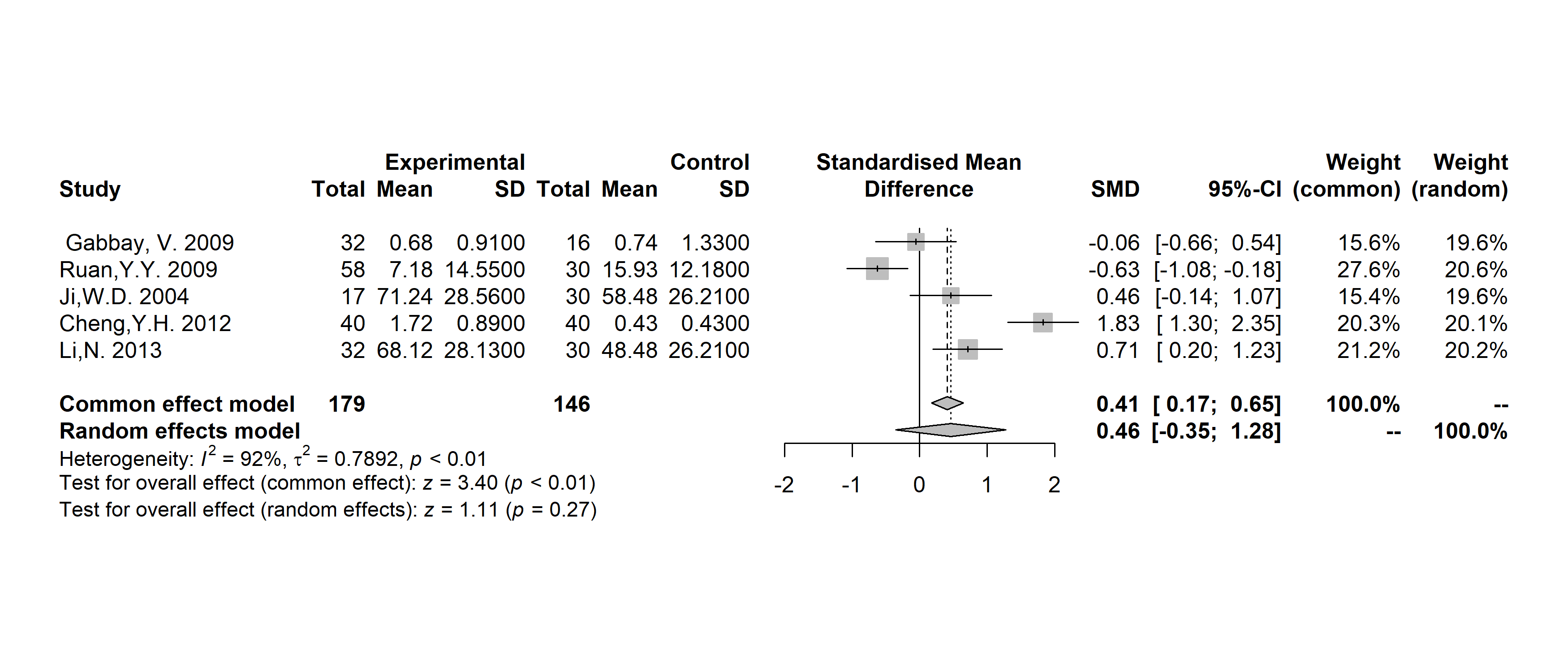


(e)


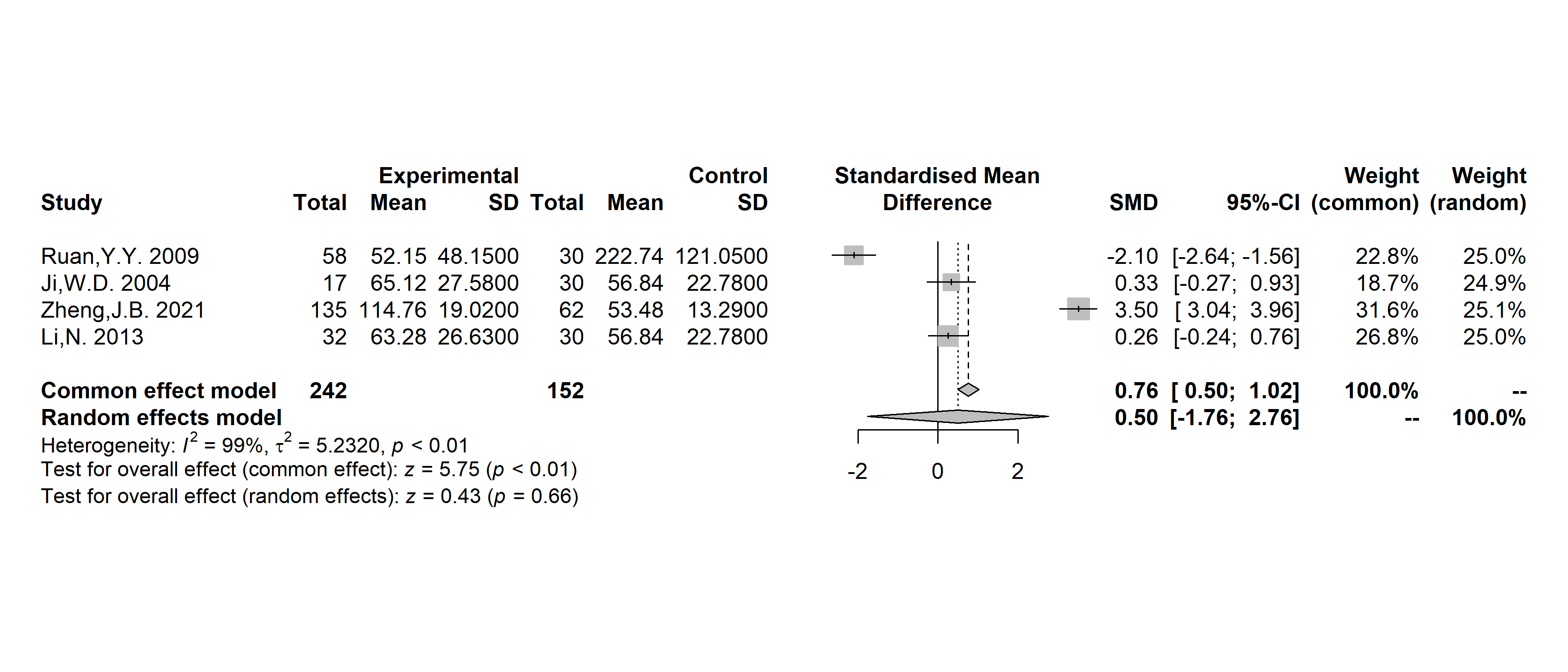


(f)


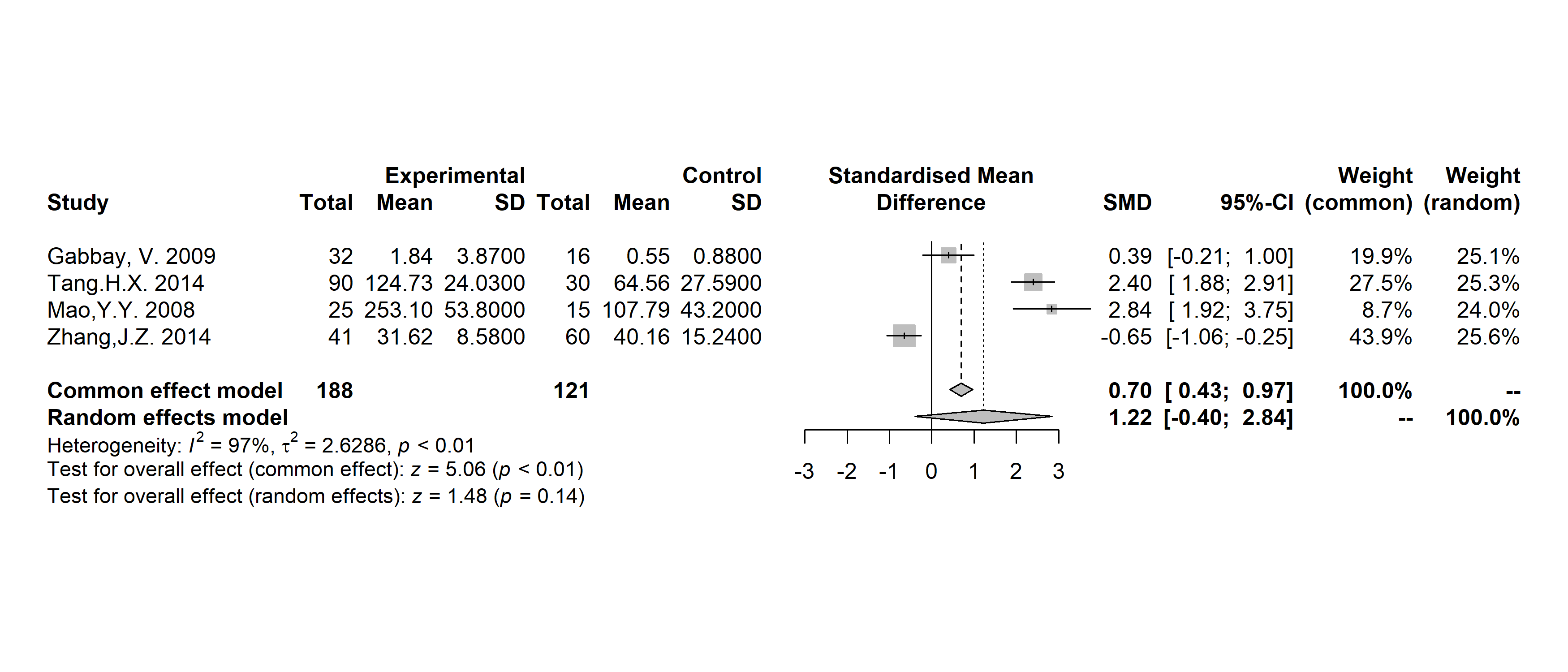


(g)


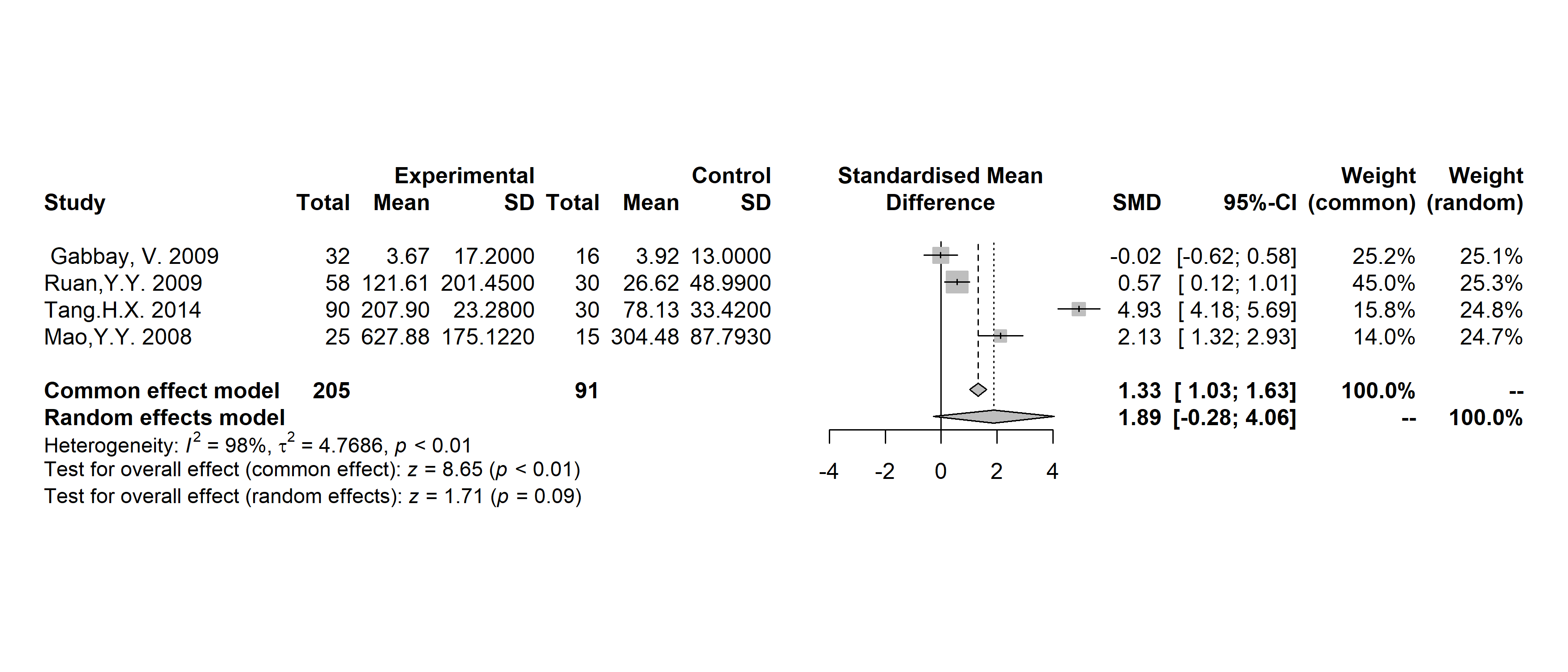


Figure S5 Forest plots for standard mean difference (SMD) from meta-analysis of monoamine neurotransmitters (5-HT (a); DA (b); NE (c)) and GABA (d) levels.

(a)


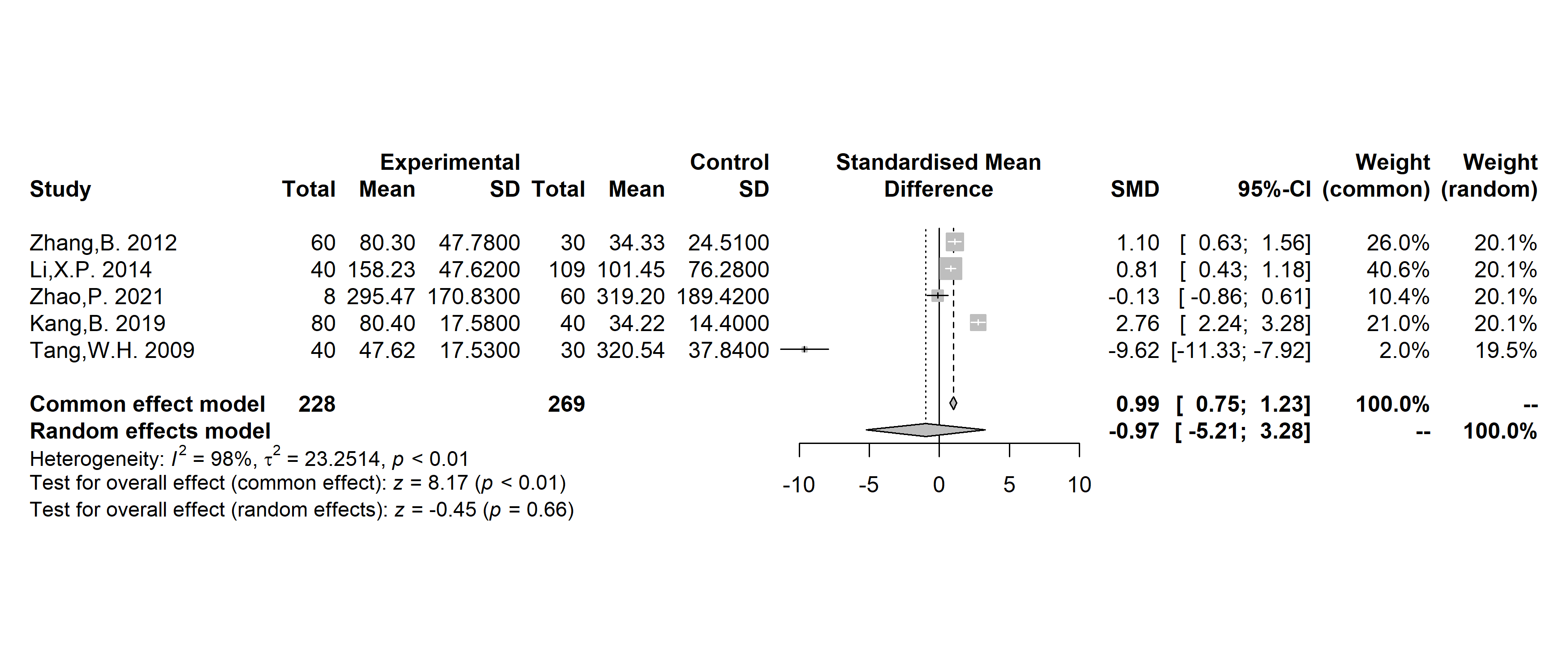


(b)


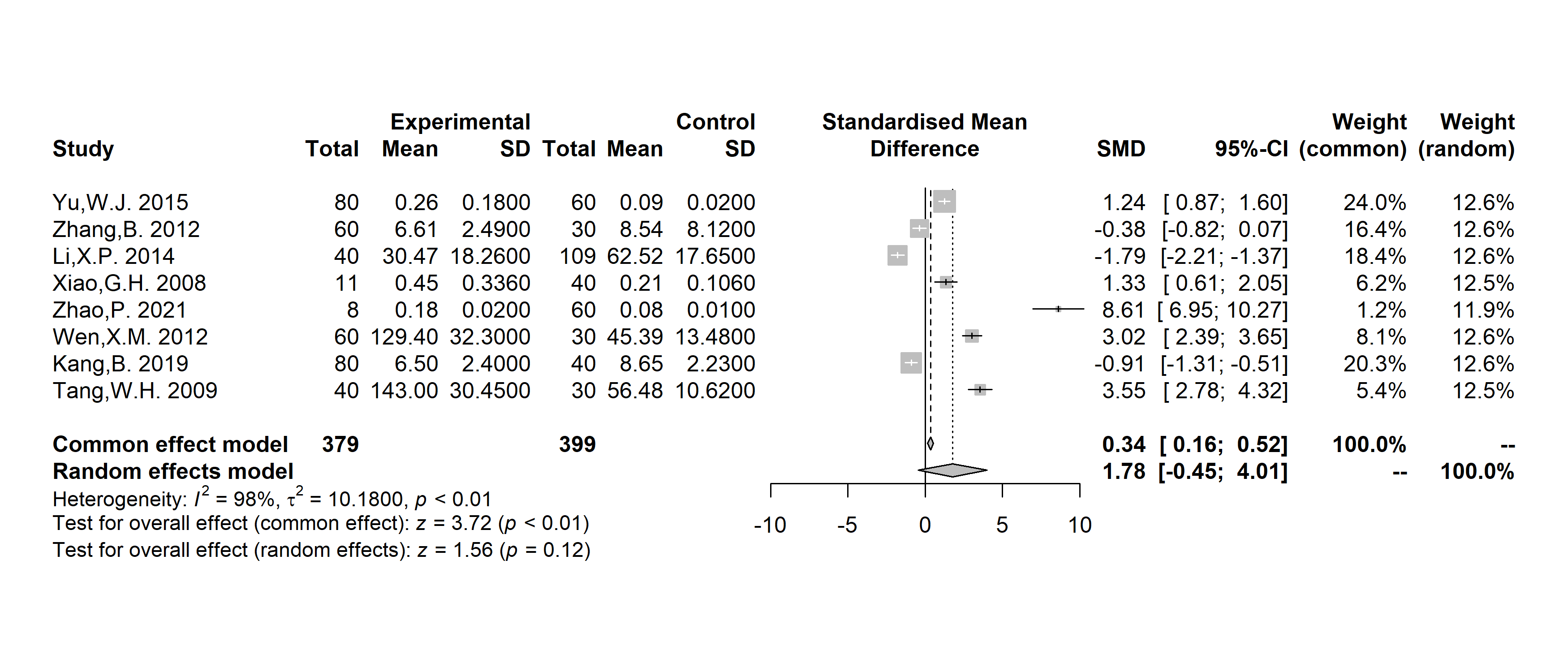


(c)


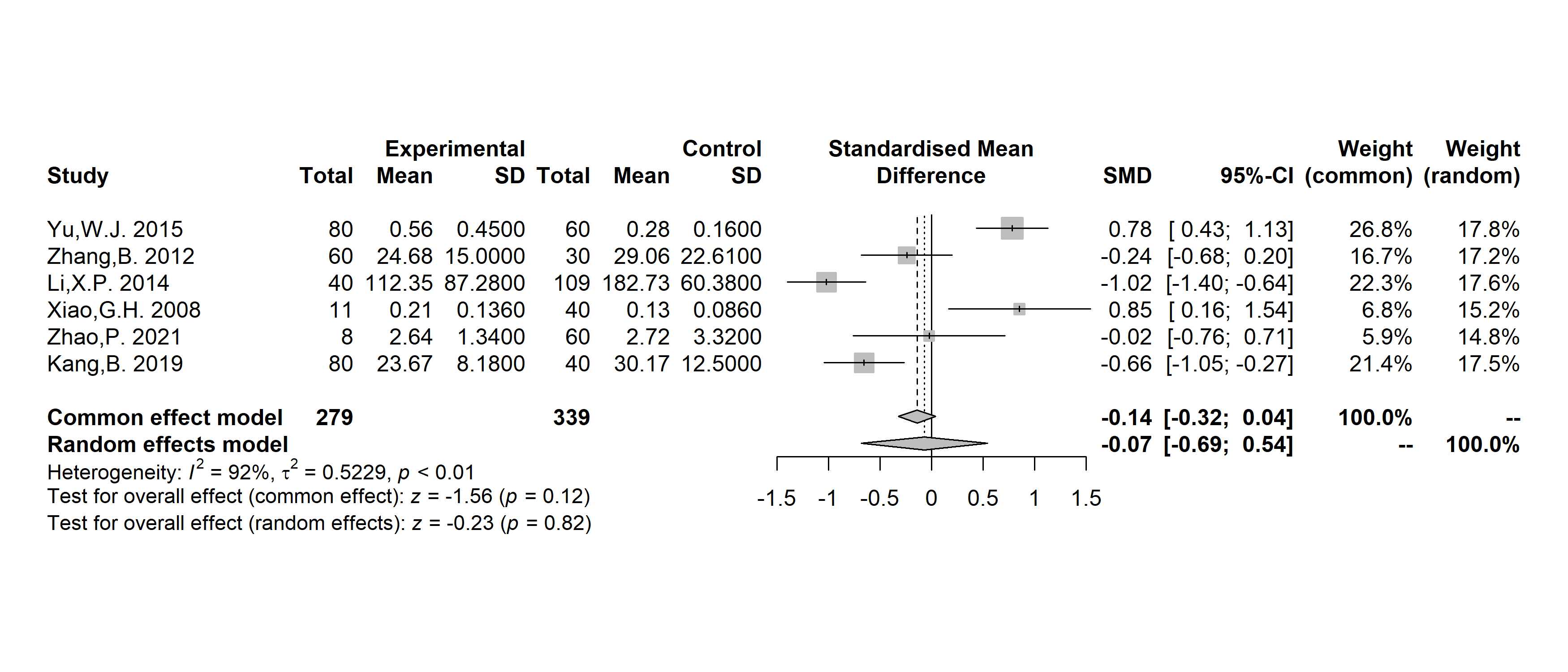


(d)


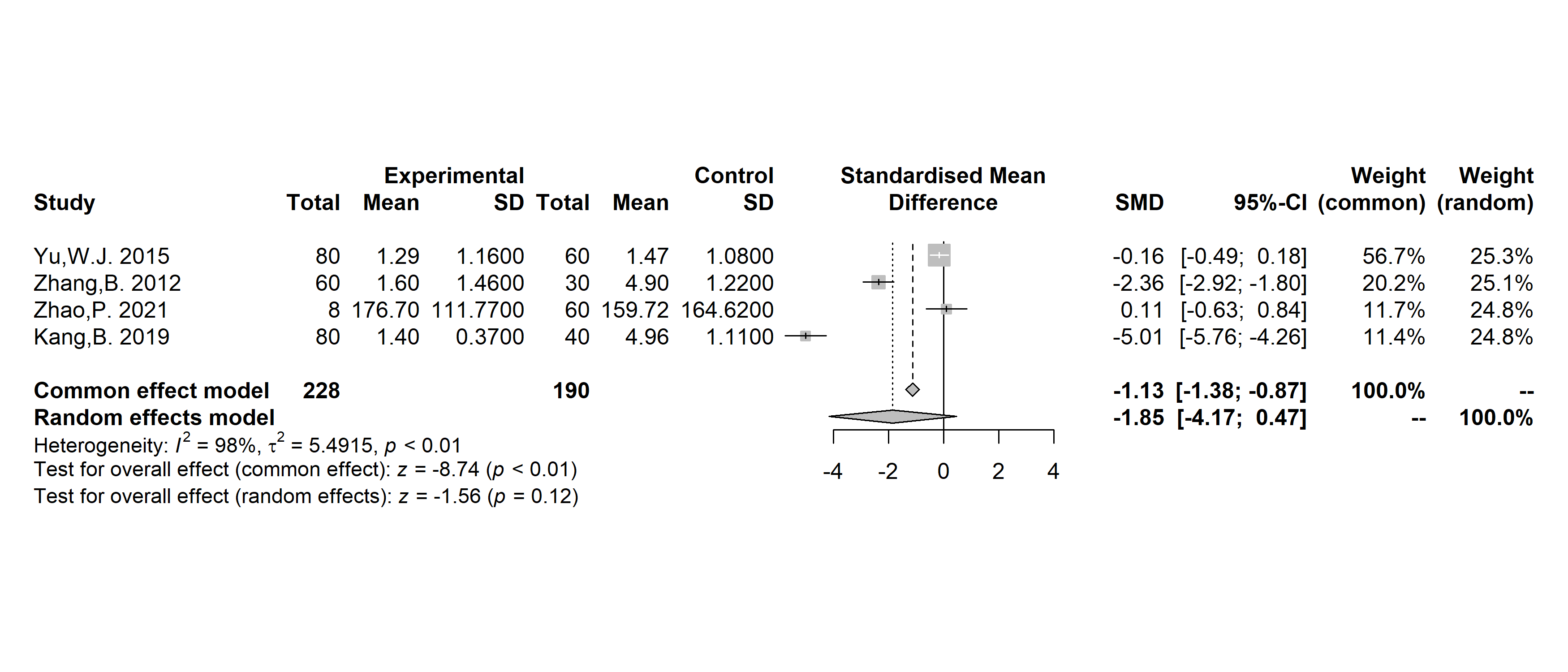


Figure S6 Forest plots for standard mean difference (SMD) from meta-analysis of blood Ca (a), Mg (b) and Cu (c) levels.

(a)


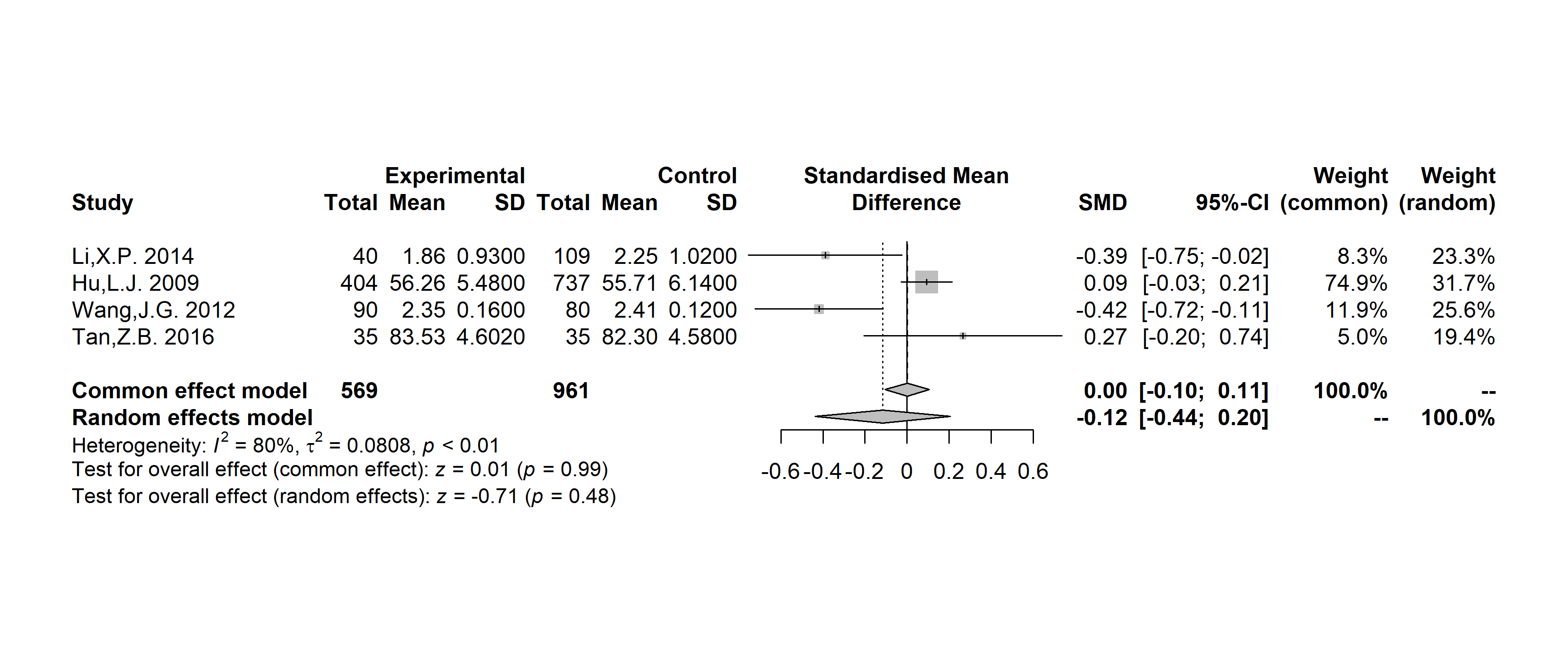


(b)


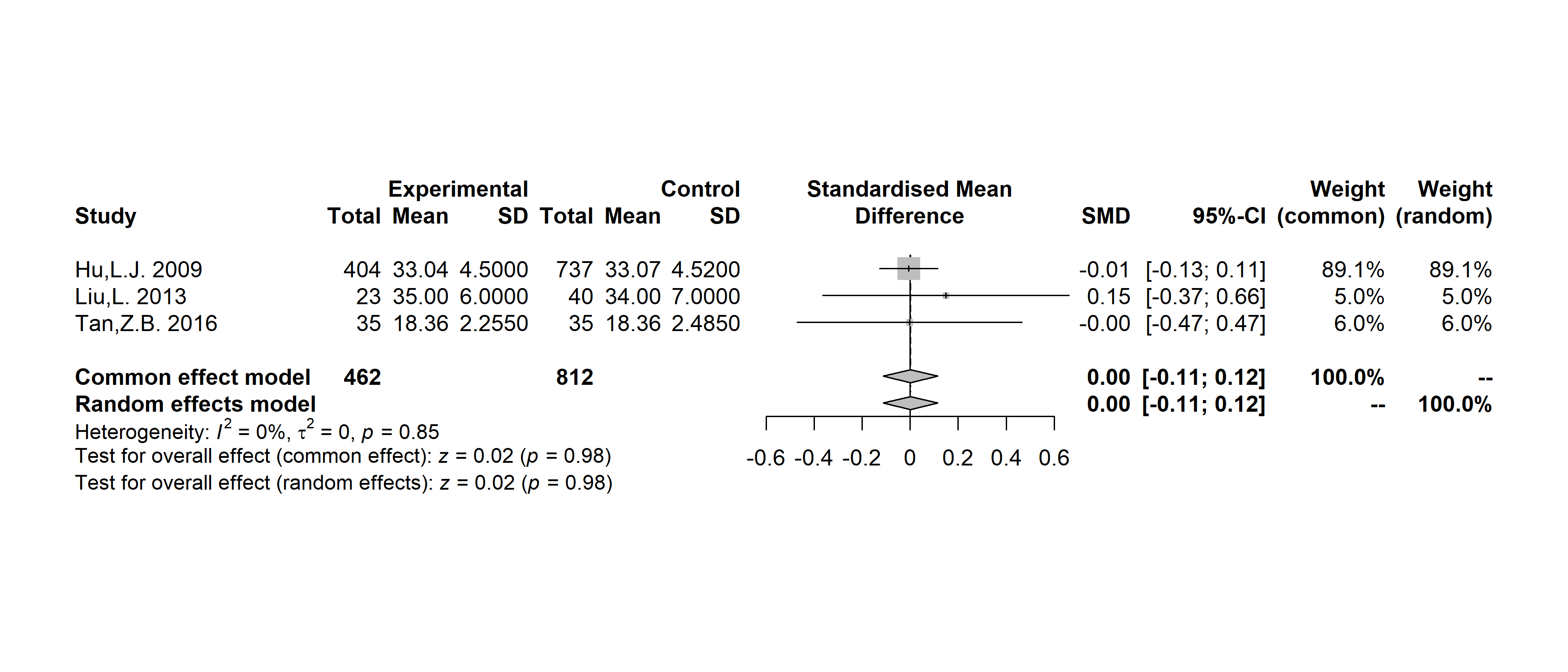


(c)


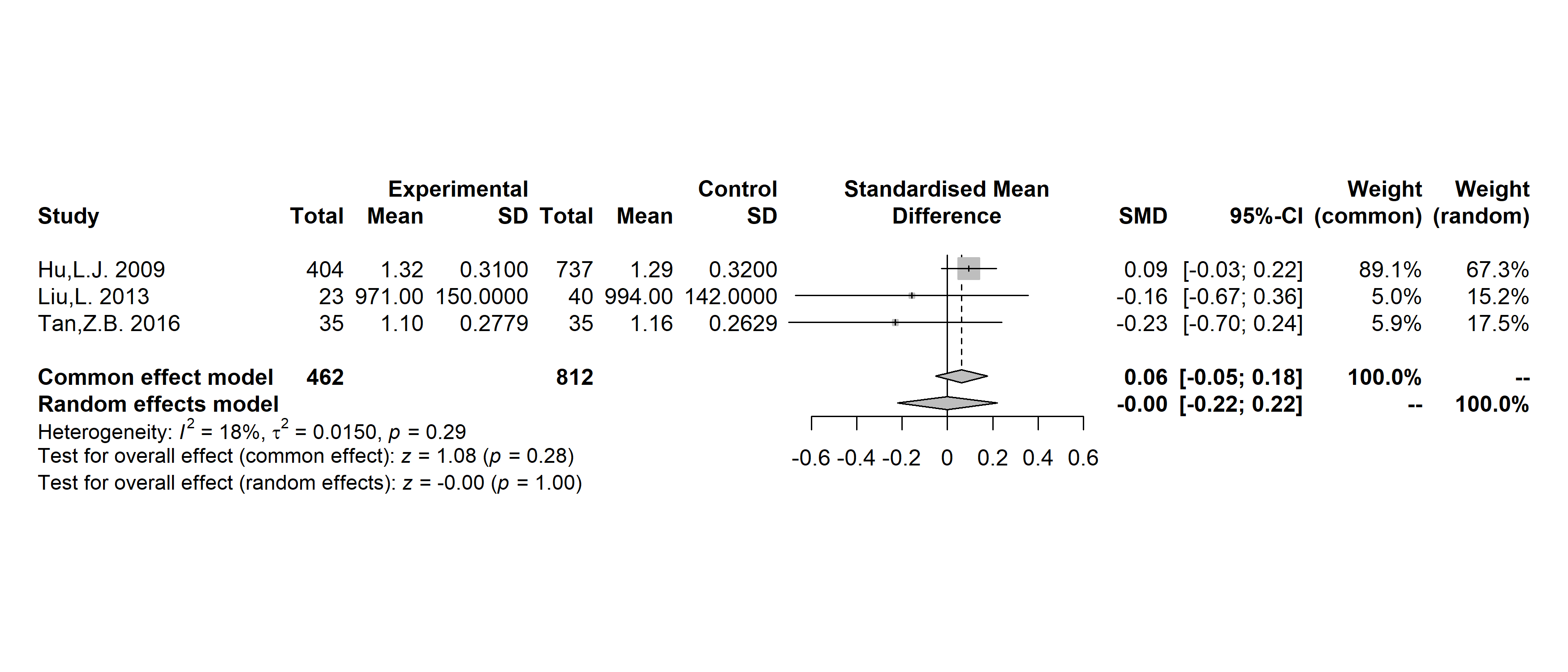


Figure S7 Forest plots for standard mean difference (SMD) from meta-analysis of blood cortisol (a) and PRL (b) levels.

(a)


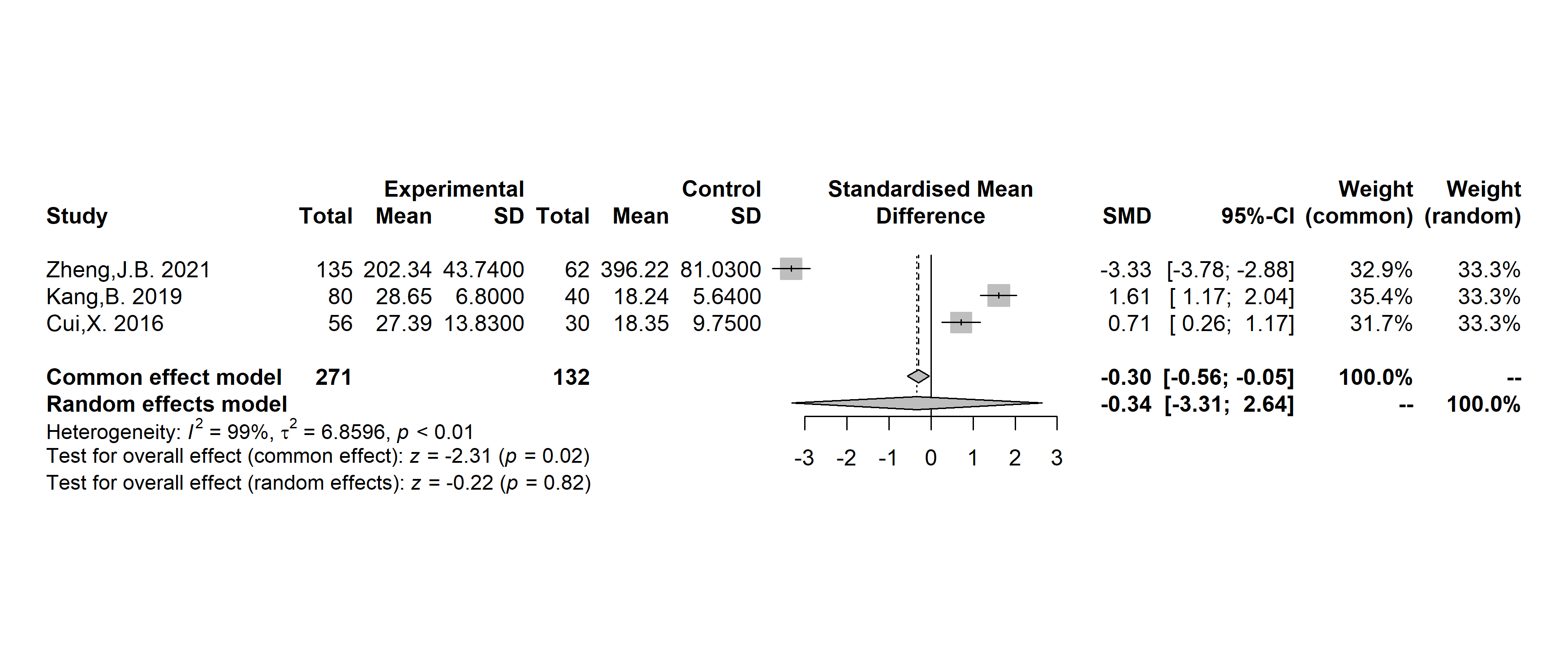


(b)


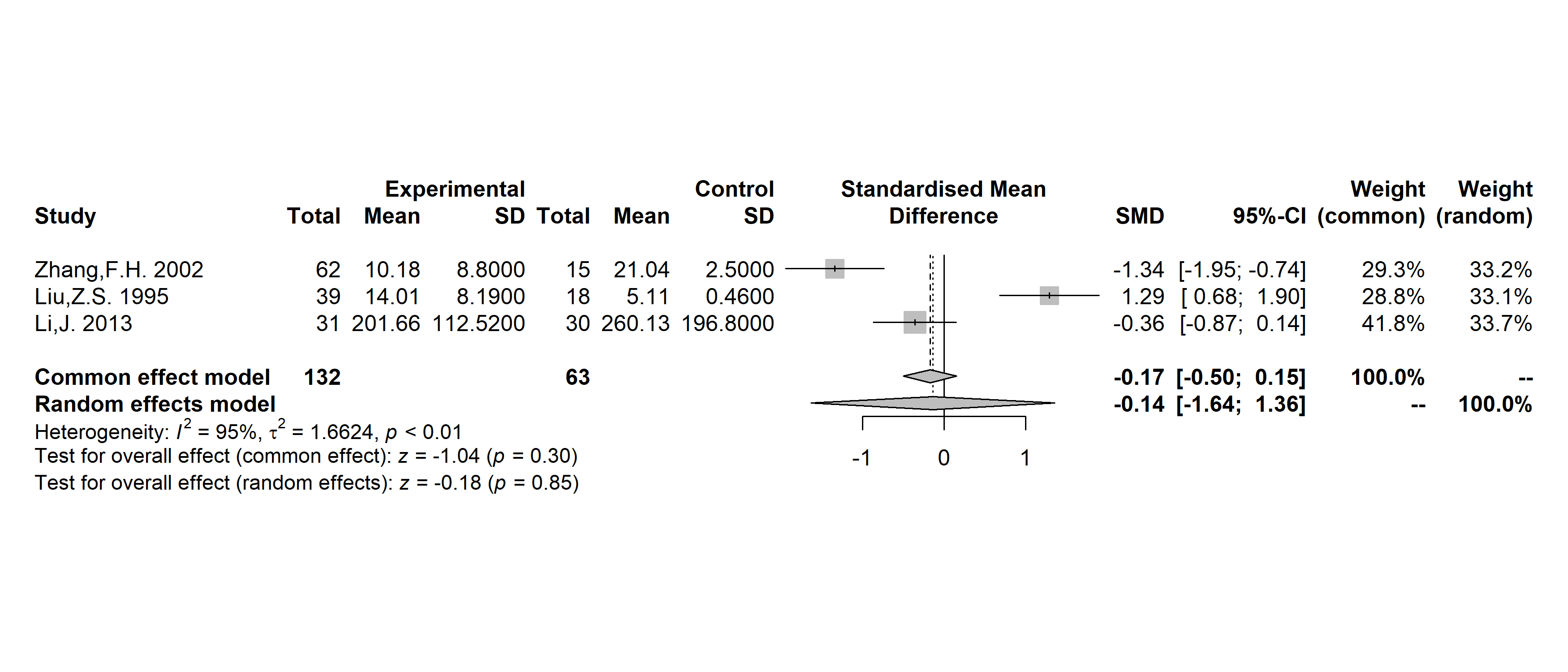


Figure S8 Funnel plots of serum CD3+T cell (a), CD4+T cell (b), CD4+T cell to serum CD8+T cell ratio (c) and anti-streptolysin O antibodies (d) in TS samples vs controls.

(a) (b)


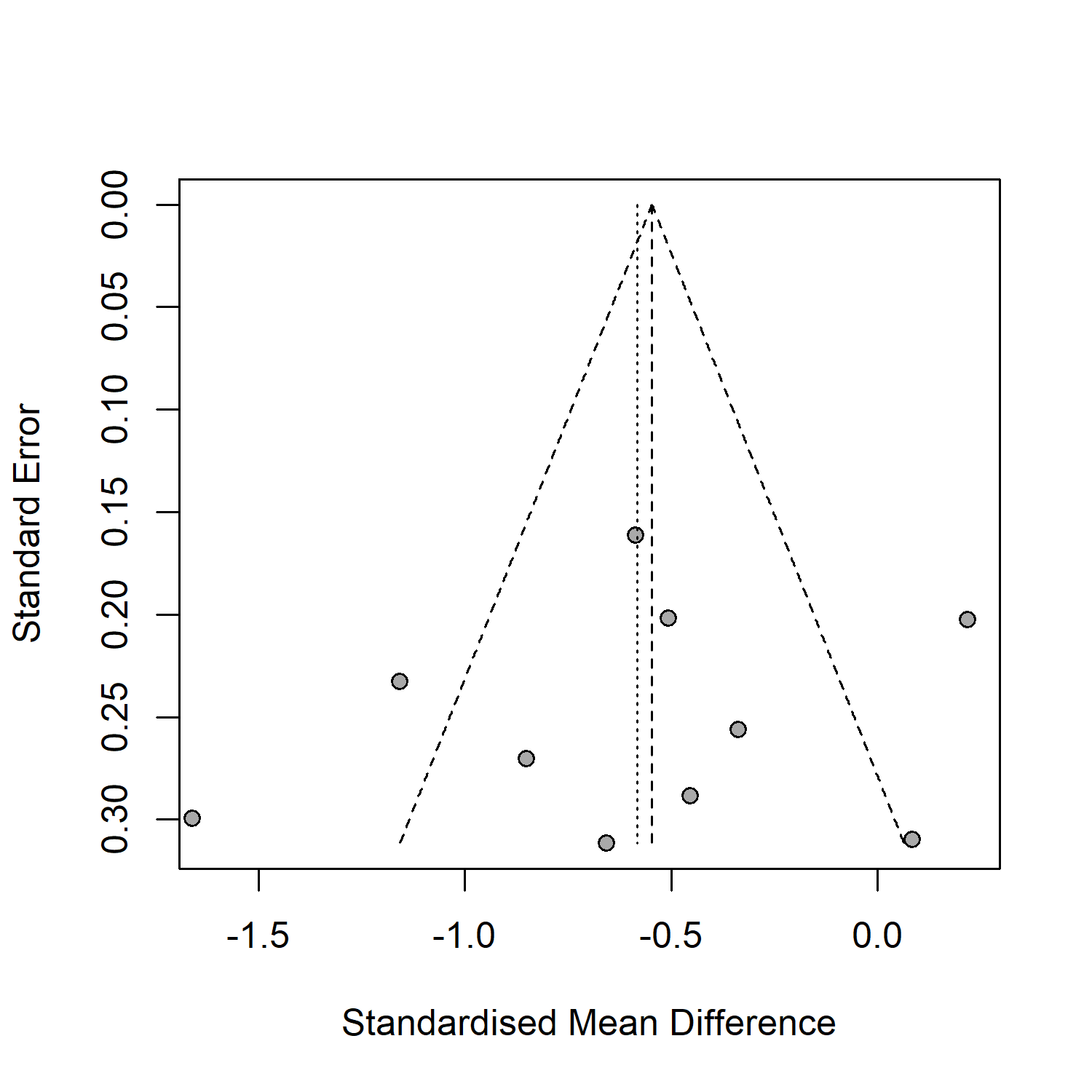

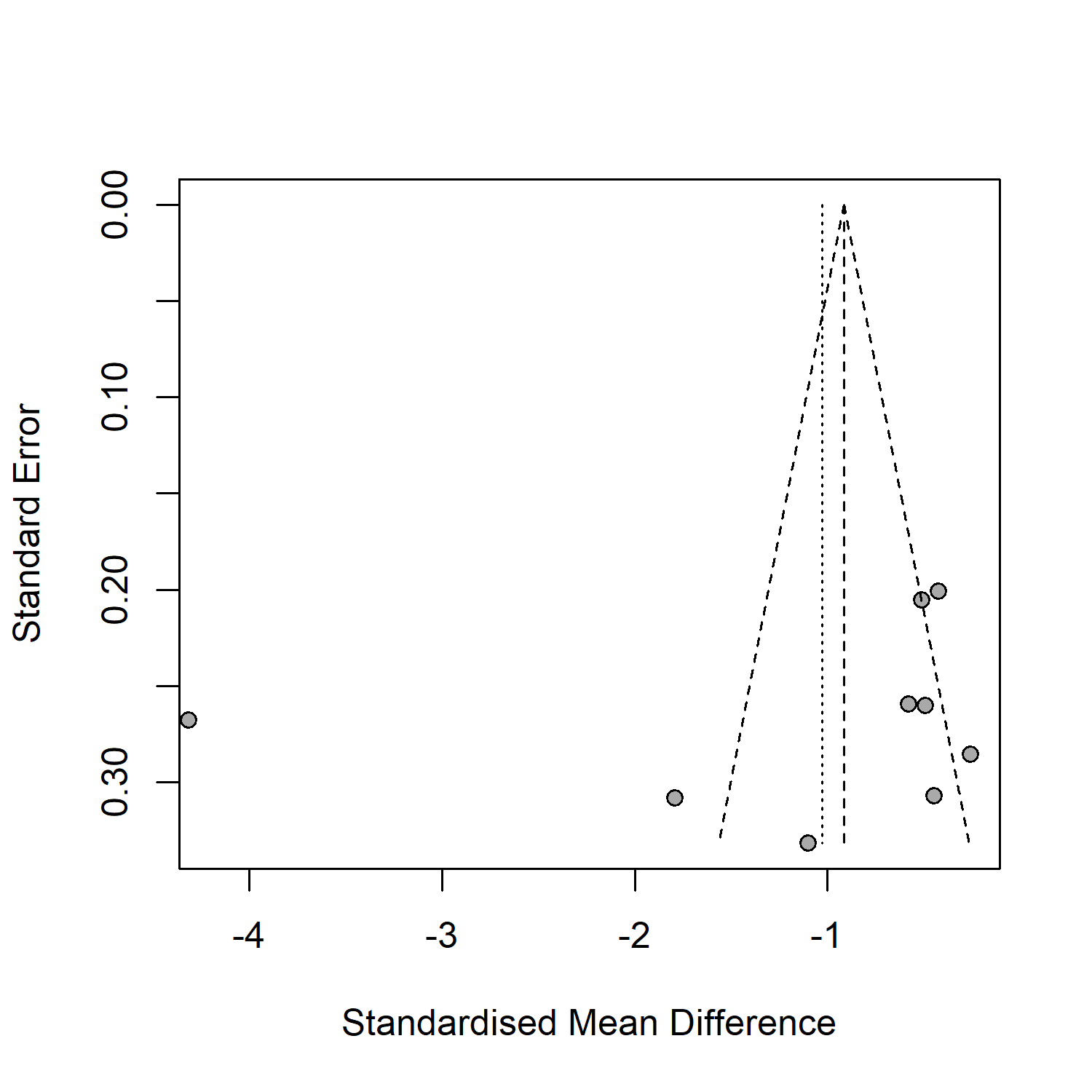


(c) (d)


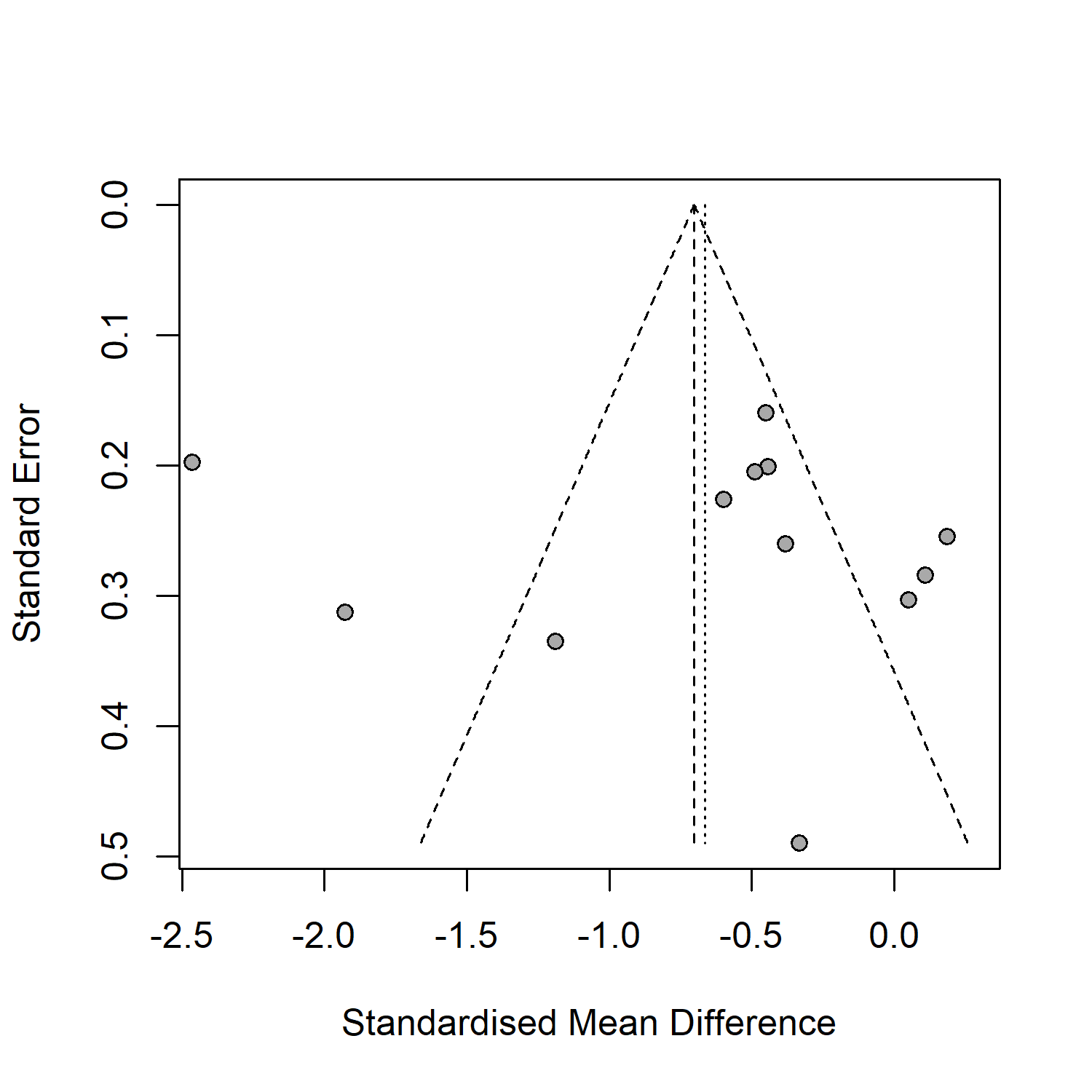

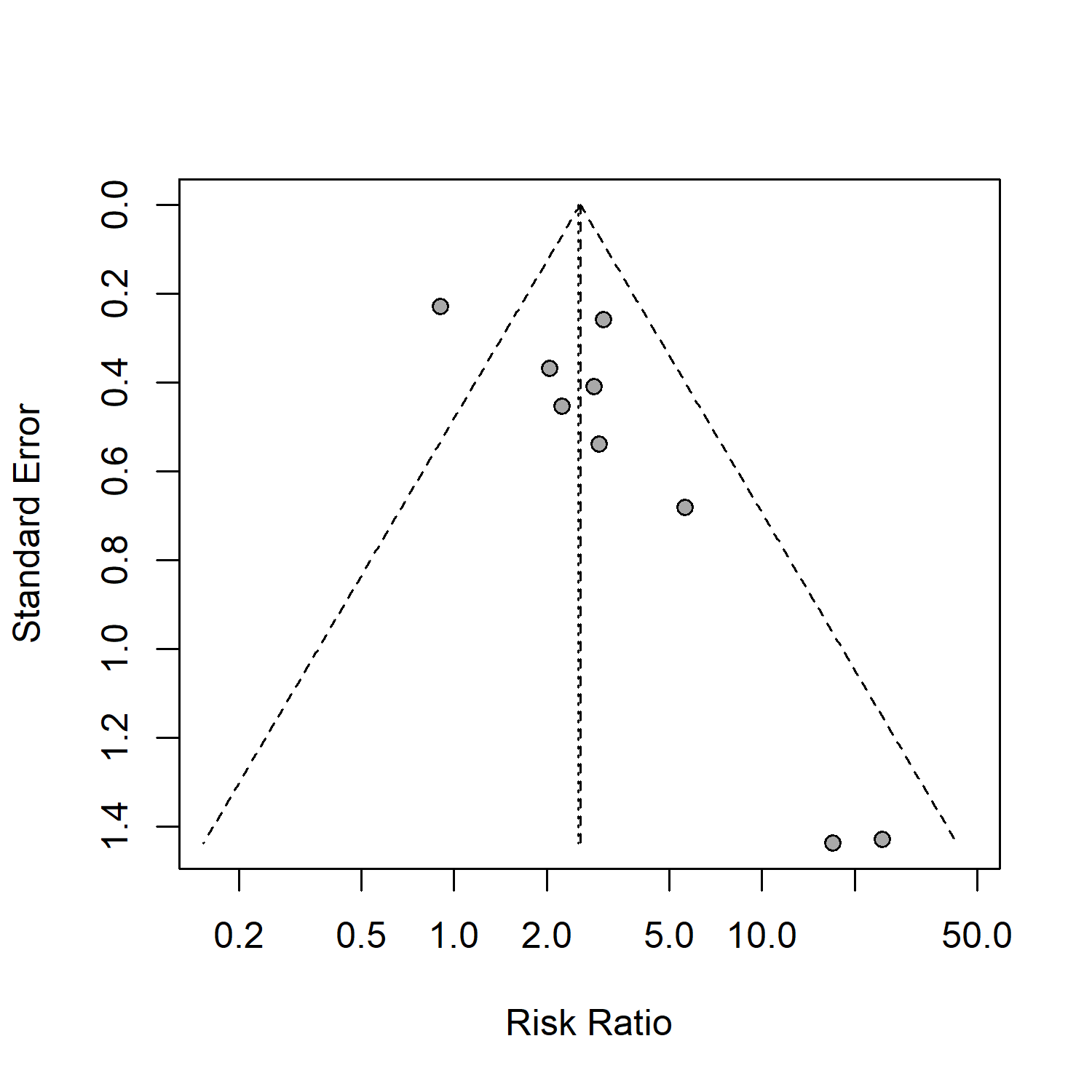


Figure S9 Sensitivity analysis of serum CD3+T cell (a), CD4+T cell (b), NK cell (c) and CD4+T cell to serum CD8+T cell ratio (d) in TS samples vs controls.

(a)


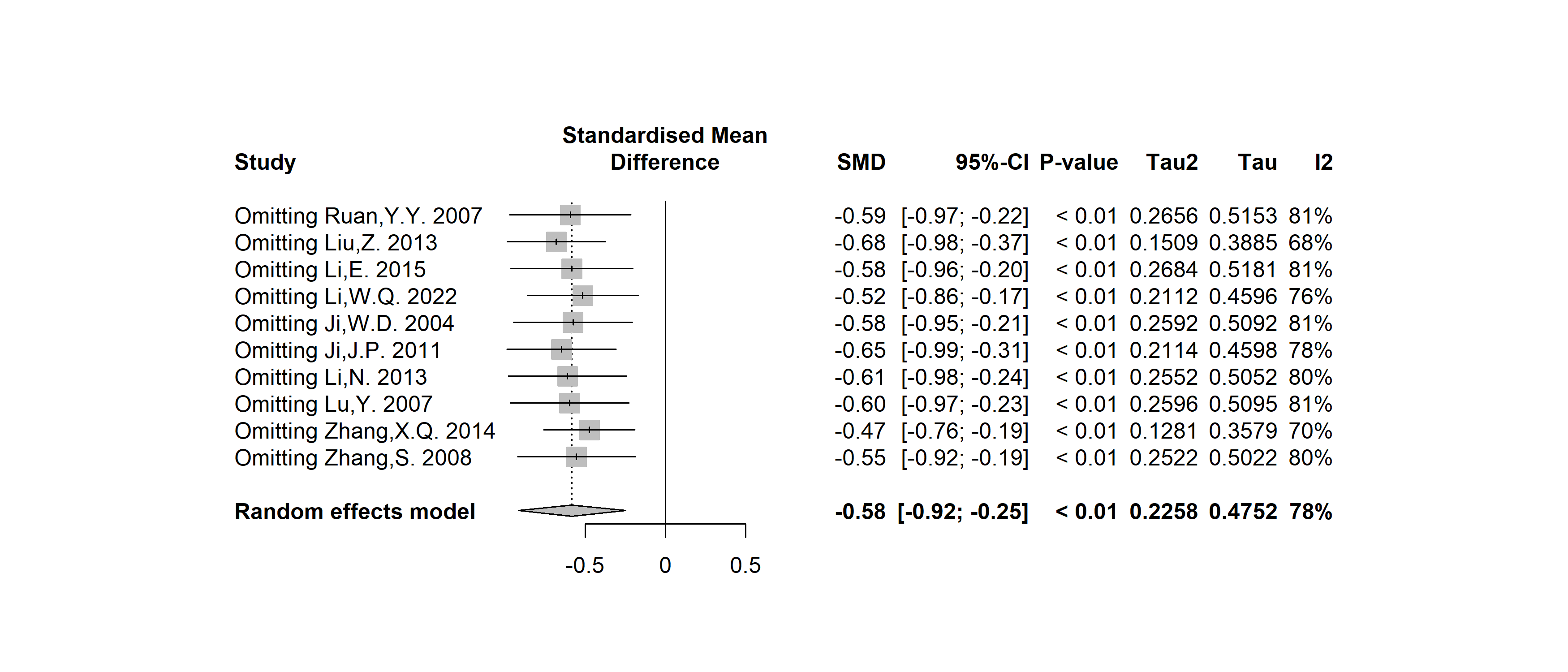


(b)


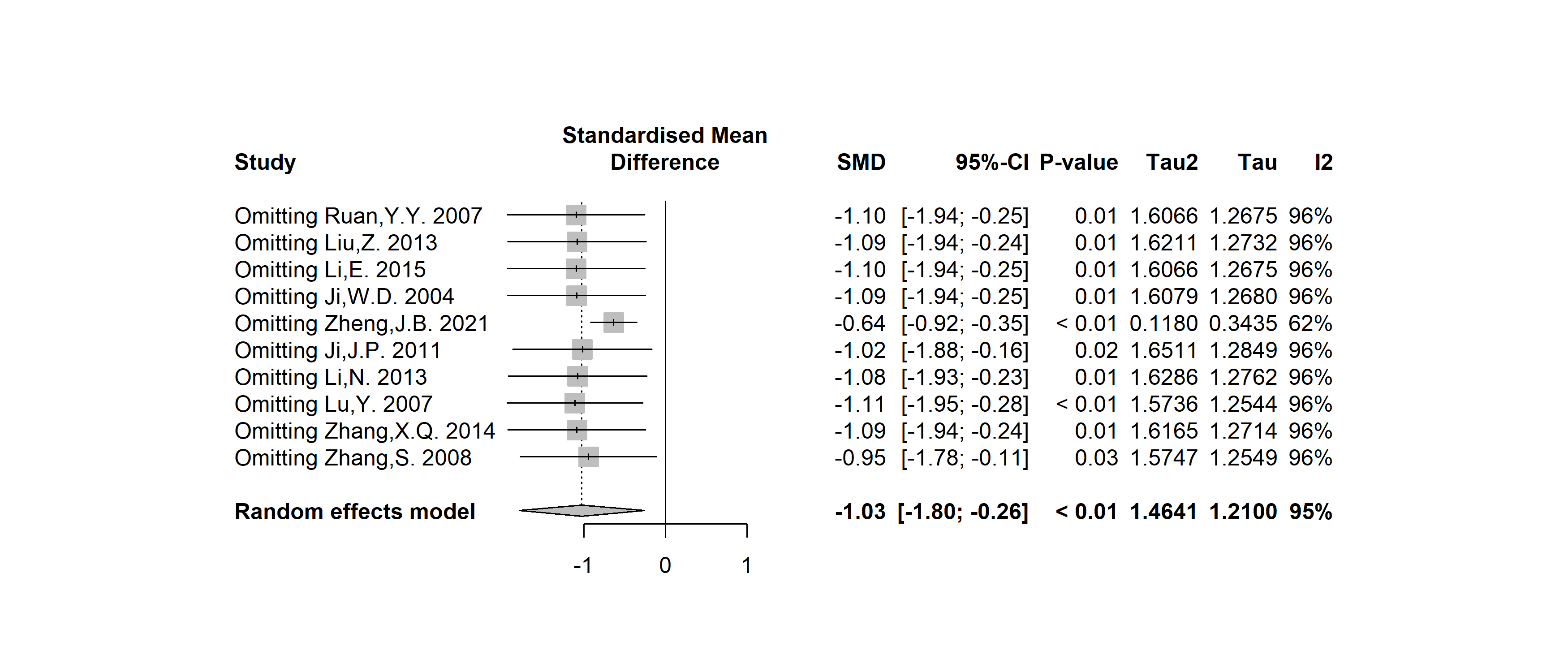


(c)


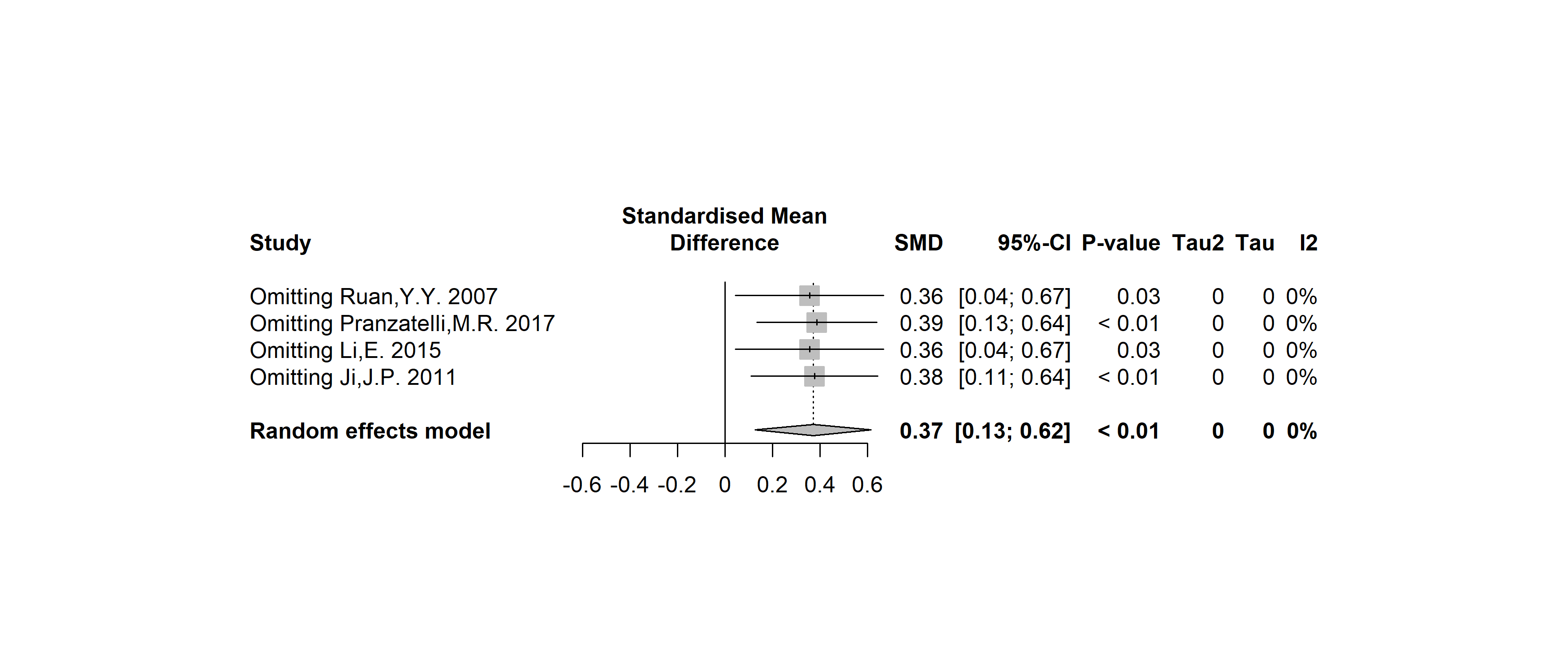


(d)


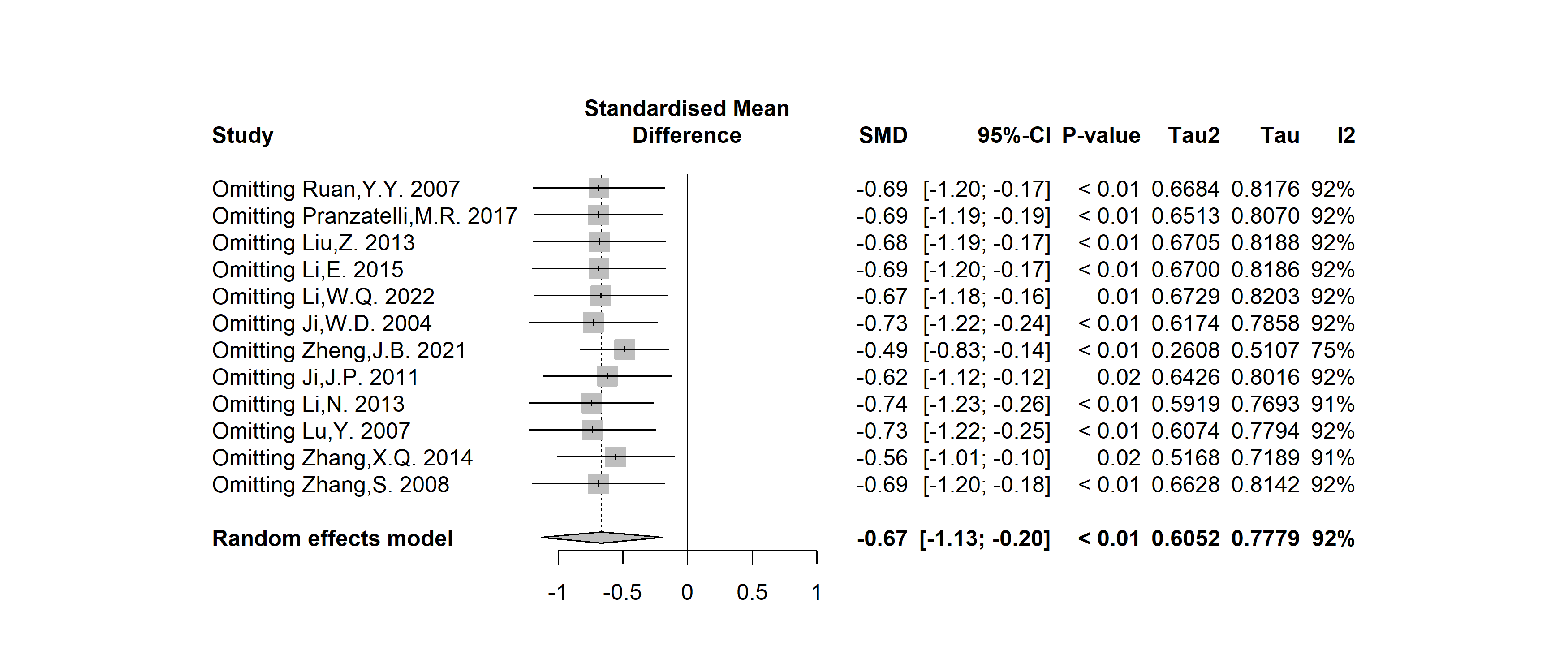


Figure S10 Sensitivity analysis of serum anti-streptolysin O antibodies (a) and anti-DNase B antibodies (b) in TS samples vs controls.

(a)


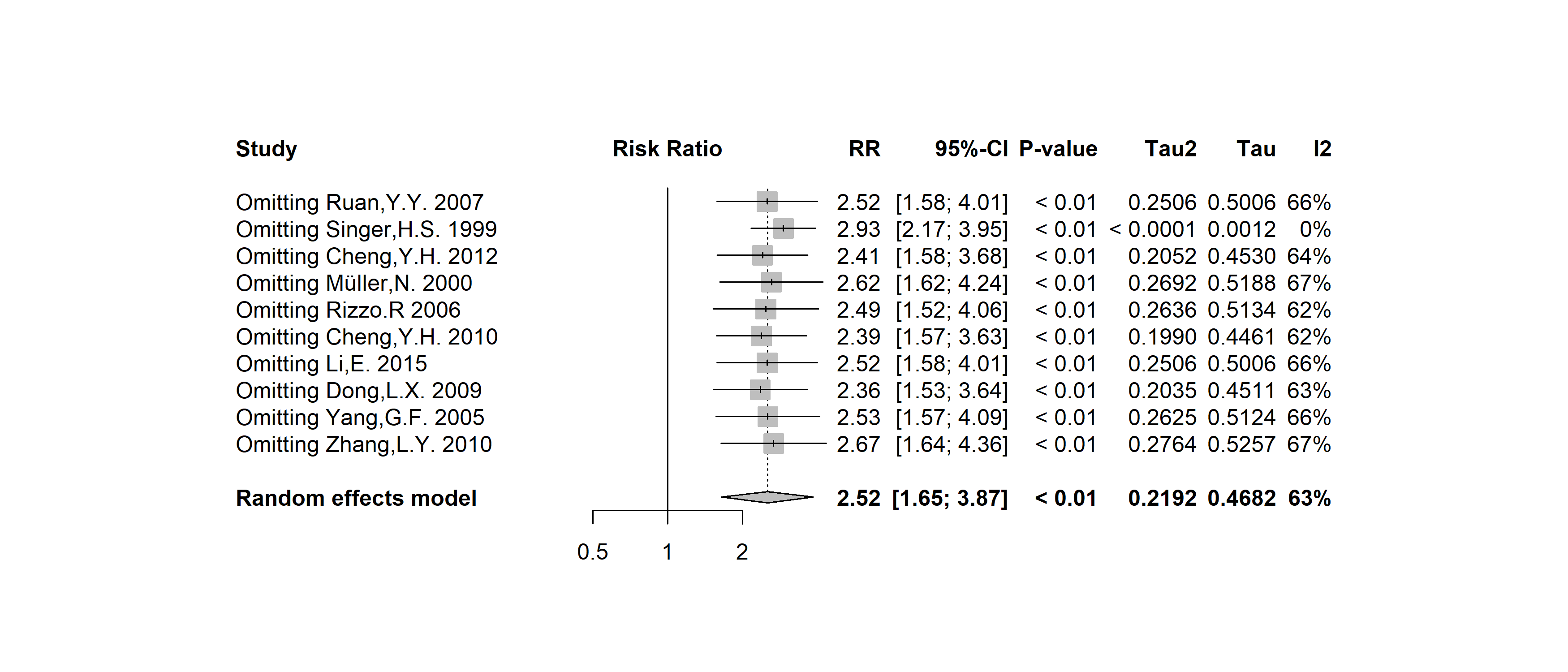


(b)


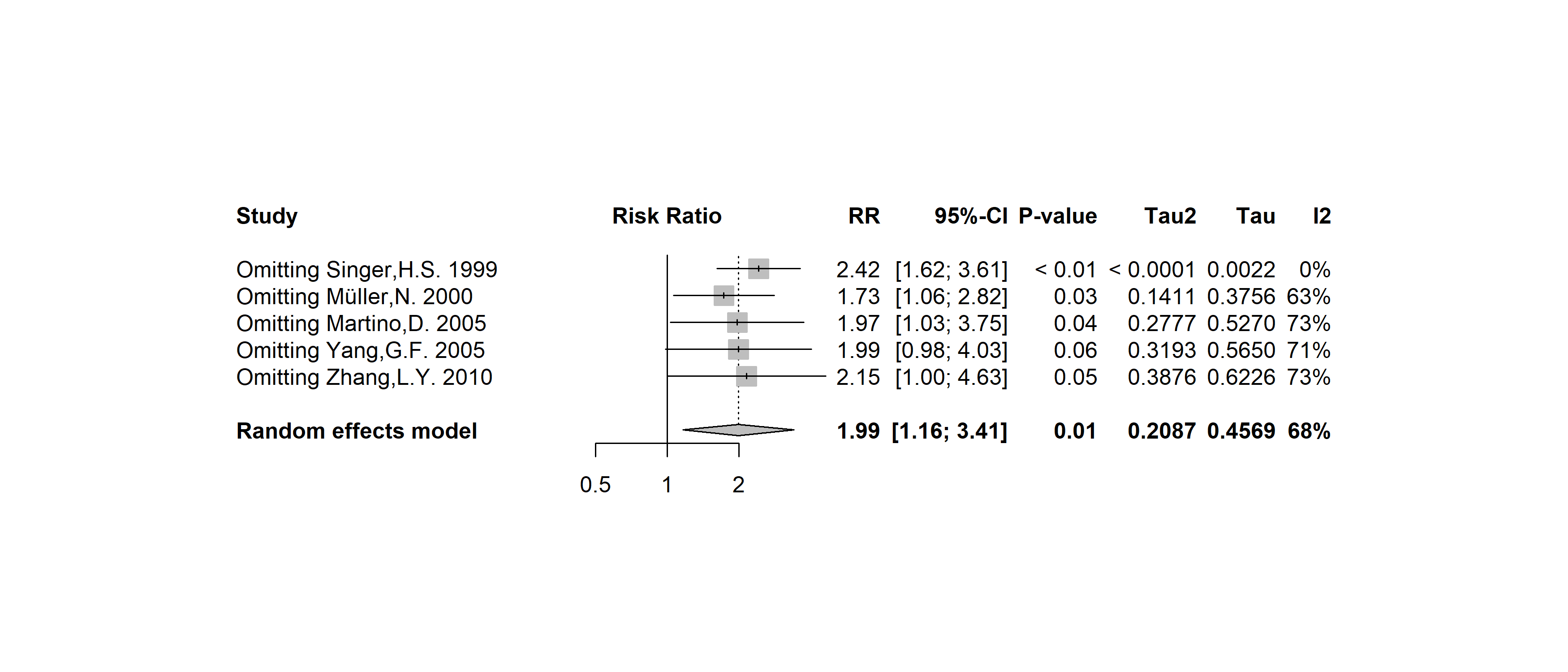


Figure S11 Sensitivity analysis of blood Glu (a) and Asp (b) in TS samples vs controls.

(a)


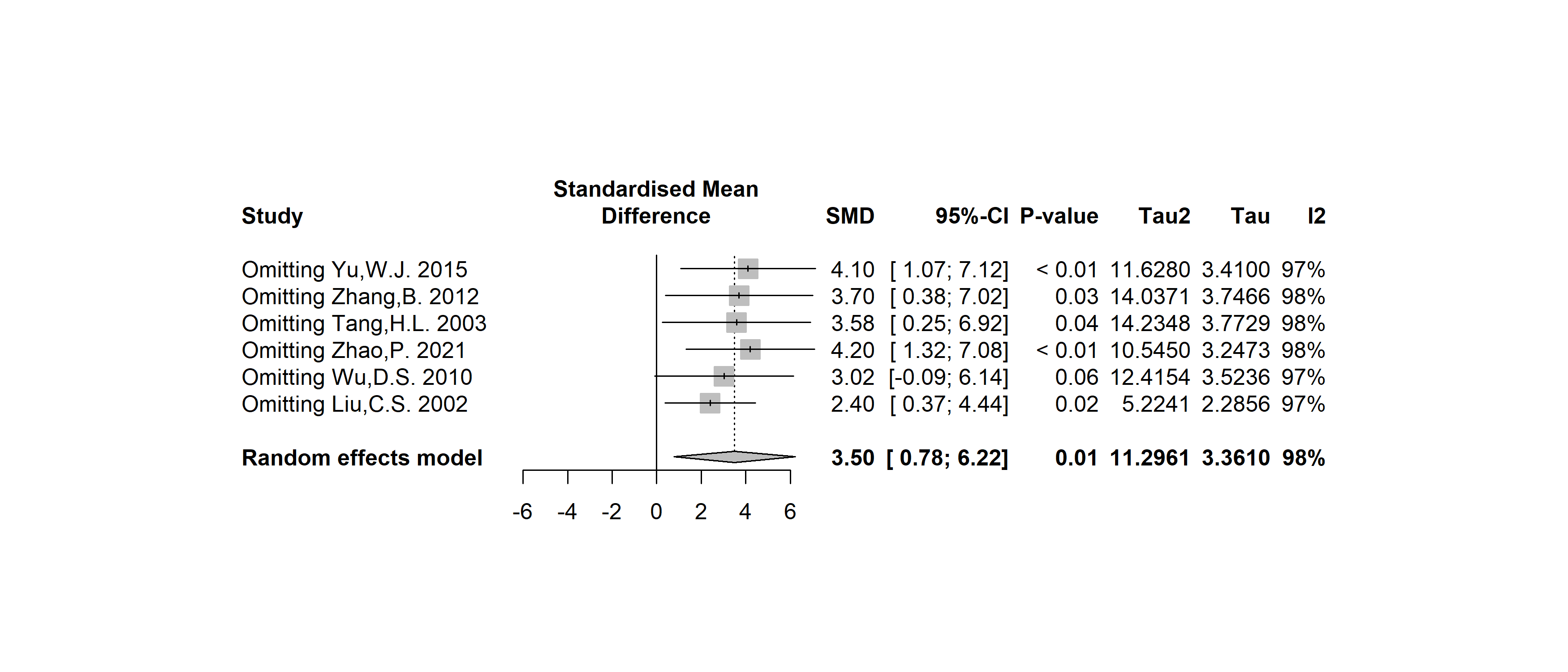


(b)


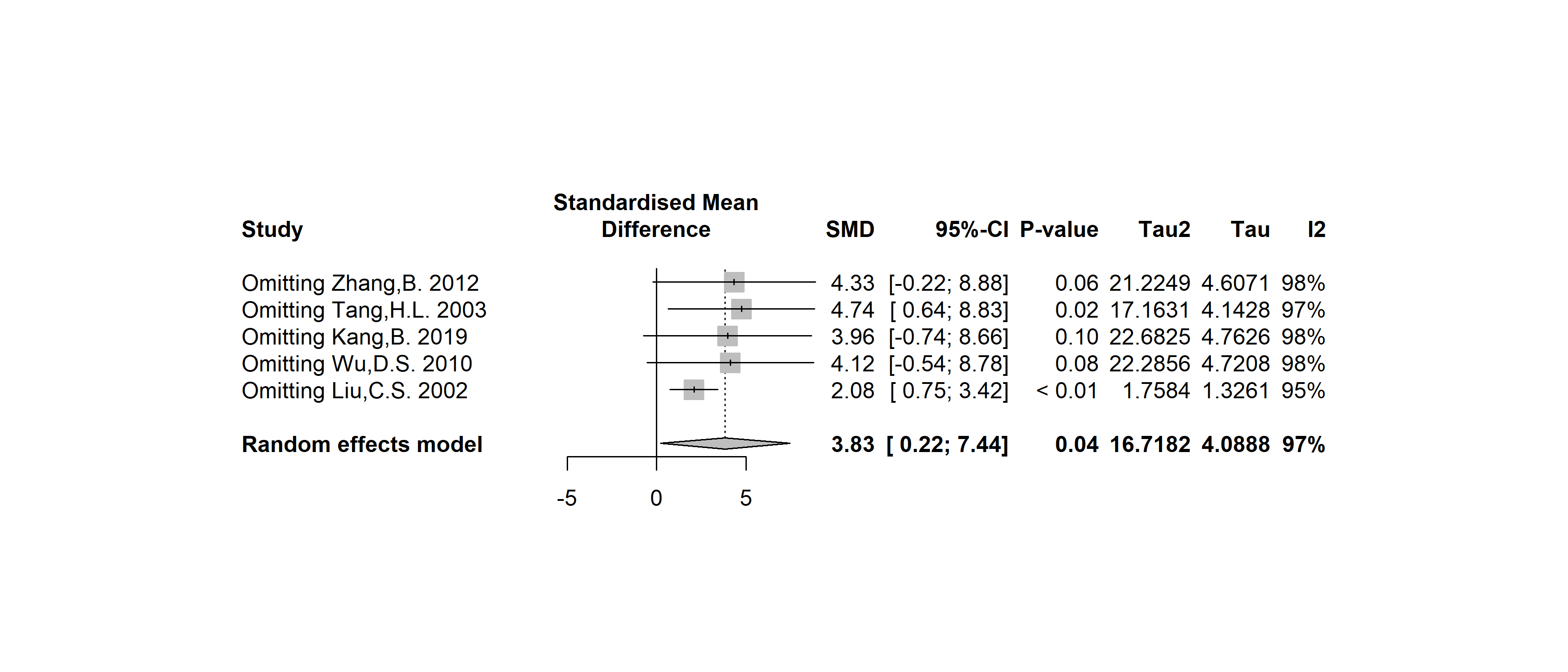


Figure S12 Sensitivity analysis of blood Fe (a), Zn (b) and vitamin D (c) in TS samples vs controls.

(a)


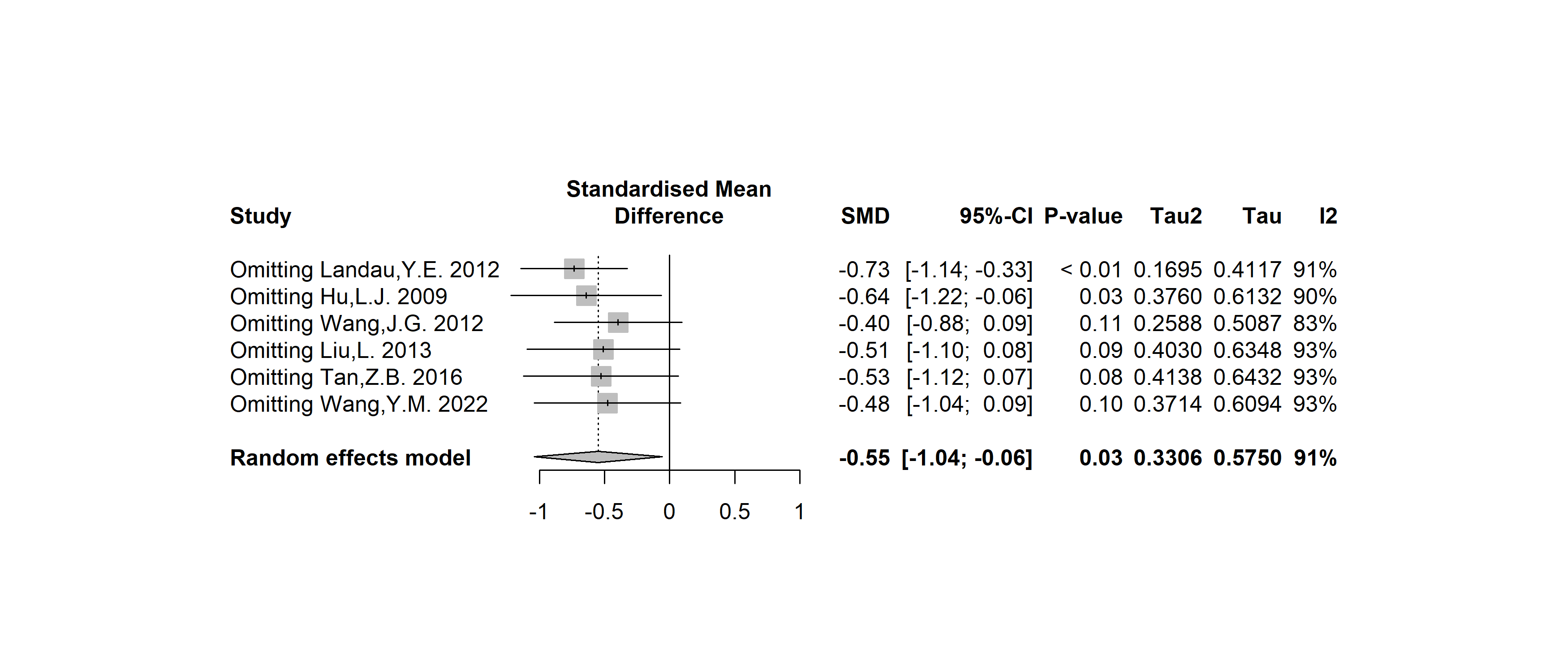


(b)


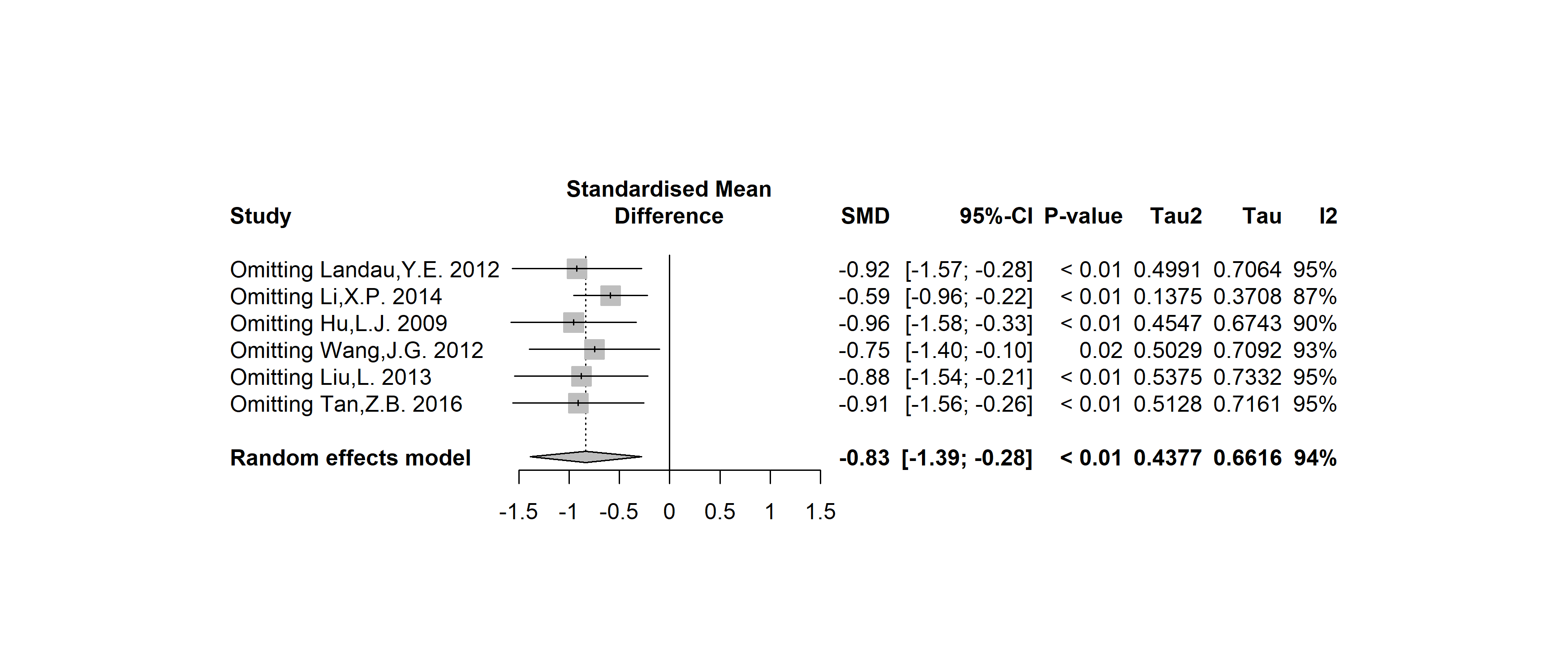


(c)


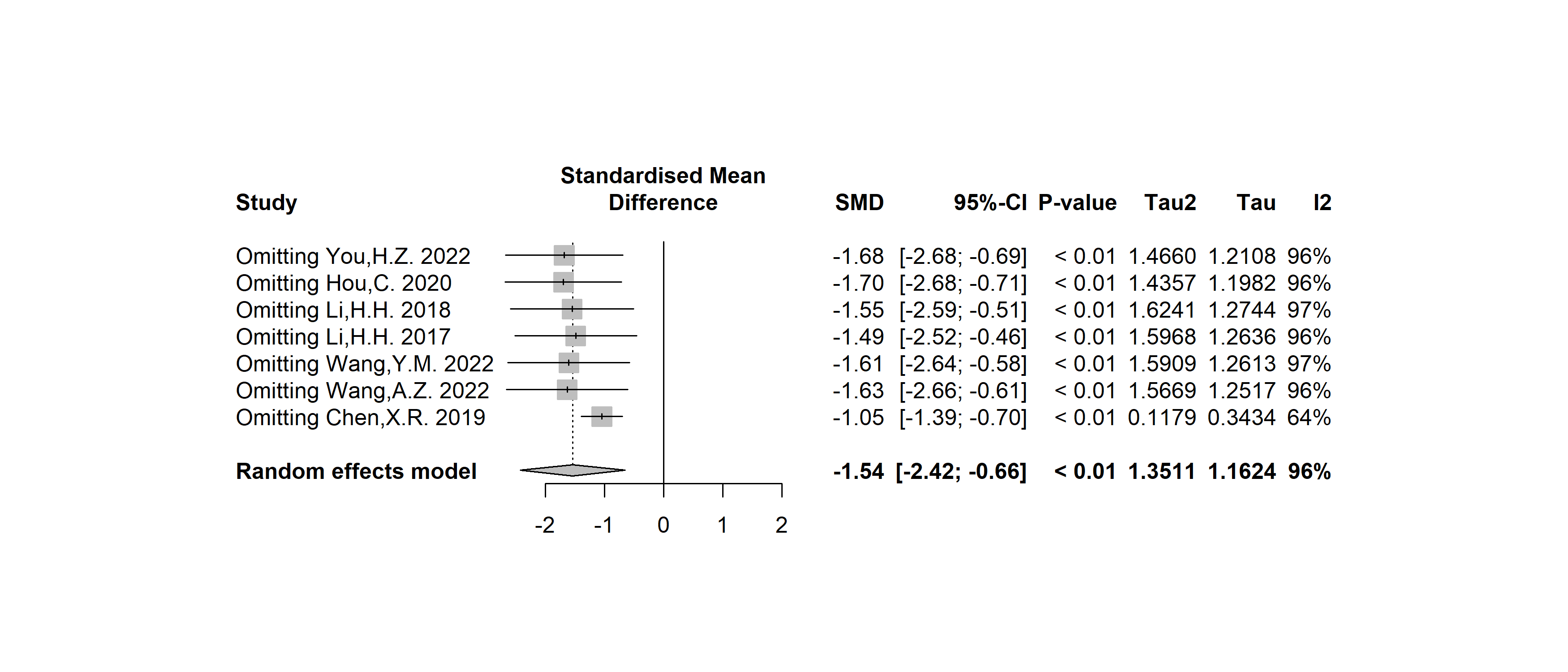


Figure S13 Sensitivity analysis of blood BDNF in TS samples vs controls.


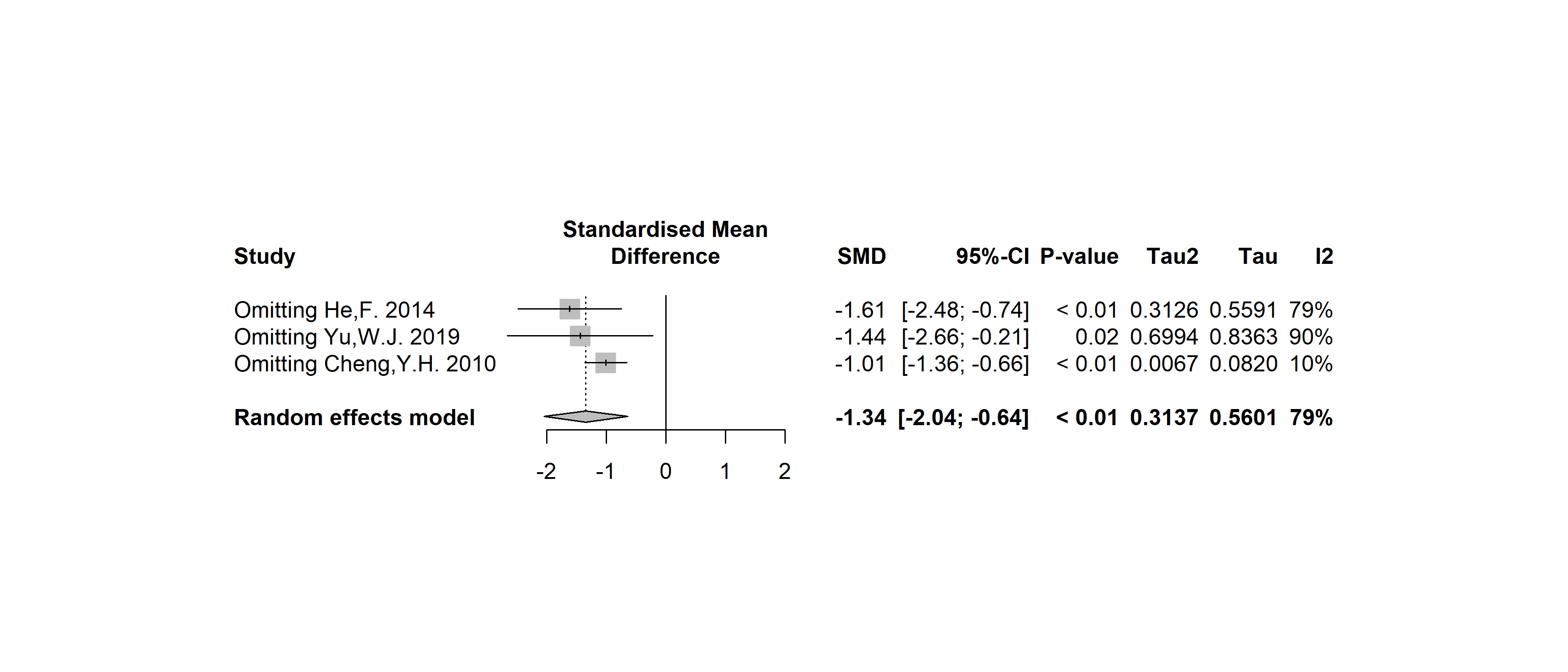

Supplement: Supplementary file 1 [file Data_Sheet_1.docx]
