# Supplementary material for: Biomarkers and Tourette syndrome: a systematic review and meta-analysis
Source: Front Neurol. 2024 Feb 7;15:1262057. doi: 10.3389/fneur.2024.1262057 (PMC10879287; doi:10.3389/fneur.2024.1262057)
Supplement: Supplementary file 2 [file Table_1.docx]

**Table S1: Search strategy used on databases.** Search terms used to identify studies exploring peripheral TS biomarkers in literature databases (Medline/PubMed; Cochrane Library; Embase; Web of Science; CNKI) published from inception until November 2022.

| **Database** | **Search Strategy** |
| --- | --- |
| Medline/PubMed-1561 | ("Tic Disorders"[Title/Abstract] OR "Tics"[Title/Abstract] OR "Tourette"[Title/Abstract] OR "Tourette Syndrome"[Title/Abstract] OR "Tic Disorders"[MeSH Terms] OR "Tics"[MeSH Terms] OR "Tourette Syndrome"[MeSH Terms]) AND ("Biomarkers"[MeSH Terms] OR "Serum"[MeSH Terms] OR "Plasma"[MeSH Terms] OR "Urine"[MeSH Terms] OR "Saliva"[MeSH Terms] OR "Blood"[MeSH Terms] OR "Blood Platelets"[MeSH Terms] OR "Erythrocytes"[MeSH Terms] OR "Hair"[MeSH Terms] OR ("levels"[Title/Abstract] OR "peripheral"[Title/Abstract] OR "Serum"[Title/Abstract] OR "Plasma"[Title/Abstract] OR "Urine"[Title/Abstract] OR "Saliva"[Title/Abstract] OR "Blood"[Title/Abstract] OR "platelets"[Title/Abstract] OR "cerebrospinal fluid"[Title/Abstract] OR "red blood cells"[Title/Abstract] OR "Hair"[Title/Abstract] OR "salivary biomarker*"[Title/Abstract] OR "urinary biomarker*"[Title/Abstract] OR "plasma biomarker*"[Title/Abstract] OR "blood biomarker*"[Title/Abstract] OR "serum biomarker*"[Title/Abstract] OR "biomarker*"[Title/Abstract])) |
| Cochrane Library-403 | (tic disorders OR tics OR Tourette OR Tourette Syndrome) AND (Serum OR Plasma OR Urine OR Saliva OR Blood OR Blood Platelets OR Erythrocytes OR Hair OR levels OR peripheral OR cerebrospinal fluid OR red blood cells OR salivary biomarker* OR urinary biomarker* OR plasma biomarker* OR blood biomarker* OR serum biomarker* OR biomarker*) |
| Embase-4174 | ('serum'/exp OR serum OR plasma:ab,ti OR urine:ab,ti OR saliva:ab,ti OR blood:ab,ti OR 'blood platelets':ab,ti OR erythrocytes:ab,ti OR hair:ab,ti OR levels:ab,ti OR peripheral:ab,ti OR 'cerebrospinal fluid':ab,ti OR 'red blood cells':ab,ti OR 'salivary biomarker*':ab,ti OR 'urinary biomarker*':ab,ti OR 'plasma biomarker*':ab,ti OR 'blood biomarker*':ab,ti OR 'serum biomarker*':ab,ti OR biomarker*:ab,ti) AND ('tic disorders'/exp OR 'tic disorders' OR (('tic'/exp OR tic) AND ('disorders'/exp OR disorders)) OR tics:ab,ti OR 'tourette syndrome':ab,ti OR tourette:ab,ti) |
| Web of Science-9806 | (TI=(tic disorders OR tics OR Tourette OR Tourette Syndrome)) AND TI=(Serum OR Plasma OR Urine OR Saliva OR Blood OR Blood Platelets OR Erythrocytes OR Hair OR levels OR peripheral OR cerebrospinal fluid OR red blood cells OR salivary biomarker* OR urinary biomarker* OR plasma biomarker* OR blood biomarker* OR serum biomarker* OR biomarker*) OR (AB=(tic disorders OR tics OR Tourette OR Tourette Syndrome)) AND AB=(Serum OR Plasma OR Urine OR Saliva OR Blood OR Blood Platelets OR Erythrocytes OR Hair OR levels OR peripheral OR cerebrospinal fluid OR red blood cells OR salivary biomarker* OR urinary biomarker* OR plasma biomarker* OR blood biomarker* OR serum biomarker* OR biomarker*) |
| CNKI-4413 | (tic disorders OR tics OR Tourette OR Multiple Tics OR Tourette Syndrome) AND (biomarker OR peripheral OR blood OR cerebrospinal fluid OR Serum OR Plasma OR Urine OR Hair OR Erythrocytes OR Saliva OR Blood Platelets OR concentration OR levels) |
