# Supplementary material for: Biomarkers and Tourette syndrome: a systematic review and meta-analysis
Source: Front Neurol. 2024 Feb 7;15:1262057. doi: 10.3389/fneur.2024.1262057 (PMC10879287; doi:10.3389/fneur.2024.1262057)
Supplement: Supplementary file 3 [file Table_2.docx]

Table S2 Study characteristics of case-control studies included in the meta-analysis.

| First Author | Year | Population | Biological Fluids | Biomarkers | Levels in  TS groups  (Mean±SD) | Levels in  control groups  (Mean±SD) | Diagnosis | %Male (TS groups) | %Male (control groups) | Age  (patients)  (Mean±SD) | Age  (controls)  (Mean±SD) | NOS scores | Comorbidity |
| --- | --- | --- | --- | --- | --- | --- | --- | --- | --- | --- | --- | --- | --- |
| Gabbay, V. | 2009 | USA | plasma | TNF-α(pg/ml) | 3.67±17.2 | 3.92±13.0 | DSM-IV | 27/32 | 7/16 | 11.2±3.15 | 15.1±3.30 | 7 | OCD |
|  |  |  |  | IL-1β(pg/ml) | 0.23±0.28 | 0.16±0.05 |  |  |  |  |  |  |  |
|  |  |  |  | IL-12(pg/ml) | 1.84±3.87 | 0.55±0.88 |  |  |  |  |  |  |  |
|  |  |  |  | IL-6(pg/ml) | 0.68±0.91 | 0.74±1.33 |  |  |  |  |  |  |  |
| Ruan,Y.Y. | 2007 | China | serum | ASO-positive | 13/58 | 4/53 | DSM-IV | 43/58 | NA/53 | 9.66±2.49 | NA | 6 | ADHD |
|  |  |  |  | CD3+(%) | 63.33±10.63 | 67.87±5.95 |  | 43/58 | NA/45 |  |  |  |  |
|  |  |  |  | CD4+(%) | 30.36±6.25 | 33.09±6.69 |  |  |  |  |  |  |  |
|  |  |  |  | CD8+(%) | 30.95±10.43 | 28.00±5.92 |  |  |  |  |  |  |  |
|  |  |  |  | CD4+/CD8+ | 1.08±0.39 | 1.25±0.37 |  |  |  |  |  |  |  |
|  |  |  |  | CD19+(%) | 14.36±4.62 | 14.22±5.06 |  |  |  |  |  |  |  |
|  |  |  |  | NK cells(%) | 20.24±9.31 | 16.98±6.43 |  |  |  |  |  |  |  |
| Singer,H.S. | 1999 | USA | serum | ASO-positive | 19/41 | 20/39 | Tourette Syndrome Classificat-ion Study Group (1993) | 33/41 | 22/39 | 11.3 | 12.1 | 7 |  |
|  |  |  |  | AntiDNAase-positive | 25/41 | 23/39 |  |  |  |  |  |  |  |
| Pranzatelli,M.R. | 2017 | USA | serum | CD4+/CD8+ | 1.8±0.5 | 2.0±0.6 | DSM-V | 3/5 | NA/26 | 10±2 | NA | 7 |  |
|  |  |  |  | NK cells(%) | 11±7 | 10±6 |  |  |  |  |  |  |  |
| You,H.Z. | 2022 | China | serum | VD(ng/ml) | 21.67±6.11 | 26.14±6.48 | DSM-V | NA/24 | 48/180 | NA | 8.18±2.39 | 7 |  |
| Liu,Z. | 2013 | China | serum | CD3+(%) | 62.96±7.27 | 61.34±7.54 | DSM-IV | 45/57 | 30/43 | 9.68±1.35 | 9.37±1.08 | 8 |  |
|  |  |  |  | CD4+(%) | 27.11±5.52 | 30.22±6.68 |  |  |  |  |  |  |  |
|  |  |  |  | CD8+(%) | 30.59±5.10 | 29.91±5.34 |  |  |  |  |  |  |  |
|  |  |  |  | CD4+/CD8+ | 0.91±0.24 | 1.03±0.25 |  |  |  |  |  |  |  |
| Weisz,J.L. | 2004 | USA | serum | CD19+(%) | 17.70±5.59 | 11.06±2.52 | DSM-IV | NA/33 | NA/98 | NA | NA | 6 |  |
| Cheng,Y.H. | 2012 | China | plasma | IL-1β(ng/mL) | 1.19±0.73 | 0.12±0.18 | DSM-IV | 28/40 | 25/40 | 12.95±3.28 | 12.38±3.03 | 8 |  |
|  |  |  |  | IL-6 (ng/mL) | 1.72±0.89 | 0.43±0.43 |  |  |  |  |  |  |  |
|  |  |  |  | ASO-positive | 8/40 | 0/40 |  |  |  |  |  |  |  |
| Ruan,Y.Y.^a^ | 2009 | China | serum | IL-6(ng/L) | 7.18±14.55 | 15.93±12.18 | DSM-IV | 43/58 | NA/30 | 9.66±2.49 | NA | 7 |  |
|  |  |  |  | IL-8(ng/L) | 52.15±48.15 | 222.74±121.05 |  |  |  |  |  |  |  |
|  |  |  |  | TNF-α(ng/L) | 121.61±201.45 | 26.62±48.99 |  |  |  |  |  |  |  |
| Tang.H.X.^b^ | 2014 | China | serum | IL-12（pg/ml) | 124.73±24.03 | 64.56±27.59 | DSM-IV | 75/90 | 26/30 | 10.68±3.01 | 10.80±3.30 | 6 |  |
|  |  |  |  | TNF-α（pg/ml) | 207.90±23.28 | 78.13±33.42 |  |  |  |  |  |  |  |
| Zhang,B.^b^ | 2012 | China | serum | DA（ng/mL） | 6.61±2.49 | 8.54±8.12 | DSM-IV | 55/60 | 15/30 | 7.7±1.2 | 7.3±5.1 | 6 |  |
|  |  |  |  | 5-HT(ng/mL) | 80.30±47.78 | 34.33±24.51 |  |  |  |  |  |  |  |
|  |  |  |  | NE（ng/mL） | 24.68±15.00 | 29.06±22.61 |  |  |  |  |  |  |  |
|  |  |  |  | Glu（μg/mL） | 23.56±7.25 | 6.28±5.45 |  |  |  |  |  |  |  |
|  |  |  |  | Asp（μg/mL） | 4.05±1.71 | 1.08±1.05 |  |  |  |  |  |  |  |
|  |  |  |  | GABA  （μg/mL） | 1.60±1.46 | 4.90±1.22 |  |  |  |  |  |  |  |
| Müller,N. | 2000 | Germany | serum | ASO-positive | 9/13 | 4/13 | DSM-III | 9/13 | 8/13 | 12±2 | 12±3 | 8 |  |
|  |  |  |  | AntiDNAse-positive | 11/13 | 1/13 |  |  |  |  |  |  |  |
| Rizzo.R | 2006 | Italy | serum | ASO-positive | 41/69 | 14/72 | DSM-IV | 64/69 | 65/72 | 9.36±3.18 | 8.75±2.75 | 7 |  |
| Landau,Y.E.^a^ | 2012 | Israel | serum | Fe (ng/ml) | 30.6±22.2 | 22.2±13.4 | DSM-IV | 58/68 | 25/36 | 11.4±3 | 10.9±2.9 | 7 |  |
|  |  |  |  | Zn (mg/dL) | 79.3±10.8 | 83.2±8.2 |  |  |  |  |  |  |  |
|  |  |  |  | IgA (mg/dL) | 124.38±67.03 | 154.34±73.26 |  |  |  |  |  |  |  |
|  |  |  |  | IgM (mg/dL) | 115.99±42.48 | 101.92±40.50 |  |  |  |  |  |  |  |
|  |  |  |  | IgG(mg/dL) | 1086.28±171.14 | 1089.92±286.33 |  |  |  |  |  |  |  |
| Li,H.H. | 2018 | China | serum | VD (ng/mL) | 16.8 ± 4.6 | 28.9±8.3 | DSM-V | NA/10 | 154/189 | NA | 8.1±2.6 | 8 |  |
| Li,H.H. | 2017 | China | serum | VD(ng/mL) | 17±8 | 32±8 | DSM-V | NA/8 | 117/144 | NA | 8.3±1.9 | 7 |  |
| Martino,D.^a^ | 2005 | UK | serum | Anti-DNAse-positive | 10/33 | 4/34 | DSM-IV | 26/33 | 24/34 | 12.13±3.80 | 9.58±3.85 | 6 |  |
| Cheng,Y.H. | 2010 | China | serum | ASO-positive | 13/66 | 0/60 | CCMD-3 | 55/67 | 50/64 | 11.8±3.2 | 14.0±2.3 | 8 |  |
| Li,E. | 2015 | China | serum | ASO-positive | 13/58 | 4/53 | DSM-IV | 43/58 | 82/128 | 9.7±2.5 | 8.9±2.7 | 7 |  |
|  |  |  |  | CD3+ (%) | 63.33±10.63 | 67.87±5.95 |  | 43/58 | NA/45 |  |  |  |  |
|  |  |  |  | CD4+ (%) | 30.36±6.25 | 33.09±6.69 |  | 43/58 | NA/45 |  |  |  |  |
|  |  |  |  | CD8+ (%) | 30.95±10.43 | 28.00±5.92 |  | 43/58 | NA/45 |  |  |  |  |
|  |  |  |  | CD4+/CD8+ | 1.08±0.39 | 1.25±0.37 |  | 43/58 | NA/45 |  |  |  |  |
|  |  |  |  | CD19+ (%) | 14.36±4.62 | 14.22±5.06 |  | 43/58 | NA/45 |  |  |  |  |
|  |  |  |  | NK cells (%) | 20.24±9.31 | 16.98±6.43 |  | 43/58 | NA/45 |  |  |  |  |
| Zhang,F.H. | 2002 | China | serum | PRL (ng/ml) | 10.18±8.80 | 21.04±2.50 | DSM-IV | 52/62 | 11/15 | 7.0±2.3 | 6.8±2.4 | 6 |  |
| Li,W.Q. | 2022 | China | serum | CD3+ (%) | 65.6±6.02 | 70.8±4.2 | DSM-V | NA/23 | 115/150 | NA | 7.4±2.2 | 7 |  |
|  |  |  |  | CD4+/CD8+ | 1.34±0.43 | 1.99±1.15 |  |  |  |  |  |  |  |
| Ji,W.D. | 2004 | China | serum | CD3+ (%) | 54.3±9.0 | 60.4±9.2 | DSM-IV | NA/17 | 22/30 | NA | 10.7±2.3 | 7 |  |
|  |  |  |  | CD4+ (%) | 38.4±6.8 | 41.7±7.6 |  |  |  |  |  |  |  |
|  |  |  |  | CD8+ (%) | 25.8±6.4 | 26.4±6.2 |  |  |  |  |  |  |  |
|  |  |  |  | CD4+/CD8+ | 1.60±0.72 | 1.57±0.46 |  |  |  |  |  |  |  |
|  |  |  |  | IL-6 (ng/L) | 71.24±28.56 | 58.48±26.21 |  |  |  |  |  |  |  |
|  |  |  |  | IL-8(g/L) | 65.12±27.58 | 56.84±22.78 |  |  |  |  |  |  |  |
| Zhang,J.Z. | 2014 | China | serum | IL-1β(KU/L) | 54.39±6.71 | 55.14±9.29 | DSM-IV | 38/41 | 53/60 | 10±3 | 10±3 | 7 |  |
|  |  |  |  | IL-4(KU/L) | 403.05±55.62 | 309.53±63.76 |  |  |  |  |  |  |  |
|  |  |  |  | IL-12(KU/L) | 31.62±8.58 | 40.16±15.24 |  |  |  |  |  |  |  |
|  |  |  |  | IFN-γ(KU/L) | 294.79±34.61 | 323.48±55.73 |  |  |  |  |  |  |  |
| Zheng,J.B. | 2021 | China | serum | Cor（nmol/L） | 202.34 ± 43.74 | 396.22 ± 81.03 | ESCAP | 94/135 | 46/62 | 10.25±2.04 | 10.67±2.15 | 7 | ADHD |
|  |  |  |  | IL-8（ng/L） | 114.76 ± 19.02 | 53.48 ± 13.29 |  |  |  |  |  |  |  |
|  |  |  |  | IFN-γ（μg/L） | 7. 57±1.64 | 1. 82 ± 0. 73 |  |  |  |  |  |  |  |
|  |  |  |  | CD4+ (%) | 24.98 ± 3.02 | 41. 47 ± 5. 13 |  |  |  |  |  |  |  |
|  |  |  |  | CD4+/CD8+ | 0. 98 ± 0.16 | 1. 52 ± 0. 31 |  |  |  |  |  |  |  |
| Liu,Z.S.^b^ | 1995 | China | plasma | PRL (ng/ml) | 14.01±8.19 | 5.11±0.46 | DSM-III | 33/39 | 11/18 | NA | NA | 6 |  |
| Li，J. | 2013 | China | serum | PRL (ng/ml) | 201.66±112.52 | 260.13±196.80 | DSM-IV | 25/31 | 19/30 | 10.09±2.14 | 9.58±1.60 | 8 | ADHD |
| He,F.^b^ | 2014 | China | serum | BDNF(ng/ml) | 16.16±10.49 | 23.89±5.41 | DSM-IV | 50/60 | 22/28 | 11.47±2.24 | 10.68±1.867 | 8 |  |
| Ji,J.P. | 2011 | China | serum | CD3+ (%) | 62.24±8.05 | 61.57±7.79 | DSM-IV | NA/16 | 17/30 | NA | 9.57±0.86 | 8 |  |
|  |  |  |  | CD4+ (%) | 23.98±3.41 | 30.42±6.65 |  |  |  |  |  |  |  |
|  |  |  |  | CD8+ (%) | 31.82±6.04 | 30.01±5.48 |  |  |  |  |  |  |  |
|  |  |  |  | CD4+/CD8+ | 0.77±0.16 | 1.04±0.25 |  |  |  |  |  |  |  |
|  |  |  |  | NK cells (%) | 11.69±5.06 | 10.04±4.57 |  |  |  |  |  |  |  |
| Dong,L.X. | 2009 | China | serum | ASO-positive | 27/48 | 2/20 | DSM-IV | 31/48 | 12/20 | 9.9±1.1 | 9.9±1.3 | 7 |  |
| Li,N. | 2013 | China | serum | CD3+ (%) | 57.5±7.8 | 60.4±9.2 | DSM-IV | 23/32 | 20/30 | 10.1±2.8 | 10.7±2.3 | 6 |  |
|  |  |  |  | CD4+ (%) | 37.4±7.1 | 41.7±7.6 |  |  |  |  |  |  |  |
|  |  |  |  | CD8+ (%) | 24.2±5.7 | 26.4±6.2 |  |  |  |  |  |  |  |
|  |  |  |  | CD4+/CD8+ | 1.58±0.62 | 1.48±0.41 |  |  |  |  |  |  |  |
|  |  |  |  | IL-6(ng/ml) | 68.12±28.13 | 48.48±26.21 |  |  |  |  |  |  |  |
|  |  |  |  | IL-8(ng/ml) | 63.28±26.63 | 56.84±22.78 |  |  |  |  |  |  |  |
| Lu,Y. | 2007 | China | serum | IgG(g/L) | 9.66±1.92 | 10.71±1.66 | CCMD-3 | 18/21 | 25/30 | 9.8±2.3 | 10.1±2.1 | 7 |  |
|  |  |  |  | IgA(g/L) | 1.53±0.52 | 1.27±0.27 |  |  |  |  |  |  |  |
|  |  |  |  | IgM(g/L) | 1.37±0.53 | 1.71±0.37 |  |  |  |  |  |  |  |
|  |  |  |  | C3(g/L) | 1.09±0.28 | 1.07±0.19 |  |  |  |  |  |  |  |
|  |  |  |  | C4(g/L) | 0.24±0.12 | 0.19±0.06 |  |  |  |  |  |  |  |
|  |  |  |  | CD3+ (%) | 59.32±6.03 | 61.75±4.69 |  |  |  |  |  |  |  |
|  |  |  |  | CD4+ (%) | 34.9±5.37 | 36.13±4.3 |  |  |  |  |  |  |  |
|  |  |  |  | CD8+ (%) | 23.34±6.30 | 25.81±7.61 |  |  |  |  |  |  |  |
|  |  |  |  | CD4+/CD8+ | 1.67±0.74 | 1.59±0.69 |  |  |  |  |  |  |  |
| Liu,H.Z. | 2005 | China | serum | IgG（mg/dl) | 1001.17±206.94 | 1004.40±224.62 | CCMD-3 | 39/49 | 26/30 | 11.3±2.8 | 12.1±2.7 | 7 |  |
|  |  |  |  | IgA（mg/dl) | 155.90±50.37 | 158.17±52.98 |  |  |  |  |  |  |  |
|  |  |  |  | IgM（mg/dl) | 138.78±51.18 | 131.65±53.61 |  |  |  |  |  |  |  |
|  |  |  |  | C3（mg/dl) | 109.88±23.51 | 107.13±23.53 |  |  |  |  |  |  |  |
|  |  |  |  | C4（mg/dl) | 18.85±6.80 | 18.63±7.12 |  |  |  |  |  |  |  |
| Li,X.P. | 2014 | China | plasma | DA (ng/L) | 30.47±18.26 | 62.52±17.65 | DSM-IV | NA/40 | 73/109 | NA | 9.3±3.1 | 7 |  |
|  |  |  |  | NE (ng/L) | 112.35±87.28 | 182.73±60.38 |  |  |  |  |  |  |  |
|  |  |  |  | 5-HT (pg/L) | 158.23±47.62 | 101.45±76.28 |  |  |  |  |  |  |  |
|  |  |  |  | Zn(mol/L) | 7.02±1.35 | 10.25±1.63 |  |  |  |  |  |  |  |
|  |  |  |  | Ca(mol/L) | 1.86±0.93 | 2.25±1.02 |  |  |  |  |  |  |  |
| Hou,C. | 2020 | China | serum | VD (mg/l) | 21.83±7.60 | 26.61±7.59 | DSM-V | NA/83 | 50/63 | NA | 7.36±2.41 | 7 |  |
| Xiao,G.H. | 2008 | China | serum | DA(μg/L) | 0.451±0.336 | 0.210±0.106 | DSM-IV | NA/11 | 33/40 | NA | NA | 7 |  |
|  |  |  |  | NE(μg/L) | 0.212±0.136 | 0.127±0.086 |  |  |  |  |  |  |  |
| Yang,G.F. | 2005 | China | serum | ASO-positive | 20/80 | 7/80 | DSM-IV | 65/80 | 61/80 | 9.8±2.1 | 9.5±2.0 | 7 |  |
|  |  |  |  | AntiDNAase-positive | 22/80 | 9/80 |  |  |  |  |  |  |  |
| Hu,L.J. | 2009 | China | serum | Cu（μg/ml） | 1.32±0.31 | 1.29±0.32 | DSM-IV | 312/404 | NA/737 | 9.92±3.15 | NA | 6 |  |
|  |  |  |  | Zn（μg/ml） | 7.81±1.27 | 8.17±1.32 |  |  |  |  |  |  |  |
|  |  |  |  | Fe（μg/ml） | 340.28±28.47 | 345.09±31.53 |  |  |  |  |  |  |  |
|  |  |  |  | Ca（μg/ml） | 56.26±5.48 | 55.71±6.14 |  |  |  |  |  |  |  |
|  |  |  |  | Mg（μg/ml） | 33.04±4.50 | 33.07±4.52 |  |  |  |  |  |  |  |
|  |  |  |  | Pb（μg/ml） | 0.0510±0.0151 | 0.0476±0.0182 |  |  |  |  |  |  |  |
| Wang,J.G. | 2012 | China | serum | Ca (mmo/L) | 2.35±0.16 | 2.41±0.12 | DSM-IV | 73/90 | 70/80 | NA | NA | 7 |  |
|  |  |  |  | Fe (molL) | 8.8±3.8 | 14.2±4.9 |  |  |  |  |  |  |  |
|  |  |  |  | Zn (mol/L) | 11.7±2.6 | 14.8±2.3 |  |  |  |  |  |  |  |
| Zhang,L.Y. | 2010 | China | serum | AntiDNAase-positive | 22/39 | 11/40 | DSM-IV | 30/39 | 29/40 | 7.7±0.6 | 10.9±0.9 | 7 |  |
|  |  |  |  | ASO-positive | 16/39 | 8/40 |  |  |  |  |  |  |  |
| Tang,H.L. | 2003 | China | plasma | Glu(μmol/L) | 27.454±7.498 | 12.074±1.927 | DSM-IV | NA/15 | 18/25 | 7.2±3.8 | 6.4±5.2 | 7 |  |
|  |  |  |  | Asp(μmol/L) | 11.262±1.735 | 10.739±2.351 |  |  |  |  |  |  |  |
| Zhao,P.^a^ | 2021 | China | plasma | DA（nmol/L) | 0.18±0.02 | 0.08±0.01 | DSM-V | NA/8 | 47/60 | NA | 8.40±2.43 | 7 |  |
|  |  |  |  | NE（nmol/L) | 2.64±1.34 | 2.72±3.32 |  |  |  |  |  |  |  |
|  |  |  |  | 5-HT（nmol/L) | 295.47±170.83 | 319.20±189.42 |  |  |  |  |  |  |  |
|  |  |  |  | Glu（nmol/L)×10*^4^* | 5.18±1.94 | 5.18±2.69 |  |  |  |  |  |  |  |
|  |  |  |  | GABA（nmol/L) | 176.70±111.77 | 159.72±164.62 |  |  |  |  |  |  |  |
| Wang,Y.M. | 2022 | China | serum | Fe（μmol/L） | 12.14±4.38 | 16.93±5.02 | DSM-V CCMD-3 | NA/14 | 42/54 | NA | 5.69±2.20 | 7 |  |
|  |  |  |  | VD（ng/mL) | 17.54±10.10 | 27.49±8.30 |  |  |  |  |  |  |  |
| Wen,X.M. | 2012 | China | plasma | DA (ng/mL) | 129.4±32.3 | 45.39±13.48 | DSM-IV | 52/60 | 20/30 | NA | NA | 7 |  |
| Gao,C. | 2016 | China | serum | IFN-γ（μg/L) | 9.75±3.62 | 1.92±3.26 | Diagnosis and treatment of children with tic disorder（2013） | 28/40 | 30/40 | 8.8±1.5 | 8.2±1.4 | 7 |  |
|  |  |  |  | IL-4（μg/L) | 40.92±4.92 | 47.79±5.16 |  |  |  |  |  |  |  |
| Yu,W.J. | 2015 | China | serum | DA (ng/mL) | 0.26±0.18 | 0.09±0.02 | DSM-IV | 65/80 | 40/60 | 8.8±2.3 | 8.5±3.4 | 7 |  |
|  |  |  |  | NE (ng/mL) | 0.56±0.45 | 0.28±0.16 |  |  |  |  |  |  |  |
|  |  |  |  | Glu (μg/mL) | 13.22±11.23 | 8.32±2.44 |  |  |  |  |  |  |  |
|  |  |  |  | GABA (μg/mL) | 1.29±1.16 | 1.47±1.08 |  |  |  |  |  |  |  |
| Yu,W.J. | 2019 | China | serum | BDNF (ng/mL) | 2.23±1.07 | 3.35±0.58 | DSM-V | 43/56 | 22/30 | 9.4±2.9 | 8.9±3.2 | 7 |  |
| Kang,B.^b^ | 2019 | China | serum | DA(μg/L) | 6.50±2.40 | 8.65±2.23 | DSM-IV | 69/80 | 35/40 | 7.55±1.10 | 7.8±1.1 | 7 |  |
|  |  |  |  | 5-HT(μg/L) | 80.4±17.58 | 34.22±14.40 |  |  |  |  |  |  |  |
|  |  |  |  | NE(μg/L) | 23.67±8.18 | 30.17±12.50 |  |  |  |  |  |  |  |
|  |  |  |  | Asp(mg/L) | 4.20±1.08 | 1.06±0.45 |  |  |  |  |  |  |  |
|  |  |  |  | GABA(mg/L) | 1.40±0.37 | 4.96±1.11 |  |  |  |  |  |  |  |
|  |  |  |  | Cor(μg/L) | 28.65±6.80 | 18.24±5.64 |  |  |  |  |  |  |  |
| Liu,L. | 2013 | China | serum | Cu(ug/L) | 971±150 | 994±142 | CCMD-3 | NA/23 | 29/40 | NA | 6.9±2.3 | 7 |  |
|  |  |  |  | Mg(mg/L) | 35±6 | 34±7 |  |  |  |  |  |  |  |
|  |  |  |  | Zn(mg/L) | 4.5±1.5 | 5.3±1.2 |  |  |  |  |  |  |  |
|  |  |  |  | Fe(mg/L) | 386±76 | 439±63 |  |  |  |  |  |  |  |
|  |  |  |  | Pb(μg/L) | 95±32 | 65±25 |  |  |  |  |  |  |  |
| Gao,C. | 2019 | China | serum | IFN-γ(KU/L) | 254.56±44.27 | 316.42±52.16 | DSM-V | 25/42 | NA/42 | 9.2±2.3 | NA | 7 |  |
|  |  |  |  | IL-4(KU/L) | 407.38±72.24 | 305.67±58.84 |  |  |  |  |  |  |  |
| Wu,D.S. | 2010 | China | serum | Glu（μmol/L) | 87.56±13.29 | 23.48±5.64 | DSM-IV | 28/40 | 20/30 | 9.6±2.1 | 9.8±2.4 | 7 |  |
|  |  |  |  | Asp（μmol/L) | 59.62±13.52 | 27.54±7.86 |  |  |  |  |  |  |  |
| Wang,A.Z. | 2022 | China | serum | VD (ng/mL) | 26.49±7.73 | 34.55±8.51 | DSM-V | NA/78 | 40/50 | NA | 9.87±2.92 | 7 |  |
| Zhang,X.Q. | 2014 | China | serum | CD3+ (%) | 51.1756±3.998 | 58.062±4.198 | DSM-IV  ICD-10  CCMD-3 | 22/31 | 17/30 | 9.03±2.14 | 8.03±1.16 | 7 |  |
|  |  |  |  | CD4+ (%) | 26.614±4.050 | 28.443±3.262 |  |  |  |  |  |  |  |
|  |  |  |  | CD8+ (%) | 22.681±3.949 | 19.845±1.401 |  |  |  |  |  |  |  |
|  |  |  |  | CD4+/CD8+ | 1.061±0.214 | 1.437±0.168 |  |  |  |  |  |  |  |
| Chen,X.R. | 2019 | China | serum | VD(ng/ml) | 22.79±2.80 | 33. 28±2. 42 | DSM-V | NA/80 | 63/111 | NA | 7. 35±2. 19 | 7 |  |
| Tan,Z.B. | 2016 | China | serum | Fe(mg/L) | 0.9276±0.3117 | 1.1328±0.2856 | DSM-V | 35/35 | 35/35 | NA | NA | 7 |  |
|  |  |  |  | Ca(mg/L) | 83.5289±4.602 | 82.2953±4.580 |  |  |  |  |  |  |  |
|  |  |  |  | Mg(mg/L) | 18.3571±2.255 | 18.3633±2.485 |  |  |  |  |  |  |  |
|  |  |  |  | Cu(mg/L) | 1.0976±0.2779 | 1.1604±0.2629 |  |  |  |  |  |  |  |
|  |  |  |  | Pb(mg/L) | 81.5×10^-3^±21 | 71.5×10^-3^±16 |  |  |  |  |  |  |  |
|  |  |  |  | Zn(mg/L) | 0.7606±1.1474 | 1.1324±0.2248 |  |  |  |  |  |  |  |
| Zhang,S. | 2008 | China | serum | IgG(g/L) | 10.04±2.48 | 11.13±1.89 | CCMD-3 | 25/30 | 24/30 | 10.1±2.7 | 10.5±2.1 | 7 |  |
|  |  |  |  | IgA(g/L) | 1.81±1.01 | 1.91±0.63 |  |  |  |  |  |  |  |
|  |  |  |  | IgM(g/L) | 1.36±038 | 1.26±0.48 |  |  |  |  |  |  |  |
|  |  |  |  | C3(g/L) | 1.02±0.28 | 1.15±0.18 |  |  |  |  |  |  |  |
|  |  |  |  | C4(g/L) | 0.21±0.05 | 0.20±0.05 |  |  |  |  |  |  |  |
|  |  |  |  | CD3+ (%) | 57.97±11.97 | 65.51±3.11 |  |  |  |  |  |  |  |
|  |  |  |  | CD4+ (%) | 28.15±6.54 | 41.73±8.33 |  |  |  |  |  |  |  |
|  |  |  |  | CD8+ (%) | 23.63±7.83 | 29.65±5.20 |  |  |  |  |  |  |  |
|  |  |  |  | CD4+/CD8+ | 1.286±0.39 | 1.397±0.12 |  |  |  |  |  |  |  |
| Mao,Y.Y. | 2008 | China | serum | IL-12（pg/ml） | 253.10±53.80 | 107.79±43.20 | CCMD-3 | 19/25 | 11/15 | 9.2±2.3 | 8.8±2.7 | 7 |  |
|  |  |  |  | TNF-α（pg/ml） | 627.881±175.122 | 304.475±87.793 |  |  |  |  |  |  |  |
| Cheng,Y.H. | 2010 | China | serum | BDNF（ng/ml) | 4.26±10.69 | 23.34±6.86 | CCMD-3 | 24/31 | 23/29 | 11.75±4.86 | 14.03±3.5 | 7 |  |
| Cui,X. | 2016 | China | serum | Cor(μg/L) | 27.39±13.83 | 18.35±9.75 | DSM-IV | 43/56 | 22/30 | 9.4±2.9 | 8.9±3.2 | 7 |  |
| Tang,W.H. | 2009 | China | plasma | DA（μg/L) | 143.00±30.45 | 56.48±10.62 | DSM-IV | 21/40 | 20/30 | 9.6±2.1 | 9.8±2.4 | 7 |  |
|  |  |  |  | 5-HT（μg/L) | 47.62±17.53 | 320.54±37.84 |  |  |  |  |  |  |  |
| Liu,C.S. | 2002 | China | plasma | Glu (nmol/ml) | 132.81±4.403 | 90.61±4.899 | DSM-IV | 40/48 | 20/24 | NA | NA | 6 |  |
|  |  |  |  | Asp (nmol/m1) | 67.07±3.412 | 28.43±3.433 |  |  |  |  |  |  |  |

^a^ The data are transformed to Mean ± SD [Standard Deviation].

^b^ The data (Mean ± SD [Standard Deviation]) are combined from multiple groups into a single group.
