# Supplementary material for: Biomarkers and Tourette syndrome: a systematic review and meta-analysis
Source: Front Neurol. 2024 Feb 7;15:1262057. doi: 10.3389/fneur.2024.1262057 (PMC10879287; doi:10.3389/fneur.2024.1262057)
Supplement: Supplementary file 4 [file Table_3.DOCX]

Table S3

Quality assessment of selected items in included studies by Newcastle-Ottawa Scale (NOS).

| First Author | Year | Selection | | | | Comparability  Star5 Star6 | | Exposure | | | | Score |
| --- | --- | --- | --- | --- | --- | --- | --- | --- | --- | --- | --- | --- |
|  |  | Star1 | Star2 | Star3 | Star4 |  |  | Star7 | Star8 | Star9 | Star10 |  |
| Gabbay, V. | 2009 | 1 | 1 | 1 | 1 | 0 | 0 | 1 | 0 | 1 | 1 | 7 |
| RuanY.Y. | 2007 | 1 | 1 | 0 | 1 | 1 | 0 | 1 | 0 | 1 | 0 | 6 |
| Singer,H.S. | 1999 | 1 | 1 | 1 | 1 | 0 | 0 | 1 | 0 | 1 | 1 | 7 |
| Pranzatelli,M.R. | 2017 | 1 | 1 | 0 | 1 | 1 | 0 | 1 | 0 | 1 | 1 | 7 |
| You,H.Z. | 2022 | 1 | 1 | 0 | 1 | 1 | 0 | 1 | 0 | 1 | 1 | 7 |
| Liu,Z. | 2013 | 1 | 1 | 1 | 1 | 1 | 0 | 1 | 0 | 1 | 1 | 8 |
| Weisz,J.L. | 2004 | 1 | 1 | 0 | 1 | 0 | 0 | 1 | 0 | 1 | 1 | 6 |
| Cheng,Y.H. | 2012 | 1 | 1 | 1 | 1 | 1 | 0 | 1 | 0 | 1 | 1 | 8 |
| Ruan,Y.Y. | 2009 | 1 | 1 | 0 | 1 | 1 | 0 | 1 | 0 | 1 | 1 | 7 |
| Tang.H.X. | 2014 | 1 | 1 | 0 | 1 | 1 | 0 | 1 | 0 | 1 | 0 | 6 |
| Zhang,B. | 2012 | 0 | 1 | 0 | 1 | 1 | 0 | 1 | 0 | 1 | 1 | 6 |
| Müller,N. | 2000 | 1 | 1 | 1 | 1 | 1 | 0 | 1 | 0 | 1 | 1 | 8 |
| Rizzo.R | 2005 | 1 | 1 | 0 | 1 | 1 | 0 | 1 | 0 | 1 | 1 | 7 |
| Cheng,Y.H. | 2010 | 1 | 1 | 1 | 1 | 1 | 0 | 1 | 0 | 1 | 1 | 8 |
| Landau,Y.E. | 2012 | 1 | 1 | 0 | 1 | 1 | 0 | 1 | 0 | 1 | 1 | 7 |
| Li,H.H. | 2018 | 1 | 1 | 1 | 1 | 1 | 0 | 1 | 0 | 1 | 1 | 8 |
| Li,H.H. | 2017 | 1 | 1 | 0 | 1 | 1 | 0 | 1 | 0 | 1 | 1 | 7 |
| Martino,D. | 2005 | 1 | 1 | 0 | 1 | 0 | 0 | 1 | 0 | 1 | 1 | 6 |
| Li,E. | 2015 | 1 | 1 | 0 | 1 | 1 | 0 | 1 | 0 | 1 | 1 | 7 |
| Zhang,F.H. | 2002 | 1 | 1 | 0 | 1 | 0 | 0 | 1 | 0 | 1 | 1 | 6 |
| Li,W.Q. | 2022 | 1 | 1 | 0 | 1 | 1 | 0 | 1 | 0 | 1 | 1 | 7 |
| Ji,W.D. | 2004 | 1 | 1 | 1 | 1 | 0 | 0 | 1 | 0 | 1 | 1 | 7 |
| Zhang,J.Z. | 2014 | 1 | 1 | 0 | 1 | 1 | 0 | 1 | 0 | 1 | 1 | 7 |
| Zheng,J.B. | 2021 | 1 | 1 | 0 | 1 | 1 | 0 | 1 | 0 | 1 | 1 | 7 |
| Liu,Z.S. | 1995 | 1 | 1 | 0 | 1 | 0 | 0 | 1 | 0 | 1 | 1 | 6 |
| Li，J. | 2013 | 1 | 1 | 1 | 1 | 1 | 0 | 1 | 0 | 1 | 1 | 8 |
| He,F. | 2014 | 1 | 1 | 1 | 1 | 1 | 0 | 1 | 0 | 1 | 1 | 8 |
| Ji，J.P. | 2011 | 1 | 1 | 1 | 1 | 1 | 0 | 1 | 0 | 1 | 1 | 8 |
| Dong,L.X. | 2009 | 1 | 1 | 0 | 1 | 1 | 0 | 1 | 0 | 1 | 1 | 7 |
| Li,N. | 2013 | 1 | 1 | 0 | 1 | 0 | 0 | 1 | 0 | 1 | 1 | 6 |
| Lu,Y. | 2007 | 1 | 1 | 0 | 1 | 1 | 0 | 1 | 0 | 1 | 1 | 7 |
| Liu，H.Z. | 2005 | 1 | 1 | 0 | 1 | 1 | 0 | 1 | 0 | 1 | 1 | 7 |
| Li,X.P. | 2014 | 1 | 1 | 0 | 1 | 1 | 0 | 1 | 0 | 1 | 1 | 7 |
| Hou,C. | 2020 | 1 | 1 | 0 | 1 | 1 | 0 | 1 | 0 | 1 | 1 | 7 |
| Xiao,G.H. | 2008 | 1 | 1 | 0 | 1 | 1 | 0 | 1 | 0 | 1 | 1 | 7 |
| Yang,G.F. | 2005 | 1 | 1 | 0 | 1 | 1 | 0 | 1 | 0 | 1 | 1 | 7 |
| Hu,L.J. | 2009 | 1 | 1 | 0 | 1 | 0 | 0 | 1 | 0 | 1 | 1 | 6 |
| Wang,J.G. | 2012 | 1 | 1 | 0 | 1 | 1 | 0 | 1 | 0 | 1 | 1 | 7 |
| Zhang,L.Y. | 2010 | 1 | 1 | 0 | 1 | 1 | 0 | 1 | 0 | 1 | 1 | 7 |
| Tang,H.L. | 2003 | 1 | 1 | 0 | 1 | 1 | 0 | 1 | 0 | 1 | 1 | 7 |
| Zhao,P. | 2021 | 1 | 1 | 0 | 1 | 1 | 0 | 1 | 0 | 1 | 1 | 7 |
| Wang,Y.M. | 2022 | 1 | 1 | 0 | 1 | 1 | 0 | 1 | 0 | 1 | 1 | 7 |
| Wen,X.M. | 2012 | 1 | 1 | 0 | 1 | 1 | 0 | 1 | 0 | 1 | 1 | 7 |
| Gao,C. | 2016 | 1 | 1 | 0 | 1 | 1 | 0 | 1 | 0 | 1 | 1 | 7 |
| Yu,W.J. | 2015 | 1 | 1 | 0 | 1 | 1 | 0 | 1 | 0 | 1 | 1 | 7 |
| Yu,W.J. | 2019 | 1 | 1 | 0 | 1 | 1 | 0 | 1 | 0 | 1 | 1 | 7 |
| Kang,B. | 2019 | 1 | 1 | 0 | 1 | 1 | 0 | 1 | 0 | 1 | 1 | 7 |
| Liu,L. | 2013 | 1 | 1 | 0 | 1 | 1 | 0 | 1 | 0 | 1 | 1 | 7 |
| Gao,C. | 2019 | 1 | 1 | 0 | 1 | 1 | 0 | 1 | 0 | 1 | 1 | 7 |
| Wu,D.S. | 2010 | 1 | 1 | 0 | 1 | 1 | 0 | 1 | 0 | 1 | 1 | 7 |
| Wang,A.Z. | 2022 | 1 | 1 | 0 | 1 | 1 | 0 | 1 | 0 | 1 | 1 | 7 |
| Zhang,X.Q. | 2014 | 1 | 1 | 0 | 1 | 1 | 0 | 1 | 0 | 1 | 1 | 7 |
| Chen,X.R. | 2019 | 1 | 1 | 0 | 1 | 1 | 0 | 1 | 0 | 1 | 1 | 7 |
| Tan,Z.B. | 2016 | 1 | 1 | 0 | 1 | 1 | 0 | 1 | 0 | 1 | 1 | 7 |
| Zhang,S. | 2008 | 1 | 1 | 0 | 1 | 1 | 0 | 1 | 0 | 1 | 1 | 7 |
| Mao,Y.Y. | 2008 | 1 | 1 | 0 | 1 | 1 | 0 | 1 | 0 | 1 | 1 | 7 |
| Chen,Y.H. | 2010 | 1 | 1 | 1 | 1 | 0 | 0 | 1 | 0 | 1 | 1 | 7 |
| Cui,X. | 2016 | 1 | 1 | 0 | 1 | 1 | 0 | 1 | 0 | 1 | 1 | 7 |
| Tang,W.H. | 2009 | 1 | 1 | 0 | 1 | 1 | 0 | 1 | 0 | 1 | 1 | 7 |
| Liu,C.S. | 2002 | 1 | 1 | 0 | 1 | 0 | 0 | 1 | 0 | 1 | 1 | 6 |
